# Aggregation‐Induced Dual Phosphorescence from (*o*‐Bromophenyl)‐Bis(2,6‐Dimethylphenyl)Borane at Room Temperature

**DOI:** 10.1002/chem.202200525

**Published:** 2022-04-08

**Authors:** Zhu Wu, Fabian Dinkelbach, Florian Kerner, Alexandra Friedrich, Lei Ji, Vladimir Stepanenko, Frank Würthner, Christel M. Marian, Todd B. Marder

**Affiliations:** ^1^ Institut für Anorganische Chemie and Institute for Sustainable Chemistry & Catalysis with Boron Julius-Maximilians-Universität Würzburg Am Hubland 97074 Würzburg Germany; ^2^ Institut für Theoretische Chemie und Computerchemie Heinrich-Heine-Universität Düsseldorf Universitätsstr. 1 40225 Düsseldorf Germany; ^3^ Frontiers Science Center for Flexible Electronics (FSCFE) & Shaanxi Institute of Flexible Electronics (SIFE) Northwestern Polytechnical University Xi An Shi 127 West Youyi Road 710072 Xi'an P. R. China; ^4^ Institut für Organische Chemie and Center for Nanosystems Chemistry Julius-Maximilians-Universität Würzburg Am Hubland 97074 Würzburg Germany

**Keywords:** AIE, luminescence, phosphorescence, triarylborane, triplet

## Abstract

Designing highly efficient purely organic phosphors at room temperature remains a challenge because of fast non‐radiative processes and slow intersystem crossing (ISC) rates. The majority of them emit only single component phosphorescence. Herein, we have prepared 3 isomers (*o*, *m*, *p*‐bromophenyl)‐bis(2,6‐dimethylphenyl)boranes. Among the 3 isomers (***o***‐, ***m***‐ and ***p***‐**BrTAB**) synthesized, the *ortho*‐one is the only one which shows dual phosphorescence, with a short lifetime of 0.8 ms and a long lifetime of 234 ms in the crystalline state at room temperature. Based on theoretical calculations and crystal structure analysis of ***o***‐**BrTAB**, the short lifetime component is ascribed to the T_1_^M^ state of the monomer which emits the higher energy phosphorescence. The long‐lived, lower energy phosphorescence emission is attributed to the T_1_^A^ state of an aggregate, with multiple intermolecular interactions existing in crystalline ***o***‐**BrTAB** inhibiting nonradiative decay and stabilizing the triplet states efficiently.

## Graphical abstract

**Bromo‐triarylborane** ***o*** **‐BrTAB** exhibits dual phosphorescence at room temperature; a short‐lived higher energy emission originates from single molecules (T_1_^M^) whereas the long‐lived component at lower energy results from an aggregate (T_1_^A^). Under 365 nm UV light irradiation, crystalline ***o*** **‐BrTAB** emits bright pale blue light (fluorescence). After turning off the light, a persistent yellow afterglow remains for 2 s.

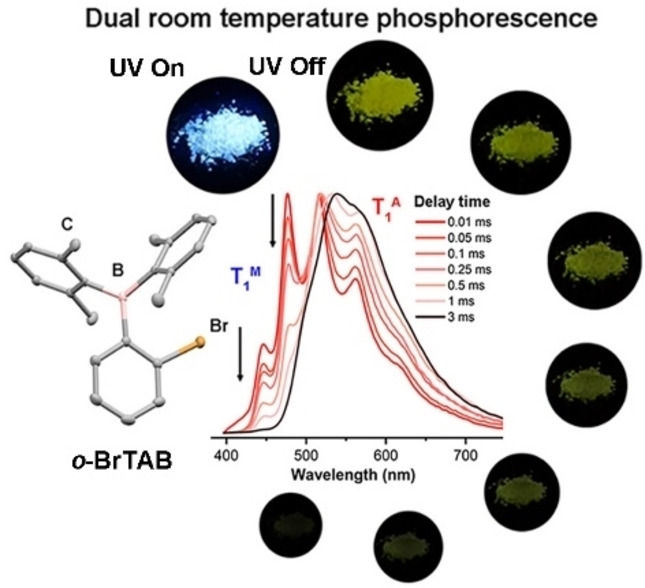

## Introduction

Purely organic phosphors have received considerable research interest in optoelectronic devices,^[1]^ bioimaging, molecular sensing,^[3]^ and security printing,^[4]^ due to efficient utilization of their triplet states. So far, most luminophores displaying room temperature phosphorescence (RTP) are restricted to inorganics containing noble transition metals such as iridium (Ir) and platinum (Pt), as transition‐metal complexes are characterized by strong spin‐orbit coupling (SOC) induced by heavy metal ions and an intrinsic conformational rigidity which can theoretically harvest 100 % of the electrically generated singlet and triplet excitons.^[5]^ In addition, the phosphorescence emission spectra and quantum efficiencies can be tuned by modification of the ligand system. On the other hand, purely organic phosphors generally show inefficient intersystem crossing (ISC) and slow radiative decay rates from the lowest triplet state (T_1_) to the ground singlet state (S_0_).^[6]^ In addition, the long‐lived triplet excitons in metal‐free luminophores can interact with environmental quenchers such as O_2_, which quench the phosphorescence to a great extent.^[7]^ In order to achieve RTP, one prerequisite is to enhance SOC to accelerate the ISC process, typically by utilizing heteroatoms, heavy atoms, or particular functional groups containing heteroatoms with lone pairs such as C=O, C=S or NR_2_.^[8]^ At the same time, it is also important to suppress nonradiative pathways and to isolate the chromophores from oxygen by host‐guest doping,^[9]^ crystallization,^[10]^ or incorporation in a polymer matrix or on carbon dots.^[11]^ Unlike trapped organic phosphors which emit only single component phosphorescence,^[12]^ dual phosphorescence emission results from two different triplet states and was observed previously in a frozen glass matrix at low temperature.^[13]^ More recently, dual room temperature phosphorescence (DRTP) in aggregated states was reported by several groups. For example, Tang's group developed a single‐molecule white light phosphor which emits from both T_1_ and T_2_ states at room temperature.^[14]^ Huang et al. reported a series of indole derivatives which show DRTP via inter‐/intramolecular charge transfer.^[15]^ Zhang et al. designed a number of D‐*sp* ^3^‐linker‐A‐type triphenylamine (TPA) luminophores, which showed a TPA‐localized triplet state T_1_^L^ and an acceptor centered triplet state T_1_^H^ simultaneously in a PMMA film.^[16]^ Ma's group observed dual phosphorescence from pyridine‐modified carbazole derivatives originating from T_1_ and T_1_* states.^[17]^ Although several DRTP luminophores have been reported, research on this topic and the types of DRTP systems are still quite limited,^[18]^ and there is no reliable design concept and strategy to achieve DRTP luminophores with high performance.

**Figure 1:**
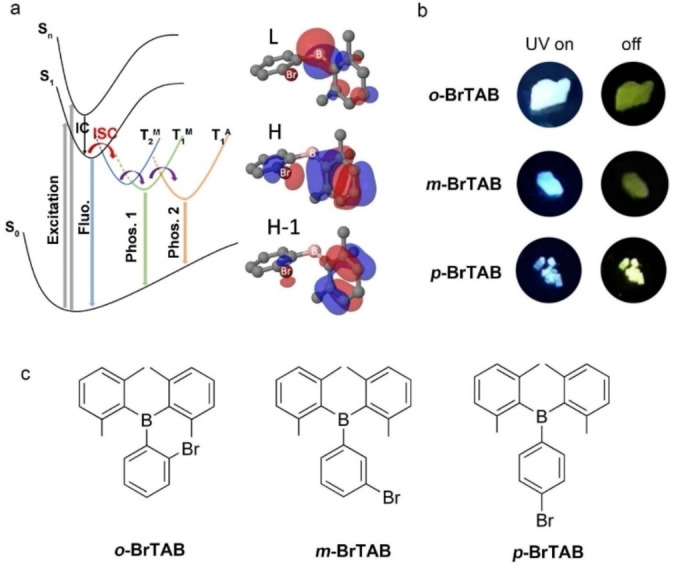
(a) Proposed mechanism of the dual phosphorescent emission in ***o***‐**BrTAB** and essential parts of the molecular orbitals involved in the transitions; T_1_^M^ and T_2_^M^ represent monomer states, T_1_^A^ an aggregate state; (b) afterglow photographs taken before and after irradiation (λ_ex_=365 nm) under ambient conditions; (c) molecular structures of the three isomeric compounds described in the paper.

Based on our long standing interest in 3‐coordinate organoboron materials and their optical and electronic properties,[^19^, ^20^] we successfully designed persistent triarylborane phosphors with efficient ISC via (σ, B p)→(π, B p) transitions.^[21]^ However, it seemed likely that the SOC and, thus, the ISC rate constant could be improved. It is proposed that introducing heavy atoms (Br or I) into existing persistent RTP luminophores to enhance the SOC may greatly improve the performance of RTP phosphors. Br or I atoms can also provide multiple efficient intermolecular interactions to restrict nonradiative pathways, which is crucial to stabilize triplet states.^[22]^ More importantly, the dense crystalline matrix provides a favorable environment for delocalization of the excitation and the formation of energy dispersed band structures.^[23]^ Herein, we introduced the heavy atom Br into phenyl‐bis(2,6‐dimethylphenyl)borane at different positions on the phenyl ring to obtain 3 isomers (***o***‐, ***m***‐ and ***p***‐**BrTAB**, Figure 1c). Interestingly, (2‐bromophenyl)bis(2,6‐dimethylphenyl)borane (***o***‐**BrTAB**) exhibits DRTP in the crystalline state under ambient conditions (Figure 1b). The faster, higher energy phosphorescence ranging from 430 to 490 nm with a short lifetime of 0.8 ms is ascribed to the T_1_^M^ state of the monomer while the long‐lived, lower energy phosphorescence emission in the range of 490–700 nm with a lifetime of up to 234 ms is attributed to the T_1_^A^ state of an aggregate in the crystalline material.

## Results and Discussion

**Figure 2:**
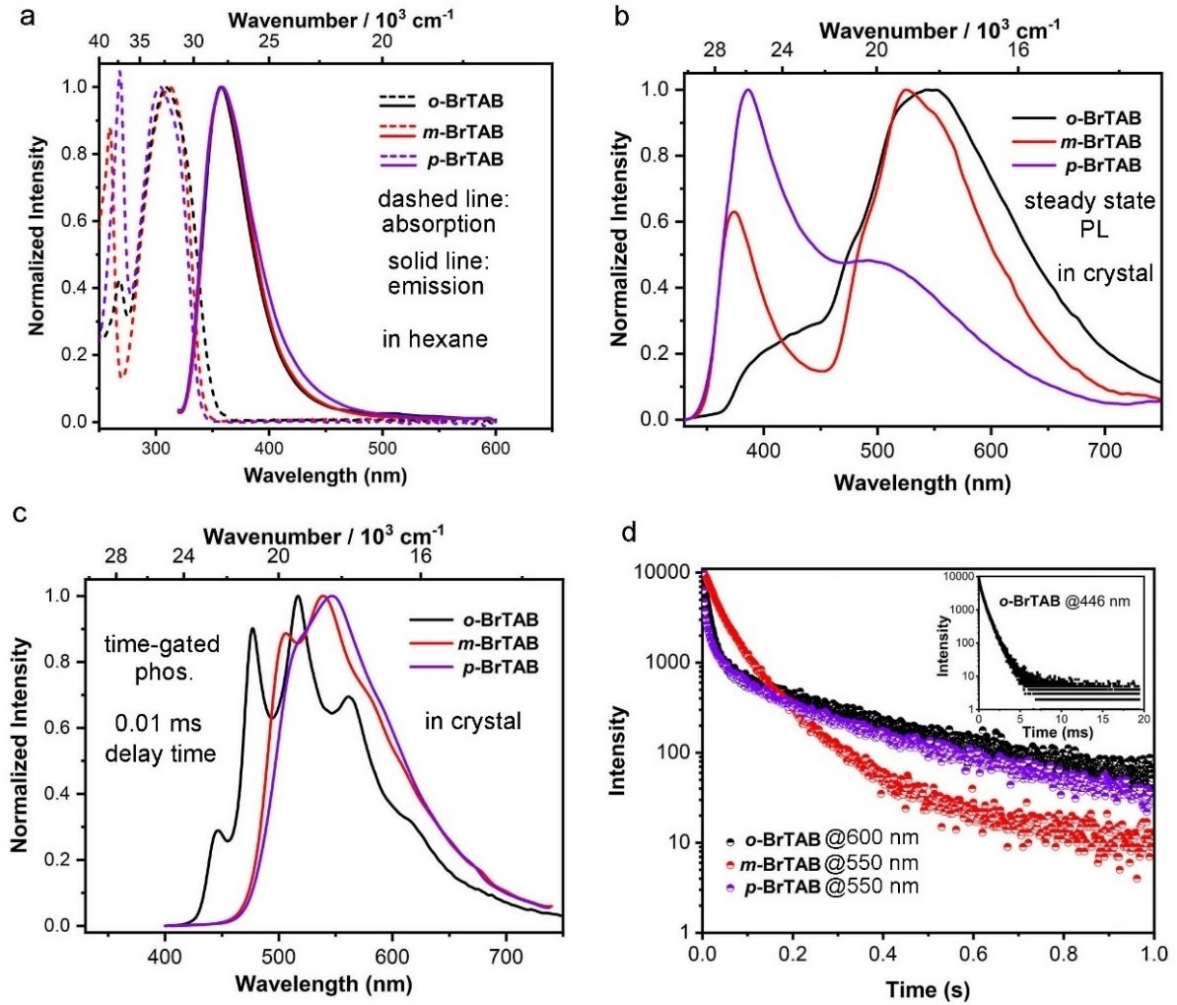
Normalized (a) UV/Vis absorption and fluorescence in hexane, (b) PL and (c) time‐gated (0.01 ms delay) phosphorescence spectra of crystalline ***o***‐, ***m***‐ and ***p*** **‐BrTAB** (*λ*_ex_=305 nm). (d) Decays of the phosphorescence (600 nm for ***o*** **‐BrTAB** and 550 nm for ***m***‐ and ***p*** **‐BrTAB**) in the crystalline state. Insert: decay of the phosphorescence (446 nm) of crystalline ***o*** **‐BrTAB**. All above measurements were conducted at room temperature in air.

The compound ***o***‐**BrTAB** was synthesized^[24]^ by reaction of the Grignard reagent (2,6‐Me_2_‐C_6_H_3_)MgBr with *o*‐Br‐C_6_H_4_BF_3_K, whereas ***m***‐ and ***p***‐**BrTAB** were prepared by the reaction of bis(2,6‐dimethylphenyl)fluoroborane with the respective aryllithium species generated by mono lithium‐halogen exchange of *m*‐ or *p*‐dibromobenzene with *n*‐BuLi (for synthetic details and characterization data, see the Supporting Information). All three compounds showed absorption bands between 270 and 350 nm in hexane which are attributed to B←π transitions with extinction coefficients of *ϵ*=10000‐16000 M^−1^cm^−1^ (Figure 2a and Table 1). Placement of the Br atoms at the *ortho*, *meta* and *para* positions of the phenyl group had no obvious effect on the molecular energy levels of the three triarylboranes.

**Table 1:** Photophysical properties of ***o‐***, ***m‐*** and ***p‐*** **BrTAB** in hexane and the crystalline state at RT, and in frozen methylcyclohexane and in the crystalline state at 77 K.

Compound	State [T]	*λ*_abs_ [nm]	*Ε* [_M_^−1^cm^−1^]	*Φ*_PL_ [*%*]	*λ*_f_ [nm]	*Φ*_f_ [*%*]	*τ*_f_ ns	*τ*_0_^f^ [ns]	*k*_r_^f^ [s^−1^]	*λ*_p_ [nm]	*Φ*_p_ [%]	*τ*_p_ (T_1_^M^) [ms]	*τ*_p_ (T_1_^A^) [ms]
***o*** **‐BrTAB**	Solution (RT)^[a]^	312	10900	3.5	358	3.5	2.8	80	1*10^7^	nd^[b]^		nd	nd
	Crystalline (RT)^[c]^			1.5	–^[d]^	0.2	3.2	8*10^3^	1*10^5^	446, 477, 517, 562^[e]^	1.3	0.3 (34 %), 0.8 (66 %)	13 (22 %), 234 (78 %)
	Crystalline (77 K)	–		–	–		–	–		431, 455		0.3 (28 %), 2.1 (72 %)	161 (42 %), 453 (58 %)
	Frozen glass (77 K)^[f]^	–		–	–					457		23 (57 %), 92 (43 %)	–
***m*** **‐BrTAB**	Solution (RT)^[a]^	310	11300	1.4	358	1.4	2.3	164	6*10^6^	nd		–	nd
	Crystalline (RT)^[c]^	–		3.8	374	0.7	6.0	3*10^3^	3*10^5^	506, 539	3.1	–	64 (67 %), 215 (33 %)
	Crystalline (77 K)			–	374		–	–		430, 458, 483, 510		51 (54 %), 153 (45 %)	45 (28 %), 512 (72 %)
	Frozen glass (77 K)^[f]^	–		–	–		–	–		438		154 (55 %), 486 (45 %)	–
***p*** **‐BrTAB**	Solution (RT)^[a]^	304	15200	1.2	358	1.2	1.7	77	1*10^7^	nd		–	nd
	Crystalline (RT)^[c]^	–		4.4	386	2.4	2.9	121	8*10^6^	547	2.0	–	90 (30 %), 378 (70 %)
	Crystalline (77 K)	–		–	386			–		427, 458, 510		18 (69 %), 59 (31 %)	166 (19 %), 581 (81 %)
	Frozen glass (77 K)^[f]^	–		–	367		–	–		438		53 (48 %), 201 (52 %)	–

[a] Measured in hexane at room temperature (RT); [b] not detected (nd); [c] measured in the crystalline state at RT; [d] The maximum fluorescence emission wavelength of ***o*** **‐BrTAB** is estimated to occur at ca. 400–405 nm, but cannot be more accurately determined due to the overlap of fluorescence and phosphorescence; [e] 446 and 477 nm are ascribed to **T_1_** ^**M**^ and 517 and 562 nm are ascribed to **T_1_** ^**A**^; [f] measured in frozen methylcyclohexane at 77 K. For the phosphorescence lifetimes, the value given in % in parentheses is the contribution to a bi‐exponential fit of the decay.

The spectra are composed of two absorption bands, where the lower energy band, around 300 nm, is more intense than the one at 260–270 nm. DFT/MRCI (multireference configuration interaction) calculations (Figure S10) show that the band at 300 nm is an overlay of the S_0_→S_1_ and S_0_→S_4_ absorptions, while the band at higher energy arises from the S_0_→S_6_ absorption. They also reveal that irradiation of the compounds with UV light in the 310–320 nm wavelength regime predominantly populates the S_1_ state. The experimental fluorescence spectra of the 3 isomers in solution almost overlap, with emission maxima at 358 nm. Our calculations show the onsets of the fluorescence to occur at 348 nm for ***o***‐**BrTAB**, 328 nm for ***m***‐**BrTAB** and 324 nm for ***p***‐**BrTAB**, while experimentally, the onset is located at 328 nm for all three compounds. The computed fluorescence rate constants for ***o***‐, ***m***‐, and ***p***‐**BrTAB** are 1×10^7^, 2×10^7^ and 2×10^7^ s^−1^, respectively, (Table S3) while their corresponding experimental fluorescence rate constants are 1×10^7^, 6×10^6^ and 1×10^7^ s^−1^, respectively, showing good agreement between calculated and experimental values (Table 1 and Table S3).

**Figure 3:**
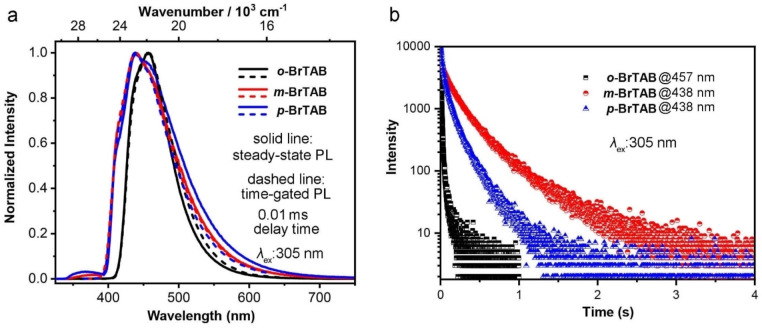
(a) Normalized photoluminescence (solid line) and time‐gated phosphorescence (dashed line) spectra and (b) decays of the phosphorescence of ***o‐***, ***m‐*** and ***p***‐**BrTAB** at their maximum emission wavelength in a frozen methylcyclohexane glass at 77 K (*λ*_ex_=305 nm).

We also measured the PL emission spectra in a frozen methylcyclohexane optical glass at 77 K (Figure 3). Compared to ***m***‐ and ***p***‐**BrTAB**, which still show some residual fluorescence at higher energies (330‐400 nm), only phosphorescence with a lifetime of 23 ms (57 %) was observed in the spectrum of ***o***‐**BrTAB**, which indicates that ISC is very efficient and the phosphorescence quantum yield is much higher than that of fluorescence. Note that, as indicated in the footnote to Table 1, % values in parentheses are the contributions to a bi‐exponential fit of the decays. Comparison of both experimental and computed fluorescence rate constants (ca. 10^7^ s^−1^) with the fastest ISC rate constants for ***o***‐, ***m***‐, and ***p***‐**BrTAB** obtained from our theoretical calculations (see below) of 1×10^10^, 9×10^8^ and 3×10^8^ s^−1^ (Table S3), respectively, supports the above observations at 77 K in the frozen matrices. Thus, in the bromo‐substituted triarylboranes, most of the excited state population is transferred to the triplet manifold, especially in ***o***‐**BrTAB**. The heavy atom effect of Br on the monomer phosphorescence radiative lifetimes is in the order ***o***‐**BrTAB**> ***p***‐**BrTAB**> ***m***‐**BrTAB** (Table S3), in agreement with the experimental trends listed in Table 1. The absence of DRTP in the frozen glass at 77 K suggests that the longer‐lived phosphorescence component must originate from an aggregated state.

**Figure 4:**
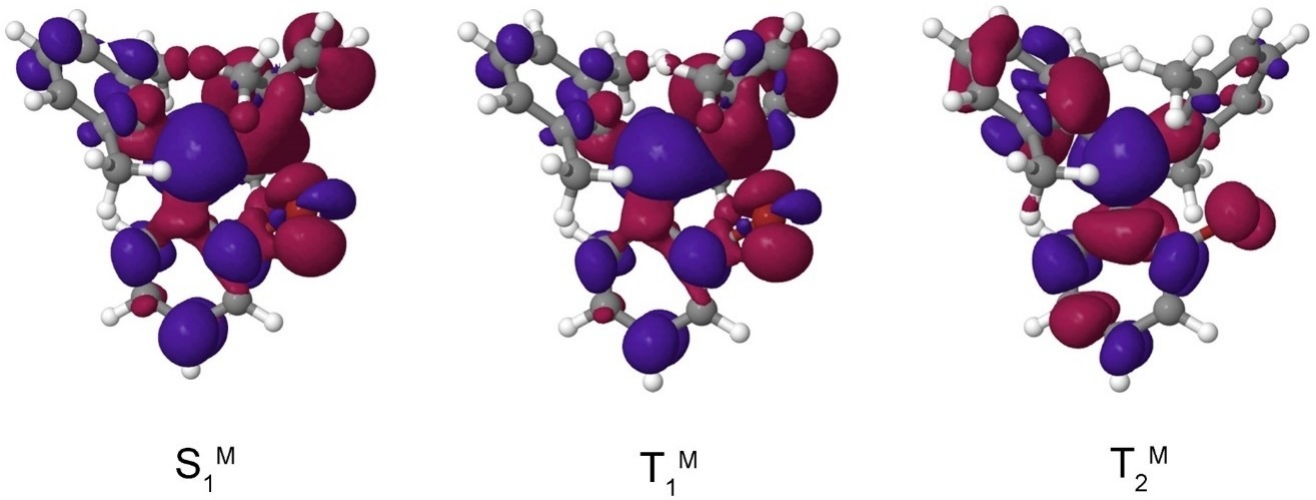
Difference of electron density distributions in the S_1_^M^, T_1_^M^ and T_2_^M^ states of the ***o*** **‐BrTAB** monomer with regard to the electronic ground state, S_0_^M^, at the S_1_^M^ geometry. Red areas indicate a loss of electron density upon excitation, blue areas a gain. Note the reorientation of the p orbital hole on the bromine atom when proceeding from S_1_^M^ to T_2_^M^.

Recently, Sarkar, Hendrickson et al. reported on the three isomeric *o*‐, *m*‐, and *p*‐bromobenzaldehydes.^[25]^ When Br is *ortho* to the aldehyde, SOC is greatly enhanced, consistent with our observations. Looking at Table S3, the heavy Br atom effect leads to efficient ISC for all three compounds. However, while the fluorescence rate constant has the same order of magnitude (10^7^ s^−1^) for all three isomers, the ISC rate constants decrease in the order ***o***‐> ***m***‐> ***p***‐**BrTAB**. For ***p***‐**BrTAB** and ***m***‐**BrTAB**, ISC is 15 and 45 times faster, respectively, than fluorescence, so that residual fluorescence might be expected from those 2 isomers. For ***o***‐**BrTAB**, however, ISC is 1000 times faster than fluorescence, so nearly all excited molecules rapidly form triplet states. As can be seen in the difference densities in Figures S13‐15, bromine is involved more strongly in the excitation the closer it is located to the boron center. Following El‐Sayed's rule,^[27]^ a change in orbital character is required for a fast ISC process. This orbital change is visible in all three isomers, but most dominant in the ***o***‐**BrTAB** compound, where the Br p‐orbital changes its orientation moving from the S_1_ to the T_2_ state (Figure 4) causing the squared SOCME to increase markedly to 18640 cm^−2^. Despite the similarity of the electron distributions in both states (Figure 4), even the S_1_^M^ and T_1_^M^ states experience substantial mutual SOC in ***o***‐**BrTAB**. With a squared sum of SOCMEs of 25 cm^−2^, the S_1_^M^→T_1_^M^ transition is significantly faster (*k*_ISC_=2×10^9^ s^−1^) than in the unsubstituted compound. Nevertheless, with a rate constant of ca. 1×10^10^ s^−1^, the S_1_^M^→T_2_^M^ ISC is five times faster than the direct S_1_^M^→T_1_^M^ ISC. Besides ISC, internal conversion (IC) plays an important role for the population of the emissive T_1_ state from higher triplet states. In ***o***‐**BrTAB** we found a conical intersection allowing a very efficient population transfer from T_2_ to T_1_ without the necessity to surmount a large energy barrier in the process.

In ***m***‐**BrTAB**, the largest SOCMEs arise between the S_1_^M^→T_2_^M^ and S_1_^M^→T_3_^M^ pair of states. Both target states are similar in character except that the bromine involvement is more pronounced in the T_3_^M^ state, yielding a rate constant of 9×10^8^ s^−1^ for the S_1_^M^→T_3_^M^ ISC in this isomer. In ***p***‐**BrTAB**, the bromine substitution primarily enhances the probabilities of the S_1_^M^→T_1_^M^ and S_1_^M^→T_3_^M^ ISCs. Here we find the T_2_^M^ state to be more similar in character to the S_1_^M^ state than the T_1_^M^ state. The fastest ISC is found for the S_1_^M^→T_3_^M^ channel with *k*_ISC_=3×10^8^ s^−1^. A comparison of the ISC rate constants with the fluorescence rate constants of ca. 10^7^ s^−1^ (Table S3) suggests that the fluorescence quantum yield is low and that most of the excited state population is transferred to the triplet manifold in the bromo‐substituted triarylboranes, in particular in the ***o***‐**BrTAB** isomer, which is consistent with the low fluorescence intensities observed in a frozen glass at 77 K.

The phosphorescence rate constant of T_1_ depends essentially on three factors: the magnitude of the SOC matrix element (SOCME) between T_1_ and singlet states S_n_, the energy difference between T_1_ and S_n_, and the brightness of the S_n_→S_0_ transition. Interference effects aside, the larger the T_1_‐S_n_ SOC, the smaller the T_1_‐S_n_ energy difference, and the larger the S_n_→S_0_ transition dipole, the larger the phosphorescence probability.^[26]^ Because of the large T_1_‐S_2_ SOC and T_1_‐S_4_ SOC, ***o***‐**BrTAB** can borrow substantial intensity from the spin‐allowed bright transitions (S_2_→S_0_ and S_4_→S_0_, respectively). In ***m***‐**BrTAB** and ***p***‐**BrTAB**, SOC between T_1_ and the low‐lying singlet states is small and, therefore, the intensity borrowing is not very efficient. Hence, ***o***‐**BrTAB** is the only compound with a phosphorescence rate constant in the millisecond regime; the other two have rate constants of 5 s (***m***‐**BrTAB**) and 1 s (***p***‐**BrTAB**) according to the calculations.

However, the photoluminescence spectra at room temperature in the crystalline state are much different from the results in solution (Figure 2b). First, in addition to the fluorescence peaks attributed to the monomer excited state at short wavelength, broad peaks between 450 and 750 nm result from phosphorescence. The longer‐lived (phosphorescence) emission lifetimes measured for ***o***‐, ***m***‐ and ***p***‐**BrTAB** at room temperature are 234 (78 %), 64 (67 %) and 378 (70 %) ms, respectively, where the percentages given are those of the larger component of a bi‐exponential fit to the decay curves (Figure 2d). Second, the fluorescence emissions from crystalline samples of ***o**‐*, ***m**‐* and ***p***‐**BrTAB** are all redshifted compared with those in hexane solution. The bathochromic shift of ***p***‐**BrTAB** (2026 cm^−1^) is approximately twice that of ***m***‐**BrTAB** (1194 cm^−1^). Third, ***o***‐**BrTAB** has a larger ratio of phosphorescence to fluorescence intensity, indicating that ISC in ***o***‐**BrTAB** is the most efficient of the three isomers. The time‐gated phosphorescence spectrum of crystalline ***o***‐**BrTAB** at room temperature shows four fine structured bands at 446, 477, 517 and 562 nm, respectively (Figure 2c). The vibrational fine structure is also present in the computed Franck‐Condon (FC) spectrum of the T_1_^M^ emission, though less pronounced. We attribute it to a progression of a vibrational mode with a frequency of 1673 cm^−1^ in the electronic ground state which corresponds to an asymmetric C−C stretching motion of the xylyl ring closest to the Br atom.

The aggregation state of **o**‐**BrTAB** has a large influence on the photoluminescence behavior. A ground solid sample of **o**‐**BrTAB** exhibited multiple small size particles with increased surface area as observed by SEM (Figure S1). The powder X‐ray diffraction pattern of the ground **o**‐**BrTAB** sample shows its crystalline nature (Figure S3). In the ground sample, the exposed surface area is much larger than in the single crystal and, as slower phosphorescence is more sensitive to oxygen quenching, the ratio of phosphorescence to fluorescence dramatically decreased (Figure S2). The longer phosphorescence lifetime decreases significantly from 234 to 191 ms and the quantum yield is too small to be measured. The shorter phosphorescence lifetime also drops slightly to 0.7 ms, further indicating the important role of the aggregation state in the photoluminescence behavior. In addition, **o**‐**BrTAB** was embedded in a poly(methyl methacrylate) (PMMA) matrix at different loading levels. In a highly doped film (40 wt%, Figure S4), the time‐gated phosphorescence emission blueshifts (ca. 600 cm^−1^) compared to that in the crystalline state, and only the short lifetime (0.8 ms) component was detected, while in an even more concentrated PMMA film (60 wt%, Figure S5), the long lifetime (226 ms) component emerged. The experimental results clearly indicate that longer‐lived component of the DRTP is induced by aggregation.

**Figure 5:**
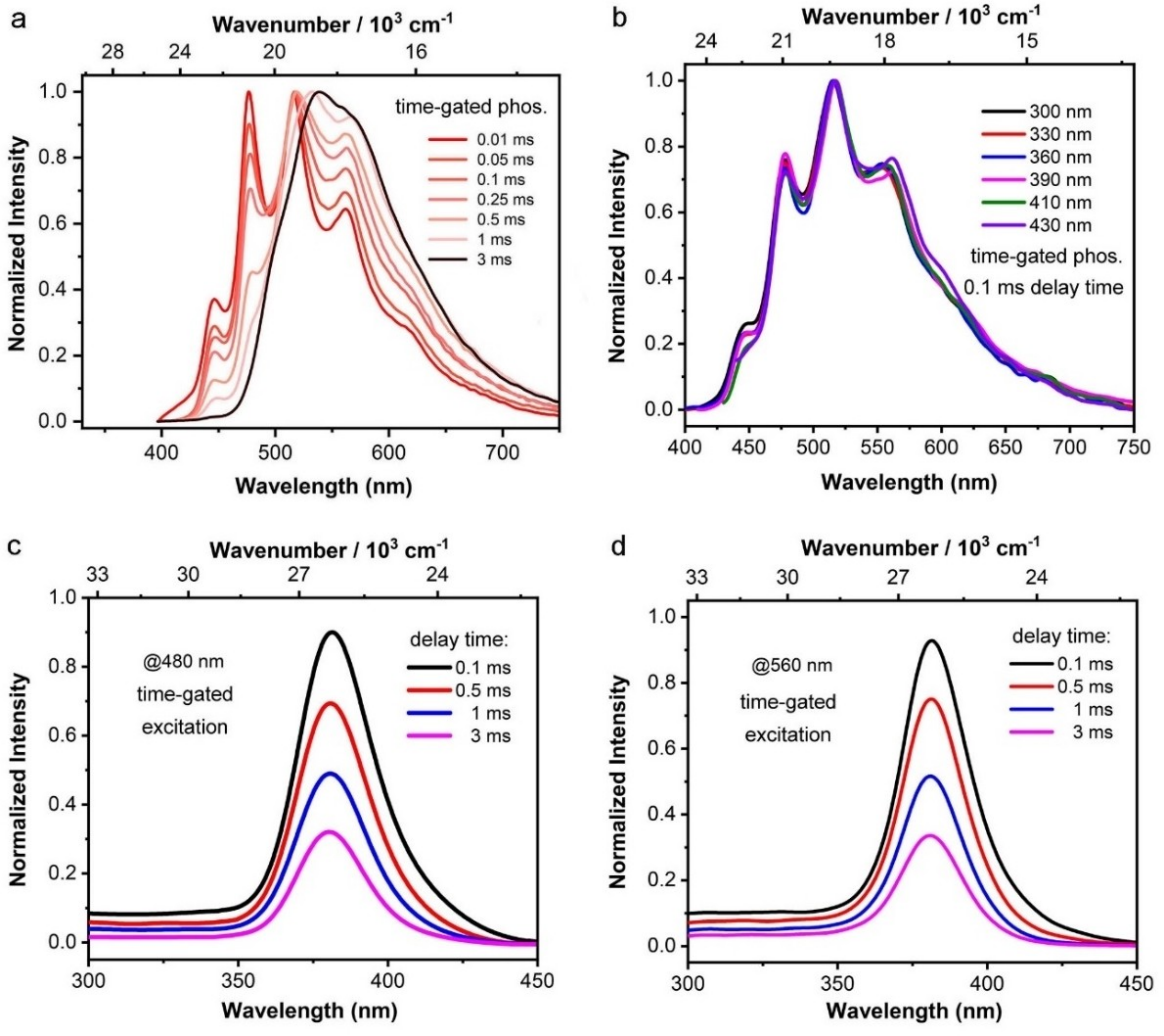
Normalized time‐gated phosphorescence spectra of crystalline ***o*** **‐BrTAB** (a) with different delay times and (b) with 0.1 ms delay at different excitation wavelengths. Time‐gated excitation spectra (c, *λ*_em_=480 nm) and (d, *λ*_em_=560 nm) of crystalline ***o*** **‐BrTAB** at different delay times at room temperature.

**Figure 6:**
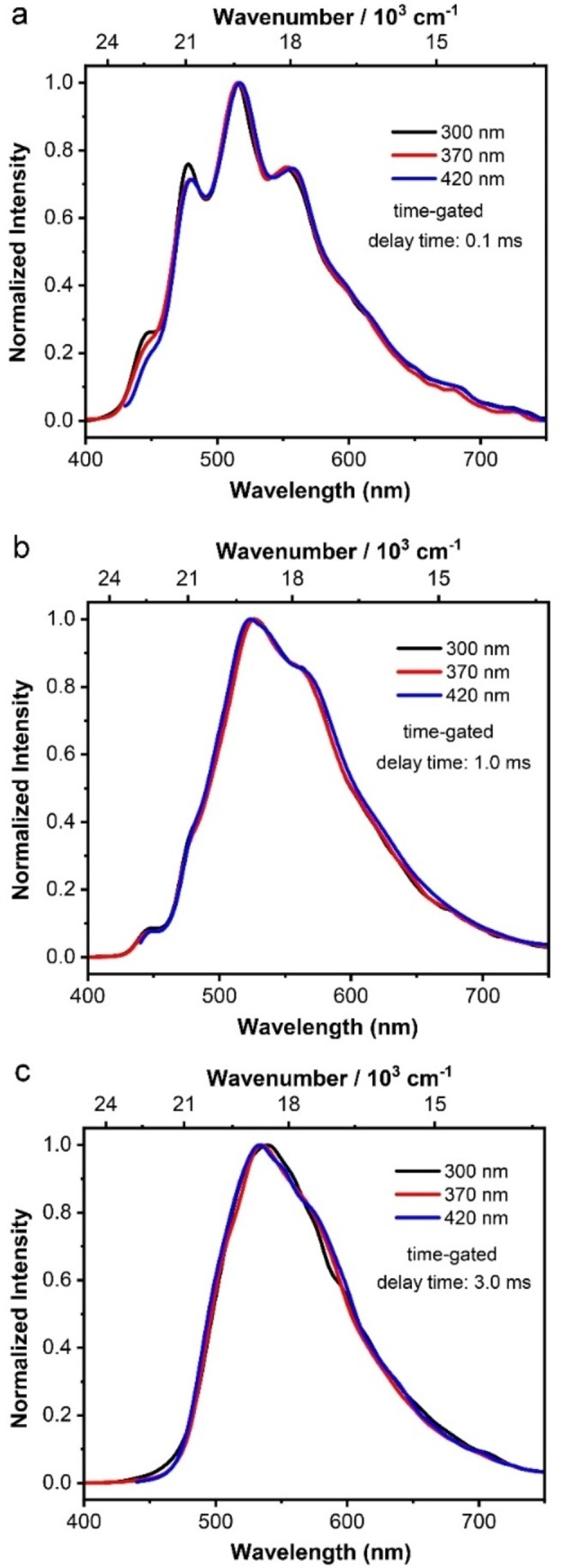
Normalized time‐gated phosphorescence spectra of crystalline ***o***‐**BrTAB** at different excitation wavelengths (*λ*_ex_=300, 370 and 420 nm) and different delay times of (a) 0.1, (b) 1.0, and (c) 3.0 ms.

The DRTP was confirmed by time‐gated phosphorescence spectroscopy of ***o***‐**BrTAB** at room temperature (Figure 5a). Upon increasing the delay time, the intensity of peaks of the shorter wavelength emission components at 446 and 477 nm decreased gradually. When the delay time was set at 3 ms, the short‐lived monomer T_1_^M^ emission of ***o***‐**BrTAB** almost disappeared, and the remaining long lifetime component is ascribed to phosphorescence from the T_1_^A^ state of an aggregate. We performed time‐gated excitation spectroscopy of crystalline ***o***‐**BrTAB** at 480 and 560 nm, respectively (Figures 5c and 5d). Upon increasing the delay time from 0.1 to 3 ms, the overall excitation intensity gradually decreases. We stress that the spectra at each delay time are identical in the range of 300 to 450 nm, indicating that one absorption leads to all excited states. We also measured time‐gated phosphorescence spectra at different excitation wavelengths (Figure 5b). The two triplet excited states always appear at the same time which further indicates that T_1_^M^ and T_1_^A^ originate from the same absorption (S_0_→S_1_ and S_0_→S_n_). Furthermore, we measured the time‐gated phosphorescence spectra of ***o***‐**BrTAB** in the crystalline state at different excitation wavelengths (300, 370 and 420 nm) and different delay times (0.1, 1.0, and 3.0 ms) (Figure 6). When a delay time of 0.1 ms was used to measure the time‐gated spectra of crystalline ***o***‐**BrTAB** at different excitation wavelengths (Figure 6a), the short‐lived component T_1_^M^ and long‐lived component T_1_^A^ are both apparent and the spectra at the three different excitation wavelengths are identical. When a longer delay time of 1.0 ms was used at different excitation wavelengths (Figure 6b), T_1_^M^ and T_1_^A^ also appear with a smaller relative intensity of the short‐lived T_1_^M^ component due to the time‐gating effect. With a 3.0 ms delay time (Figure 6c), the relative intensity of the T_1_^M^ component becomes even smaller, but it is still apparent. These results clearly indicate that the short‐lived T_1_^M^ and long‐lived T_1_^A^ components originate from the same absorption.

**Figure 7:**
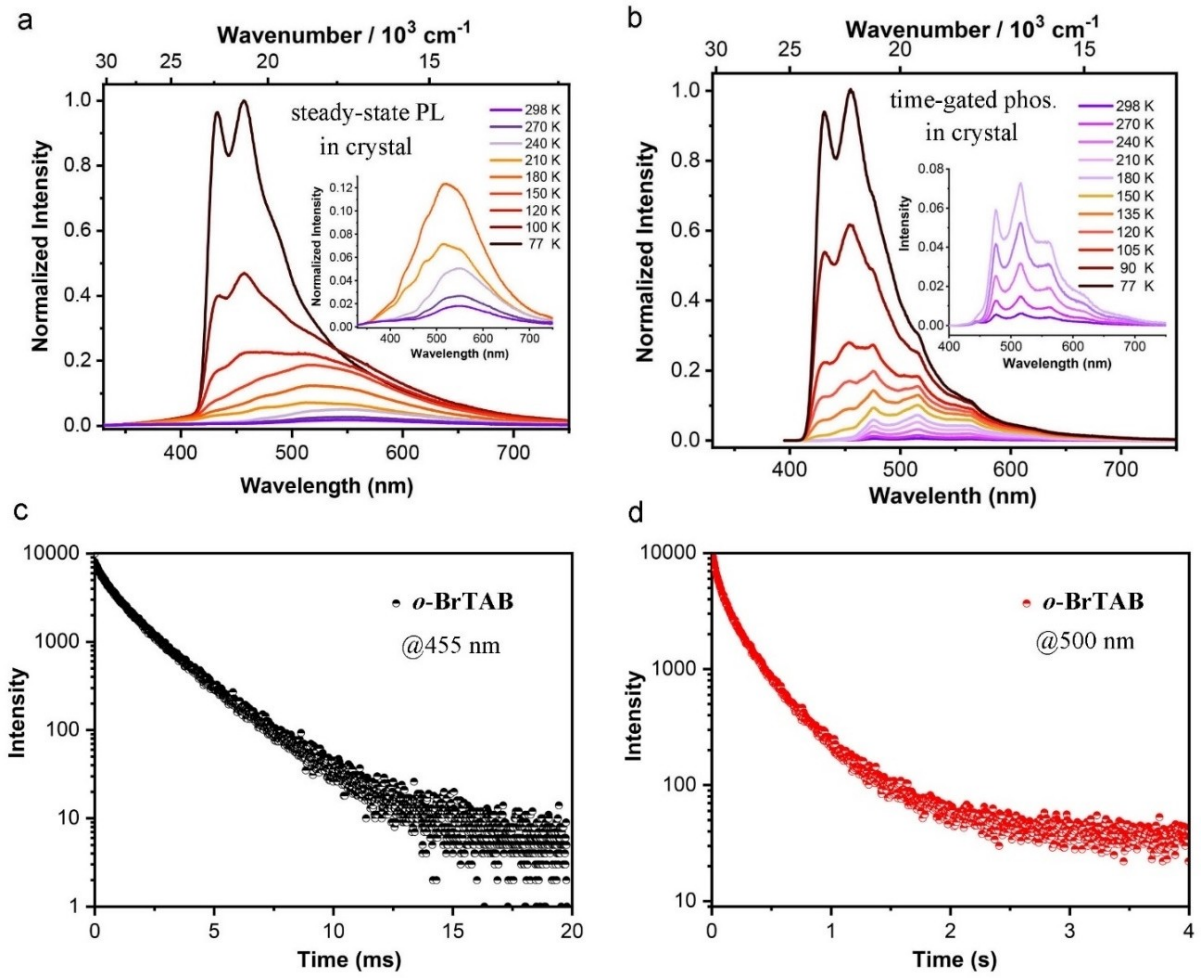
(a) Steady‐state photoluminescence and (b) time‐gated (delay time 0.01 ms) phosphorescence spectra of crystalline ***o‐*** **BrTAB** at different temperatures (*λ*_ex_=305 nm). Decays of the phosphorescence emission (c) (*λ*_em_=455 nm) and (d) (*λ*_em_=500 nm) of crystalline ***o‐*** **BrTAB** at 77 K.

Therefore, we propose that first, the single molecule of ***o***‐**BrTAB** is excited into its S_1_^M^ or S_n_^M^ state. Then it undergoes ISC to T_2_^M^ which undergoes IC to a short‐lived T_1_^M^ state. At the same time, T_1_^M^ evolves into a T_1_^A^ state, emitting more slowly and at lower energy. The process going from T_1_^M^ to T_1_^A^ was confirmed by temperature‐dependent, time‐gated spectroscopic study of crystalline ***o***‐**BrTAB** (Figure 7b). Upon decreasing the temperature from 298 to 77 K, the phosphorescence peaks belonging to T_1_^M^ at 446 and 477 nm blueshift to ca. 431 and 455 nm, respectively, which become dominant at 77 K with a lifetime ca. 2.1 ms. At 77 K, the long‐lived T_1_^A^ emission at longer wavelength still exists with a lifetime of ca. 453 ms (58 %), but in a much lower ratio compared to the short‐lived component. Thus, there is a thermal barrier for the conversion of T_1_^M^ to T_1_^A^, and lowering the temperature makes it harder to cross the barrier.

For crystalline **m**‐ and **p**‐**BrTAB**, we did not observe dual phosphorescence at room temperature, but only at low temperature. At 77 K, new emission peaks at 431 and 455 nm appeared in the time‐gated spectra with the phosphorescence lifetimes of crystalline **m**‐ and **p**‐**BrTAB** of 51 (54 %) and 18 (69 %) ms, respectively, being much shorter than the lifetimes 512 (72 %) and 581 (81 %) ms of the longer‐wavelength emissions (Figures S6–S9). As the strength of intermolecular interactions is especially distinct between the three compounds at room temperature, only ***o***‐**BrTAB** exhibits strong intermolecular interactions, effectively suppressing the nonradiative decay rate *k*_nr_, which plays an important role in stabilizing the triplet states. Hence, a nonradiative decay channel is opened for ***m***‐ and ***p***‐**BrTAB** going from 77 K to RT. In ***o***‐**BrTAB**, the radiative channel can compete with the nonradiative one even at RT. This may be the reason that dual phosphorescence is only observed in ***o***‐**BrTAB** at room temperature and a detailed discussion of intermolecular interactions is provided in the crystal structure analysis section (see below).

To explain the experimentally observed dual phosphorescence, we first searched for the most probable pathways from the singlet to the triplet states of the monomer. For the unsubstituted compound phenyl‐bis(2,6‐dimethylphenyl)borane, we have already shown that the S_1_→T_1_ transition is slower than S_1_→T_2_.^[21]^ This can be rationalized by the stronger change in orbital character when moving from S_1_ to T_2_ and thus, following El‐Sayed's rule,^[27]^ the SOCMEs between S_1_ and T_2_ are much larger than those for S_1_→T_1_. The bromine substitution increases the SOCMEs in general with respect to those of the unsubstituted compound. As may be expected, the heavy‐atom effect is strongest for the *ortho*‐substituted compound. Even in this case, the change in excitation character is larger for the S_1_^M^→T_2_^M^ transition. In particular, the p orbital hole at the Br atom changes orientation (see above) as required for an El‐Sayed allowed transition.^[27]^ Very close to the T_2_^M^ minimum, however, the T_2_^M^ and T_1_^M^ states of ***o***‐**BrTAB** undergo a conical intersection without the necessity to surmount a substantial energy barrier. Therefore, the population is not trapped in the T_2_^M^ state but is rapidly transferred to the T_1_^M^ state. The possibility that the dual phosphorescence originates from T_2_^M^ and T_1_^M^ states can be ruled out.

As dual phosphorescence was not observed from isolated molecules in the frozen glass matrix at 77 K but only in the crystalline state and in highly doped PMMA films, we propose that this phenomenon stems from aggregated ***o***‐**BrTAB** units. To explore this effect, we optimized dimeric systems of the compounds starting from the crystal structures. Spreading the excitation over a dimer is, unfortunately, not sufficient to cause a substantial stabilization of the excitonic state. Their difference densities (Figures S16‐S18) reveal that the lowest excited dimer states are composed of excitations similar to those of the monomers. In the ***o***‐**BrTAB** dimer, the T_1_^D^ state is stabilized by only 0.05 eV with respect to the monomer. Delocalization of the excitation over larger molecular clusters is required to explain the marked redshift of the long lifetime component of the phosphorescence emission in the crystalline state.

**Figure 8:**
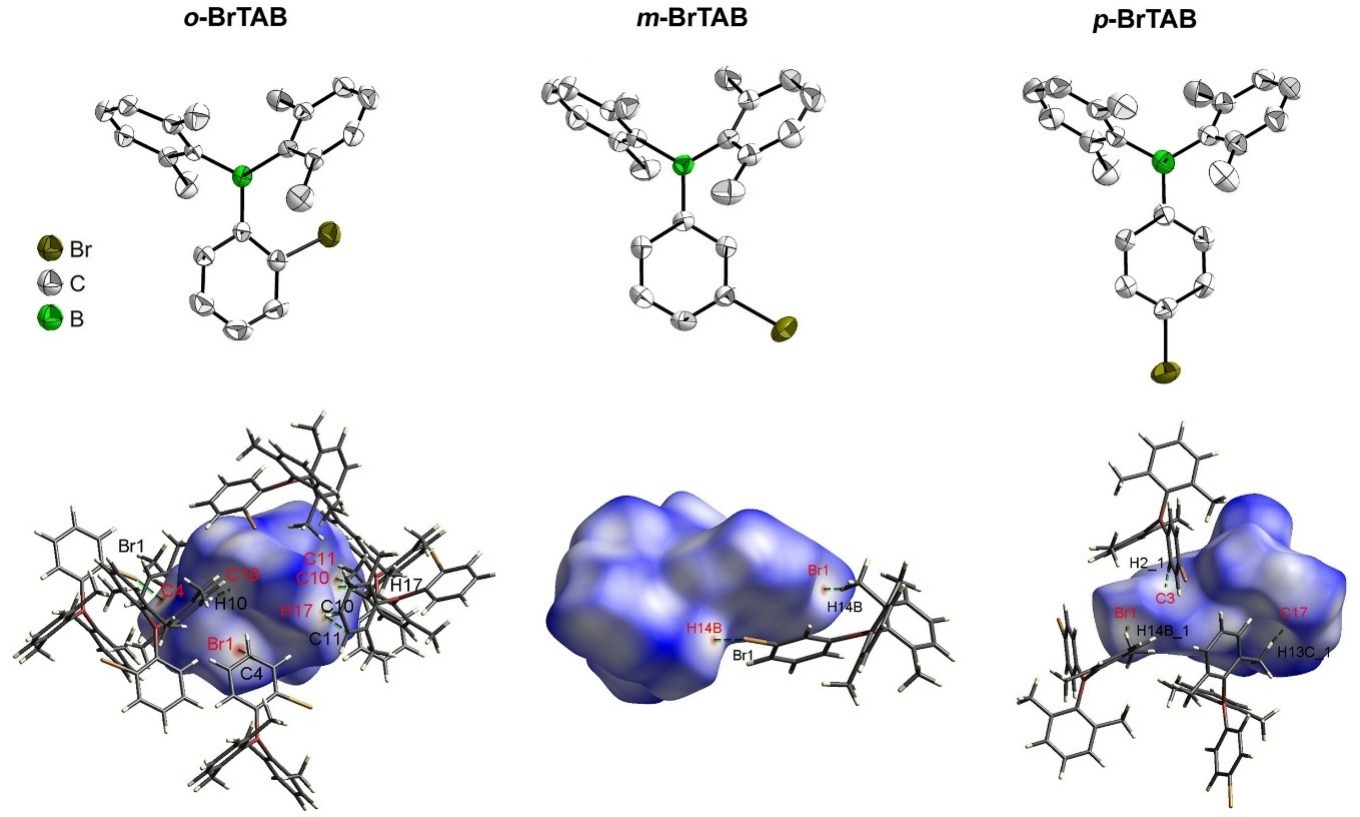
The solid‐state molecular structure of ***o***‐**BrTAB**, ***m***‐**BrTAB** and ***p***‐**BrTAB** (top left to right) determined by single‐crystal X‐ray diffraction at room temperature. Ellipsoids are drawn at the 50 % probability level, and H atoms are omitted for clarity. Hirshfeld surfaces of ***o***‐**BrTAB**, ***m*** **‐BrTAB** and ***p***‐**BrTAB** (bottom left to right) mapped with *d*_norm_ over the range −0.12 to 1.50 at room temperature. Neighboring molecules associated with close contacts are shown.

To understand the effect of the solid‐state structures and the intermolecular packing on the luminescence properties, the structures of ***o***‐, ***m***‐ and ***p***‐**BrTAB** were obtained at low temperature (100 K) and at ambient temperature (290 K for ***o***‐**BrTAB**, 296 K for ***m***‐**BrTAB**, and 300 K for ***p***‐**BrTAB**) using single‐crystal X‐ray diffraction (Figure 8). Comparison of the molecular geometries of ***o***‐, ***m***‐ and ***p***‐**BrTAB** in their crystal structures shows only a small effect of the Br atom position on the bond lengths and angles. All but one of the B−C bond distances are similar within 3 esd's (1.574(3)–1.585(2) Å at 100 K). Only the B−C bond to the Br‐substituted aryl ring in ***p***‐**BrTAB** is significantly shorter (1.563(2) and 1.561(2) Å for the two non‐symmetry equivalent molecules at 100 K, Table S11). The BC_3_ moiety is planar in all three compounds with the sum of C−B−C angles being 360° within the standard uncertainties. The individual angles are in the range 118.0(2)–122.9(2)° except for the C1−B−C7 angle in ***o***‐**BrTAB** which is significantly smaller (116.3(2)° at 100 K, Table S11). This is the angle between the Br‐substituted aryl ring R1 and the m‐xylyl group R2 arranged on the opposite side with respect to the Br−C2 bond. Another interesting feature is that the B−C1−C2 angle (127.2(2)°) to the C2 carbon atom to which the Br atom is bonded in ***o***‐**BrTAB** is significantly larger than all other B−C−C angles in ***o***‐, ***m***‐ and ***p***‐**BrTAB** which are in the range 117.5(2)–122.9(1)° at 100 K (Table S11), and the B−C1−C6 angle (117.7(2)°) to the other side of the R1 aryl ring is rather small. The larger B−C1−C2 angle in ***o***‐**BrTAB** is attributed to the bulkiness of the long Br−C bond at the *ortho* position of the R1 ring and the Br atom being close to the central B atom and the C atom of the next‐nearest *m*‐xylyl group R3 with intramolecular Br⋅⋅⋅B (3.345(2) Å) and Br⋅⋅⋅C15 (3.298(2) Å) distances below the sum of van der Waals radii (3.75 Å for Br⋅⋅⋅B and 3.53 Å for Br⋅⋅⋅C).^[28]^ The effect of the bulkiness of the substituents and, hence, repulsion between methyl groups and also the Br atom, is further observed in the torsion angles between the aryl groups and the BC_3_ planes. While the torsion angles are in a similar range (50.1–68.6°) for the *m*‐xylyl groups, a significantly smaller torsion angle (41.9°) is observed for the Br‐substituted phenyl rings. Here, the *ortho*‐ Br‐substituted phenyl ring shows a larger torsion angle (38.6(1)°) in ***o***‐**BrTAB** than the *meta*‐ and *para*‐ Br‐substituted phenyl rings (20.1‐24.8°) in ***m***‐ and ***p***‐**BrTAB** due to repulsion effects (Table S11). The molecular geometries in the solid state of ***o***‐, ***m***‐ and ***p***‐**BrTAB** at 290 K, 296 K and 300 K, respectively, are very similar to those at 100 K (Table S12).

The molecules are similarly densely packed in the three compounds as is obvious from the crystal packing coefficients (Table S16). The molecular volumes and molecular surface areas are also very similar. However, the volume of the solvent accessible voids in the unit cells, as calculated with the OLEX2 program,^[29]^ is increased in ***p***‐**BrTAB** (2.7 % at 300 K) and in ***m***‐**BrTAB** (4.4 % at 296 K) at ambient temperature, compared to ***o***‐**BrTAB** (1.7 % at 290 K) and to the low‐temperature crystal structures at 100 K (0‐1.8 % in the three compounds). This indicates a looser packing mode in ***p***‐**BrTAB** and ***m***‐**BrTAB** than in ***o***‐**BrTAB** at room temperature.

A Hirshfeld surface analysis based on the crystal structures was performed in order to quantify the nature and type of intermolecular interactions.^[30]^ The Hirshfeld surface is a special isosurface defined by the weighting function w(**r**)=0.5 for a particular molecule. This means that the Hirshfeld surface envelops the volume within which the particular molecule contributes more than half of the electron density. Hence, it also includes information on the nearest neighbors and closest contacts to the molecule (Figure 7). The similarity of the amounts and types of interactions in the three compounds is demonstrated in the two‐dimensional fingerprint plots and their breakdown to the individual relative contributions (Figures S25 and S26, 100 K).^[31]^ At 100 K, major contributions are from H⋯H interactions (61–67 %), followed by a significant amount from C⋯H (19–25 %) and Br⋯H (10–15 %) interactions. Minor contributions (0.2–1.8 %) are Br⋯C, C⋯C, and Br⋯Br interactions with C⋯C interactions being dominant in ***m***‐**BrTAB**, Br⋯Br interactions in one of the symmetry‐independent molecules (no. 2) of ***p***‐**BrTAB**, and Br⋯C interactions in both ***o***‐**BrTAB** and ***p***‐**BrTAB** (Figure S25, Table S16). Relative contributions to intermolecular interactions are very similar for the room temperature structures (Table S16).

At 100 K, the C−H⋯C interactions are strongest in ***o***‐**BrTAB**, with C⋯H distances in the range 2.735(2)‐2.812(2) Å, and two nearly linear interactions with C−H⋯C=165.82(15)° and 175.45(15)° (Table S13). C−H⋯C interactions are weaker in ***p***‐**BrTAB** (C⋯H=2.810(2)–2.865(2) Å, C−H⋯C=131.16(11)‐162.62(11)°) although of similar number as in ***o***‐**BrTAB** and, significantly, weakest in ***m***‐**BrTAB** (C⋯H=2.836(2)–2.888(3) Å, C−H⋯C=143.96(15)–166.26(15)°) as well as being fewer in number. While there are close Br⋯H contacts (2.9425(12)–3.1001(3) Å) in all three compounds, ***o***‐**BrTAB** exhibits the closest Br⋯C contact of 3.354(2) Å. Another close Br⋯C contact (3.480(2) Å) is found in ***p***‐**BrTAB**. In addition, ***o***‐**BrTAB** has a short C⋯C contact (3.361(3) Å) between an aryl ring and a methyl group, and ***p***‐**BrTAB** shows two close H⋯H contacts (2.266 and 2.363 Å) between two aryl rings (Table S16). Interestingly, ***m***‐**BrTAB** is the only compound in which two weak intermolecular π⋅⋅⋅π interactions between aryl rings can be found (Table S15). One involves the Br‐substituted phenyl rings with an interplanar separation of 3.640(3) Å and an offset shift of 2.261(4) Å. The nearest‐neighbor C⋅⋅⋅C distance is 3.698(5) Å. The other one involves the R3 xylyl rings with a slightly smaller interplanar separation of 3.544(4) Å, but a larger offset shift of 3.457(4) Å, and a nearest‐neighbor C⋅⋅⋅C distance of 3.637(5) Å.

At room temperature (290 K), there are still a significant number of intermolecular C−H⋯C interactions (C⋯H=2.846(4)–2.873(4) Å, C−H⋯C=136.4(2)–177.2(3)°) and a close Br⋯C contact of 3.432(5) Å present in ***o***‐**BrTAB** (Table S13). However, in ***m***‐**BrTAB** C⋯H contacts are long and weak, and the π⋅⋅⋅π interactions are also insignificant due to large interplanar separations of 3.812(4) Å and 3.645(6) Å with shifts of 2.305(6) Å and 3.437(6) Å, respectively, at 296 K (Table S15). Only a close Br⋯H interaction (2.9857(8) Å) with a methyl group is still significant. In ***p***‐**BrTAB**, two intermolecular C−H⋯C interactions remain (C⋯H=2.870(2) Å and 2.887(2) Å, C−H⋯C=145.18(15)° and 132.78(13)°) at 300 K, a close H⋯H interaction with 2.393(1) Å, and close Br⋯H interaction (2.9648(4) Å) with a methyl group only (Table S15). Thus, only ***o***‐**BrTAB** exhibits strong intermolecular interactions and, especially, a close Br⋅⋅⋅C contact at room temperature, which may be the reason that dual phosphorescence is only observed in ***o***‐**BrTAB** at room temperature.

In summary, the presence of multiple C−H⋯C and C−H⋯Br interactions between molecules in the crystals effectively suppresses the nonradiative decay rate *k*_nr_, which plays an important role in stabilizing the triplet states and achieving RTP. As the strength of intermolecular interactions is especially distinct between the three compounds at room temperature, and is strongest for ***o***‐**BrTAB**, this effect may be the reason that we observe DRTP in crystalline ***o***‐**BrTAB** at room temperature, but only at low temperature for ***m***‐**BrTAB** and ***p***‐**BrTAB**.

## Conclusions

We reported three bromo‐substituted triarylborane isomers which show persistent room temperature phosphorescence (RTP). Among them, (2‐bromophenyl)bis(2,6‐dimethylphenyl)borane (***o***‐**BrTAB**) exhibits rare dual room temperature phosphorescence (DRTP) with lifetimes of 0.8 ms (short wavelength component) and 234 ms (long wavelength component), respectively, in the crystalline state. Single‐crystal structure analysis shows that multiple molecular C−H⋯C and C−H⋯Br contacts in the crystals suppress the nonradiative decay rate *k*_nr_ and stabilize the triplet states. In addition, the rigid crystalline matrix provides a favorable environment for realizing dual phosphorescence at room temperature.

## Crystal structures

Deposition Number(s) 2085814 (for ***o***‐**BrTAB** at 100 K), 2085815 (for ***m***‐**BrTAB** at 100 K), 2085816 (for ***p***‐**BrTAB** at 100 K), 2089473 (for ***o***‐**BrTAB** at 290 K), 2118234 (for ***m***‐**BrTAB** at 296 K) and 2118235 (for ***p***‐**BrTAB** at 300 K) contain the supplementary crystallographic data for this paper. These data are provided free of charge by the joint Cambridge Crystallographic Data Centre and Fachinformationszentrum Karlsruhe Access Structures service.

## Conflict of interest

The authors declare no conflict of interest.

1

## Supporting information

As a service to our authors and readers, this journal provides supporting information supplied by the authors. Such materials are peer reviewed and may be re‐organized for online delivery, but are not copy‐edited or typeset. Technical support issues arising from supporting information (other than missing files) should be addressed to the authors.

Supporting Information

## Data Availability

The data that support the findings of this study are available in the supplementary material of this article.
