# Supplementary material for: Aggregation‐Induced Dual Phosphorescence from (o‐Bromophenyl)‐Bis(2,6‐Dimethylphenyl)Borane at Room Temperature
Source: Chemistry. 2022 Apr 8;28(30):e202200525. doi: 10.1002/chem.202200525 (PMC9325438; doi:10.1002/chem.202200525)
Supplement: Supplementary file 1 — Supporting Information [file CHEM-28-0-s001.pdf]

Chemistry–A European Journal

Supporting Information

Aggregation-Induced Dual Phosphorescence from (*o*-Bromophenyl)-Bis(2,6-Dimethylphenyl)Borane at Room Temperature

Zhu Wu, Fabian Dinkelbach, Florian Kerner, Alexandra Friedrich, Lei Ji, Vladimir Stepanenko, Frank Würthner, Christel M. Marian,* and Todd B. Marder*

Table of Contents

I. General information..S2

II. Experimental procedures and characterization..S5

III. SEM..S7

IV. Photophysical spectra...S8

V. Results of the quantum chemical computations...S14

VI. Single-crystal X-ray diffraction..S28

VII. ^{1}H, ^{13}C, ^{11}B NMR spectra and GC-MS and HRMS data..S42

VIII. References..S50

I. General information

All starting materials were purchased from commercial sources and were used without further purification. The organic solvents for synthetic reactions and for photophysical measurements were HPLC grade, further treated to remove trace water using an Innovative Technology Inc. Pure-Solv Solvent Purification System and deoxygenated using the freeze-pump-thaw method. All synthetic reactions were performed in an Innovative Technology Inc. glovebox or under an argon atmosphere using standard Schlenk techniques. ^{1}H, ^{13}C and ^{11}B NMR spectra were measured on a Bruker Avance 500 MHz (^{1}H, 500 MHz; ^{13}C, 126 MHz; ^{11}B, 160 MHz) NMR spectrometer. Mass spectra were recorded on Agilent 7890A/5975C Inert GC/MSD systems operating in EI mode. High resolution mass spectra were obtained using a Thermo Fisher Scientific Exactive™ Plus Orbitrap MS System with an Atmospheric Solids Analysis Probe (ASAP^{+}). Elemental analyses were performed on a Leco CHNS-932 Elemental Analyser.

General photophysical measurements. All measurements were performed in standard quartz cuvettes (1 cm × 1 cm cross-section). UV-visible absorption spectra were recorded using an Agilent 8453 diode array UV-visible spectrophotometer. The molar extinction coefficients were calculated from three independently prepared samples in hexane solution. The emission spectra were recorded using an Edinburgh Instruments FLSP920 spectrometer equipped with a double monochromator for both excitation and emission, operating in right angle geometry mode, and all spectra were fully corrected for the spectral response of the instrument. All solutions used in photophysical measurements had a concentration lower than 10^{-5} M to minimize inner filter effects during fluorescence measurements.

Quantum yield measurements. The photoluminescent quantum yields were measured using a calibrated integrating sphere (inner diameter: 150 mm) from Edinburgh Instruments combined with the FLSP920 spectrometer described above. For solution-state measurements, the longest-wavelength absorption maximum of the compound in hexane was chosen as the excitation wavelength. For solid-state measurements, the excitation wavelength was 305 nm. The phosphorescence quantum yield of compounds ***o*-**, ***m*-** and ***p*-BrTAB** were obtained using the equation:

$$\Phi_{\mathrm{P}} = \frac{B}{A} \times \Phi_{\mathrm{PL}}$$

where A and B represent the integrated area of the total photoluminescence and phosphorescence spectra, respectively. The phosphorescence component was separated from total photoluminescence (PL) based on the phosphorescence spectrum for phosphorescence quantum yields. Φ_{PL} represents the absolute photoluminescence quantum yields of the compounds in the solid state.

Lifetime measurements. Fluorescence lifetimes were recorded using the time-correlated single-photon counting (TCSPC) method on an Edinburgh Instruments FLSP920 spectrometer equipped with a high-speed photomultiplier tube positioned after a single emission monochromator. Measurements were made in right-angle geometry mode, and the emission was collected through a polarizer set to the magic angle. Solutions were excited with a pulsed diode laser at a wavelength of 316 nm at repetition rates of 5-10 MHz. The instrument response functions (IRF) were *ca.* 230 ps FWHM. The phosphorescence lifetimes were measured using a μF920 pulsed 60 W Xenon microsecond flashlamp, with a repetition rate of 0.2 or 50 Hz at room temperature and 0.1 Hz at 77 K. Decays were recorded to 10000 counts in the peak channel with a record length of at least 2000 channels. Iterative reconvolution of the IRF with a double exponential function and non-linear least-squares analysis were used to analyze the data. The quality of all decay fits was judged to be satisfactory, based on the calculated values of the reduced χ^2 and Durbin-Watson parameters and visual inspection of the weighted residuals. Time-gating was used to measure the emission following a specific delay time the range of 0.01-3 ms.

Powder X-ray diffraction and Phase analysis The compound ***o*-BrTAB** was ground into a powder using an agate mortar until hardly any room-temperature phosphorescence was observed. The powder X-ray diffraction pattern was collected in reflection geometry on a Bruker D8 Discover powder diffractometer with Da Vinci design and linear Lynx-Eye detector. X-ray radiation (Cu-Kα_1; λ = 1.5406 Å) was focused with a Goebel mirror and Cu-Kα_2 radiation was eliminated by a Ni-absorber. Data were collected from $2\theta = 2 – 60°$ in steps of 0.025° at ambient temperature. They were corrected for an offset in 2θ and exported using the Bruker AXS Diffrac-Suite. The diffraction patterns were then converted using CMPR software[1] for further processing with the GSAS program.[2] Cell parameters, background, scaling factor, zero shift and profile parameters were refined using the LeBail method. The data range $2\theta = 2 – 5°$ was excluded from refinement as no reflection was either observed or predicted in this range and due to high background signal at low angles. The starting values for the refinement were taken from the single-crystal structure refinement at 100 K.

Scanning Electron Microscopy SEM images were recorded using a Zeiss Ultra Plus field emission scanning electron microscope equipped with GEMINI e-Beam column operated at 1-3 kV with an aperture size set to 10 or 30 μm to avoid excessive charging and radiation damage of the areas imaged.

Quantum chemical calculations All geometries were obtained using the Turbomole[3] and Gaussian[4] program packages utilizing Kohn–Sham density functional theory (DFT) in combination with the PBE0[5-7] functional. For excited state optimizations, linear response time-dependent DFT (TDDFT) was employed, and, for triplet states, the Tamm–Dancoff approximation (TDDFT-TDA) was additionally used. Vibrational frequency calculations were carried out with the Gaussian program package. In all computations, the def2-SVP[8] basis set was utilized on all atoms except for bromine, for which a cc-pVTZ-PP[9] basis set in combination with the defpp-ecp[9] pseudopotential was employed. Energies and multi reference configuration interaction (MRCI) wavefunctions of the lowest ten excited states of singlet and triplet spin multiplicity, respectively, were optimized with the DFT/MRCI[10-11] method applying the redesigned R2016 Hamiltonian[12] and an initial active space of 12 electrons in 12 frontier orbitals. The orbital basis for the DFT/MRCI calculations is obtained from DFT utilizing the BH-LYP[13-14] functional. Absorption spectra were obtained by broadening the corresponding DFT/MRCI line spectra with Gaussian functions of 1000 cm^{-1} full width at half maximum (FWHM). The SPOCK[15-17] program package was employed to compute spin–orbit coupling matrix elements (SOCMEs) and phosphorescence rate constants from spin–orbit coupled wavefunctions obtained with the MRSOCI procedure. Franck–Condon (FC) emission spectra and intersystem crossing (ISC) rate constants were obtained utilizing a time evolution approach implemented in the VIBES[18-19] program. Here an integration grid of 16384 points over 300 fs time period and a Gaussian damping function of 200 cm^{-1} FWHM was employed for FC spectra. For ISC rate constants, a grid of 1000 points over a 250 fs time period and a Gaussian damping function of 0.3 cm^{-1} FWHM was used. Temperature effects of the population of the vibrational modes were accounted for by a Boltzmann distribution in the initial electronic state.

Crystal structure determinations Crystals suitable for single-crystal X-ray diffraction were selected, coated in perfluoropolyether oil, and mounted on MiTeGen sample holders. Diffraction data were collected on Bruker X8 Apex II 4-circle diffractometers with CCD area detectors using Mo-K_α radiation monochromated by graphite or multi-layer focusing mirrors or on a Bruker D8-Quest diffractometer with a CPA area detector and multi-layer mirror monochromated Mo-K_α radiation. Data were collected at ambient temperature and at 100 K.

The crystals were cooled using an Oxford Cryostream low-temperature device. The images were processed and corrected for Lorentz-polarization effects and absorption as implemented in the Bruker software packages. The structures were solved using the intrinsic phasing method (SHELXT)[20] and Fourier expansion technique. All non-hydrogen atoms were refined in anisotropic approximation, with hydrogen atoms 'riding' in idealized positions, by full-matrix least squares against F^2 of all data, using SHELXL[21] software and the SHELXLE graphical user interface.[22] Diamond[23] software was used for graphical representation. Other structural information was extracted using Mercury[24] and OLEX2[25] software. Hirshfeld surfaces were calculated and analyzed using the Crystal Explorer[26] program. Crystal data and experimental details are listed in Tables S9 and S10; full structural information has been deposited with Cambridge Crystallographic Data Centre. CCDC-2085814 (***o*-BrTAB** at 100 K), CCDC-2085815 (***m*-BrTAB** at 100 K), CCDC-2085816 (***p*-BrTAB** at 100 K), CCDC-2089473 (***o*-BrTAB** at 290 K), CCDC-2118234 (***m*-BrTAB** at 296 K) and CCDC-2118235 (***p*-BrTAB** at 300 K).

II. Experimental procedures and characterization

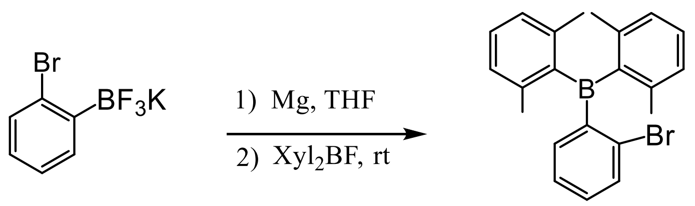

(2-bromophenyl)bis(2,6-dimethylphenyl)borane (*o*-BrTAB): A 250 mL three-necked round bottom flask, equipped with a dropping funnel and water-cooled condenser, was used. In a typical preparation, an anhydrous THF solution of 2-bromo-1,3-dimethylbenzene (896 mg, 4.5 mmol) was added dropwise to the flask containing magnesium (120 mg, 5.0 mmol) and 100 mL of anhydrous THF. The solution was refluxed during the addition period, which required approximately 3 h, and then allowed to cool to room temperature. To this solution (2-bromophenyl)trifluoroborate potassium salt[27] (789 mg, 3.0 mmol) in anhydrous THF was added and the reaction was stirred overnight. The resulting mixture was subjected to silica gel column chromatography eluting with *n*-hexane to afford ***o*-BrTAB** (722 mg,1.92 mmol) in 64% yield as white a solid: ^{1}H NMR (500 MHz, CD_2Cl_2, rt, ppm): δ 7.58–7.55 (m, 1H), 7.30–7.27 (m, 2H), 7.20–7.16 (m, 3H), 6.95 (d, *J* = 8 Hz, 4H), δ 2.03 (s, 12H). ^{13}C NMR (126 MHz, CD_2Cl_2, rt, ppm): 149.5 (br), 145.5 (br), 141.0, 136.1, 133.1, 132.3, 130.0, 128.4, 127.8, 127.4, 23.2. ^{11}B NMR (160 MHz, CD_2Cl_2, r.t., ppm): 76 (br). MS (EI^+) m/z: 376

$[M]^+$. HRMS ($ASAP^+$): m/z calcd for 376.0992 $[M]^+$; found: 376.0990 [*M*] (|Δ| = 0.53 ppm). Elem. Anal. Calcd (%) for $C_{22}H_{22}BBr$: C, 70.07; H, 5.88; Found: C, 69.91; H, 6.07.

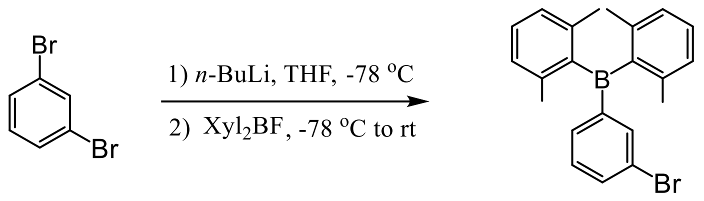

(3-bromophenyl)bis(2,6-dimethylphenyl)borane (*m*-BrTAB): To a solution of 1,3-dibromobenzene (402 mg, 1.7 mmol) in anhydrous THF (20 mL) was added a hexane solution of *n*-BuLi (1.2 mL, 1.6 M, 1.9 mmol) dropwise by syringe at -78 °C. The mixture was stirred at the same temperature for 1 h. A solution of bis(2,6-dimethylphenyl)fluoroborane[28] (408 mg, 1.7 mmol) in anhydrous THF (5 mL) was added to the reaction mixture via syringe. The reaction mixture was warmed to room temperature and stirred overnight. The reaction was quenched with a saturated solution of NaCl and the aqueous layer was extracted with Et_2O. The combined organic layer was dried over anhydrous Na_2SO_4, filtered, and concentrated under reduced pressure. The resulting mixture was subjected to silica gel column chromatography eluting with *n*-hexane to afford ***m*-BrTAB (**498 mg, 1.3 mmol) in 78% yield as a white solid: ^{1}H NMR (500 MHz, CD_2Cl_2, rt, ppm): δ 7.68 (ddd, J = 8, 2 and 1 Hz, 1H), 7.65–7.63 (m, 1H), 7.46 (dt, J = 7, 1 Hz, 1H), 7.35–7.28 (m, 1H), 7.25 (t, J = 8 Hz, 2H), 7.08–7.02 (m, 4H), 2.07 (s, 12H). $^{13}C\{^1H\}$ NMR (126 MHz, CD_2Cl_2, rt, ppm): 148.1 (br), 143.9 (br), 140.6, 138.3, 134.8, 134.6, 130.0, 129.4, 127.4, 122.9, 23.3. ^{11}B NMR (160 MHz, CD_2Cl_2, rt, ppm): 75 (br). MS (EI^+) m/z: 376 $[M]^+$. HRMS ($ASAP^+$): m/z calcd for $[M]^+$: 376.0930; found: 376.0937[*M*] (|Δ| = 1.86 ppm). Elem. Anal. Calcd (%) for $C_{22}H_{22}BBr$: C, 70.07; H, 5.88; Found: C, 69.98; H, 6.07.

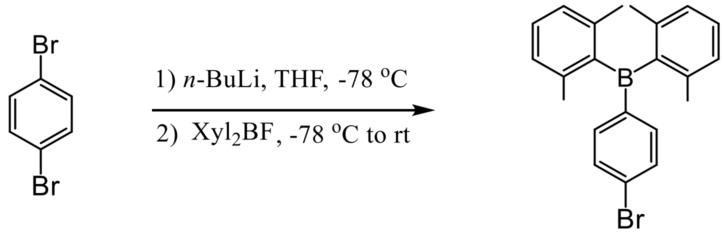

(4-bromophenyl)bis(2,6-dimethylphenyl)borane (*p*-BrTAB): To a solution of 1,4-dibromobenzene (402 mg, 1.7 mmol) in anhydrous THF (20 mL) was added a hexane solution of *n*-BuLi (1.2 mL, 1.6 M, 1.9 mmol) dropwise by syringe at -78 °C. The mixture was stirred at the same temperature for 1 h. A solution of bis(2,6-dimethylphenyl)fluoroborane[28] (408 mg, 1.7 mmol) in anhydrous THF (5 mL) was added to the reaction mixture via syringe. The reaction mixture was warmed to room temperature and stirred overnight. The reaction

was quenched with a saturated solution of NaCl and the aqueous layer was extracted with Et_2O. The combined organic layer was dried over anhydrous Na_2SO_4, filtered, and concentrated under reduced pressure. The resulting mixture was subjected to silica gel column chromatography eluting with *n*-hexane to afford ***p*-BrTAB** **(**537 mg, 1.4 mmol) in 84% yield as a white solid: ^{1}H NMR (500 MHz, CD_2Cl_2, rt, ppm): δ 7.55–7.51 (m, 2H), 7.39–7.35 (m, 2H), 7.20 (t, J = 8 Hz, 2H), 7.03–6.98 (m, 4H), 2.03 (s, 12H). ^{13}C{^{1}H} NMR (126 MHz, $CDCl_3$, rt, ppm): 144.2 (br), 144.0 (br.), 140.8, 138.1, 131.6, 129.4, 127.9, 127.5, 23.7. ^{11}B NMR (160 MHz, CD_2Cl_2, rt, ppm): 74 (br). MS (EI^+) m/z: 376 $[M]^+$. HRMS ($ASAP^+$): m/z calcd for $[M]^+$: 376.0992; found: 376.0993 [*M*] (|Δ| = 0.27 ppm). Elem. Anal. Calcd (%) for $C_{22}H_{22}BBr$: C, 70.07; H, 5.88; Found: C, 70.04; H, 6.02.

III. SEM

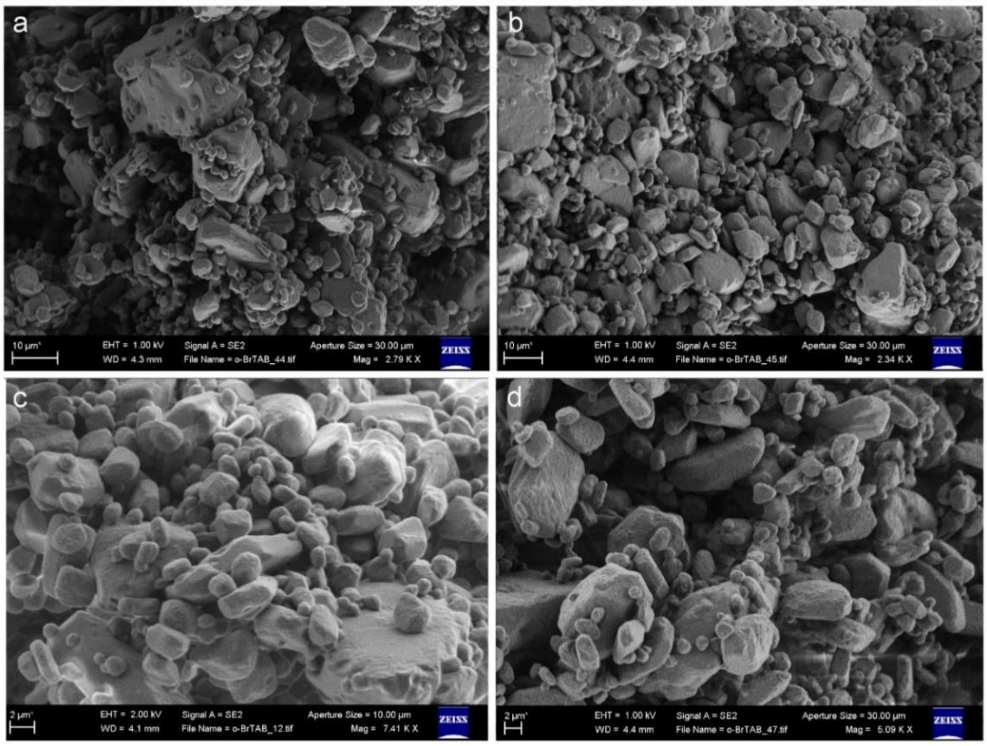

Figure S1. (a)-(d) SEM pictures of ***o*-BrTAB** as a ground powder.

IV. Photophysical spectra

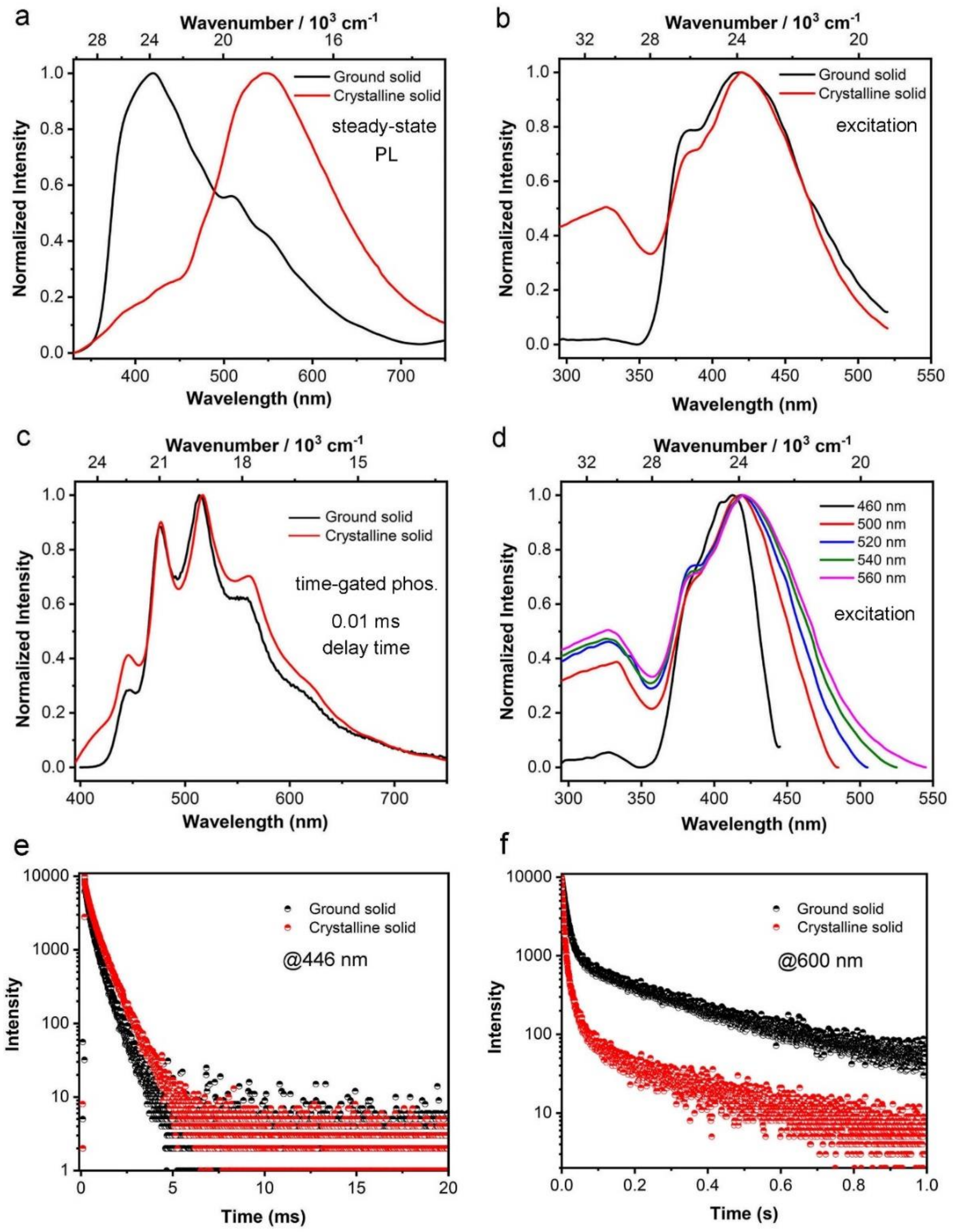

Figure S2. Normalized (a) photoluminescence emission, (b) excitation, and (c) time-gated (delay time 0.01 ms) phosphorescence emission of ***o*-BrTAB** in the crystalline state and ground powder at room temperature in air (λ_{ex} = 305 nm). (d) Normalized wavelength-dependent excitation spectra of ***o*-BrTAB** in the crystalline state from λ_{ex} = 460 to 560 nm. (e) Phosphorescence decay (λ_{em} = 446 nm) of ***o*-BrTAB** in the crystalline state (τ_p= 0.8 ms) and ground powder (τ_p = 0.7 ms) at room temperature in air. (f) Phosphorescence decay (λ_{em} = 600 nm) of ***o*-BrTAB** in the crystalline state (τ_p = 234 ms) and ground powder (τ_p = 191 ms) at room temperature in air.

Table S1. Unit cell parameters of ***o*-BrTAB** obtained from the LeBail refinement of powder X-ray diffraction data at room temperature.

**Data**	***o*-BrTAB**
λ / Å, radiation	1.5406, Cu-Kα_1
θ range / °	5 – 60
a / Å	8.2022(5)
b / Å	12.1584(5)
c / Å	18.8797(6)
α / °	90.0
β / °	93.312(4)
γ / °	90.0
Volume / Å^3	1879.7(1)
R_p	0.0495
wR_p	0.0798

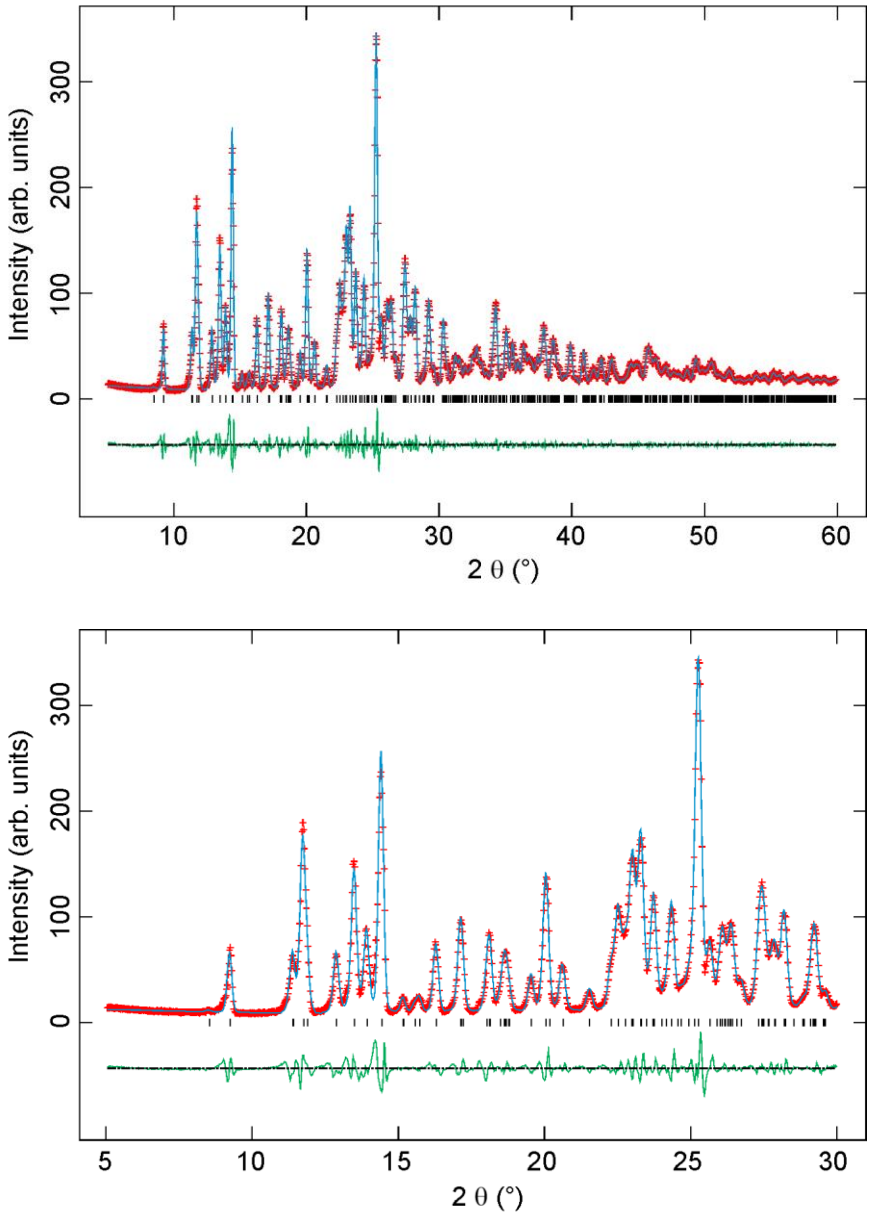

Figure S3. Powder X-ray diffraction patterns of compound ***o*-BrTAB** after grinding crystals in an agate mortar (top) in the full range 5 – 60° and (bottom) in the selected range 2θ = 5 – 30°. Red crosses represent the experimental values. The continuous blue lines show the results of the Le Bail fit to the data. The difference between experimental data and LeBail fit is represented by the green lines at the bottom of the plots. Vertical bars show the positions of the Bragg reflections of ***o*-BrTAB**.

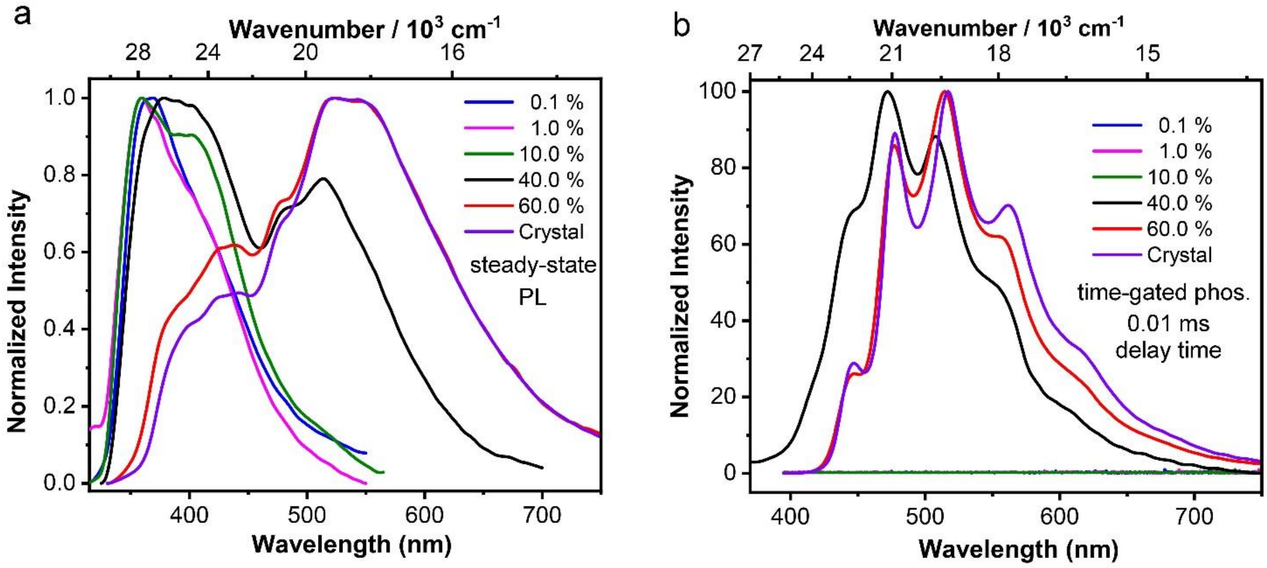

Figure S4. Normalized (a) PL emission and (b) time-gated phosphorescence emission spectra (delay time 0.01 ms) spectra of ***o*-BrTAB** in 0.1, 1.0, 10, 40 and 60 % PMMA film and the crystalline state (λ_{ex} = 305 nm).

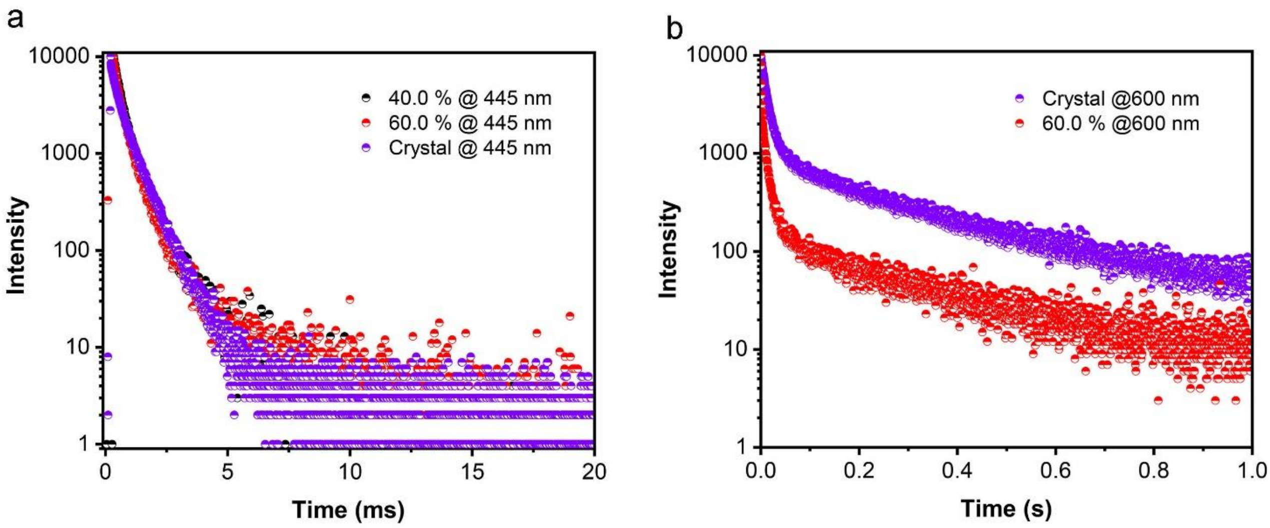

Figure S5. (a) Decays of the phosphorescence emission (λ_{em} = 446 nm, τ_p = 0.8 ms) of ***o*-BrTAB** in 40 % PMMA film, 60 % PMMA film and the crystalline state at room temperature in air. (b) Decays of the phosphorescence emission at 600 nm of ***o*-BrTAB** in 60 % PMMA film (τ_p = 226 ms) and the crystalline state (τ_p = 234 ms) at room temperature in air.

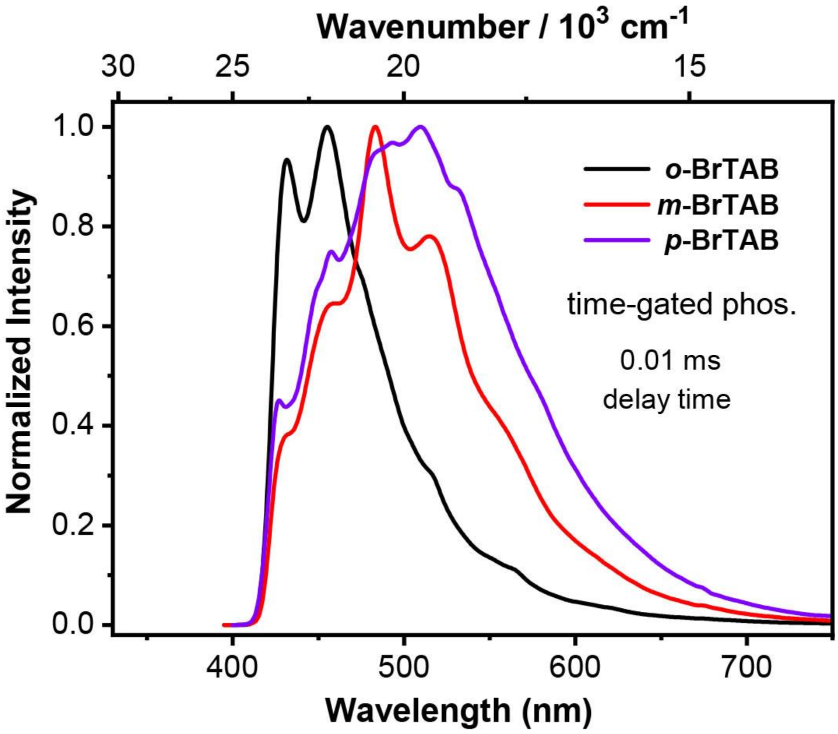

Figure S6. Normalized time-gated (delay time 0.01 ms) phosphorescence emission spectra of crystalline ***o*-**, ***m*-** and ***p*-BrTAB** at 77 K (λ_{ex} = 305 nm).

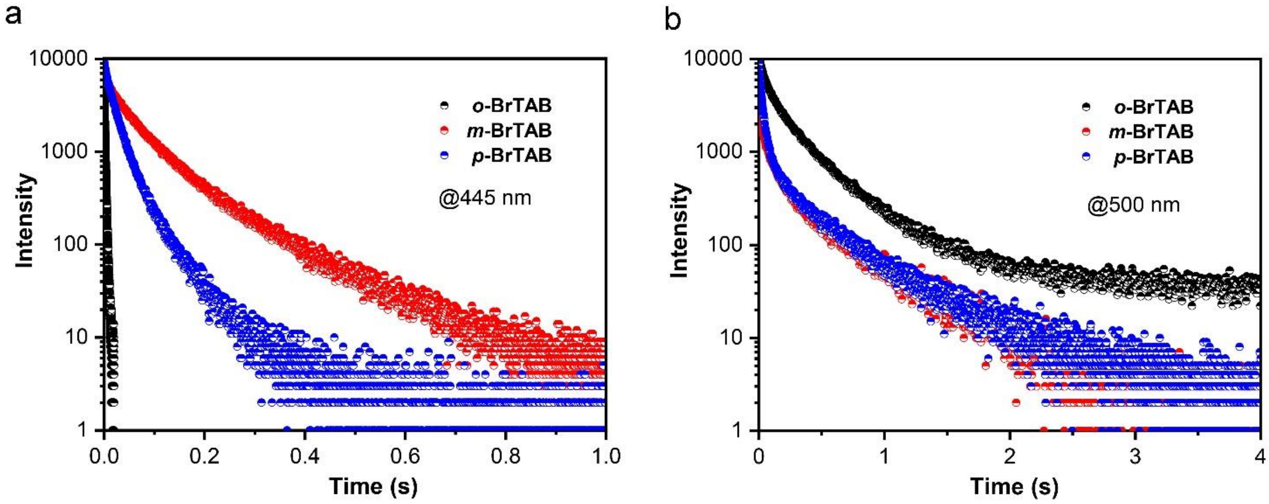

Figure S7. Decays of the phosphorescence emission (a) (λ_{em} = 445 nm) and (b) (λ_{em} = 500 nm) of crystalline ***o*-**, ***m*-** and ***p*-BrTAB** at 77 K.

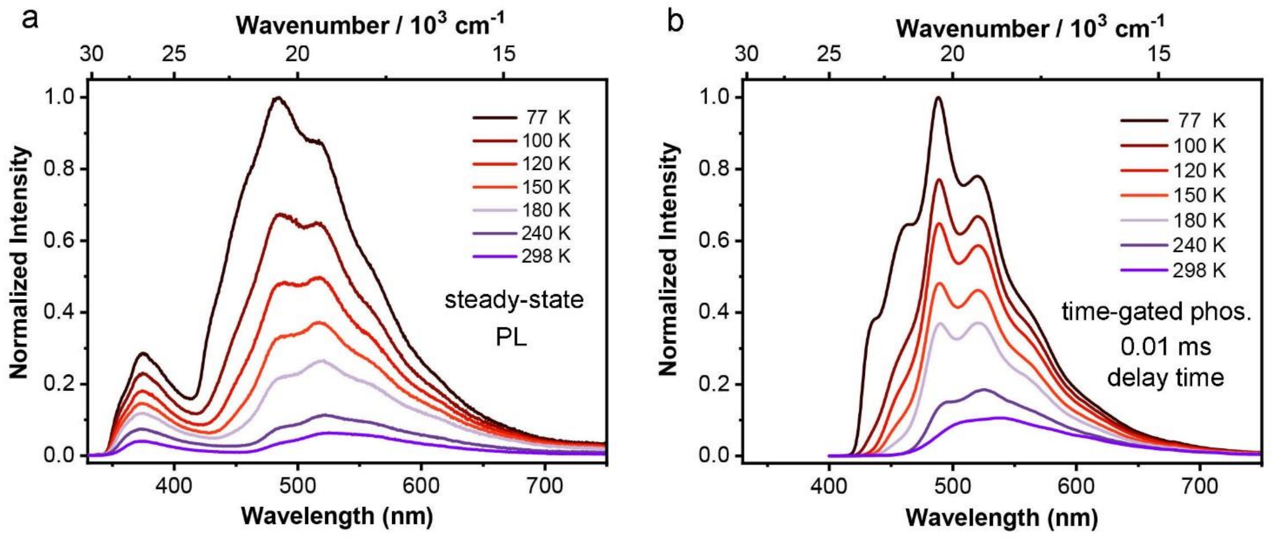

Figure S8. (a) Photoluminescence emission and (b) time-gated (delay time 0.01 ms) phosphorescence emission spectra of crystalline ***m*-BrTAB** at different temperatures (λ_{ex} = 305 nm).

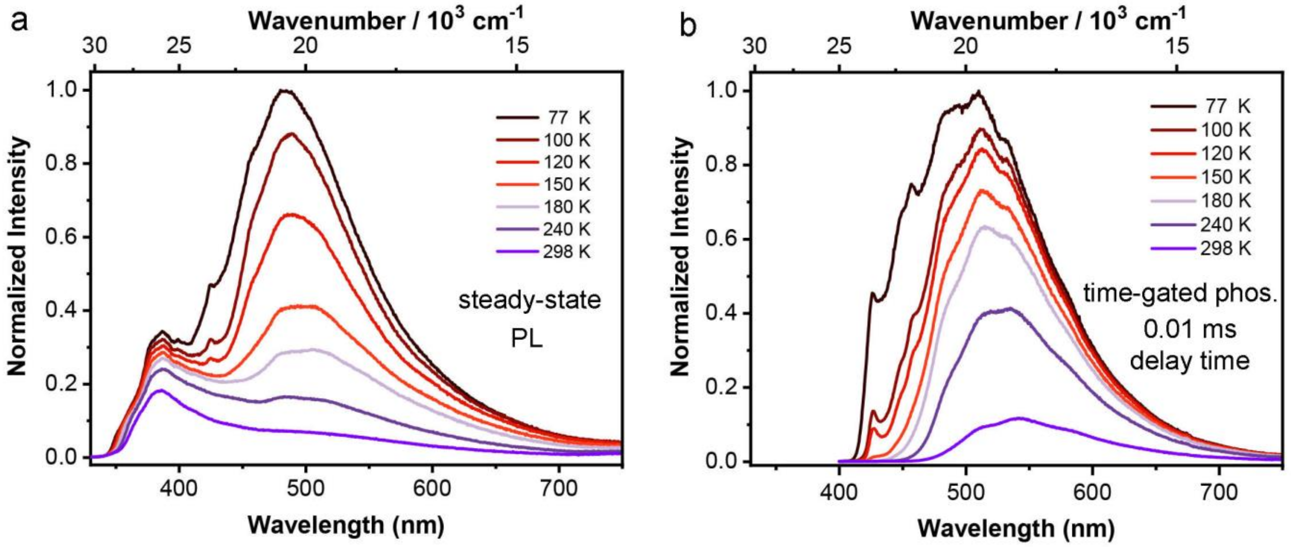

Figure S9. (a) Photoluminescence emission and (b) time-gated (delay time 0.01 ms) phosphorescence emission spectra of crystalline ***p*-BrTAB** at different temperatures (λ_{ex} = 305 nm).

V. Results of the quantum chemical computations

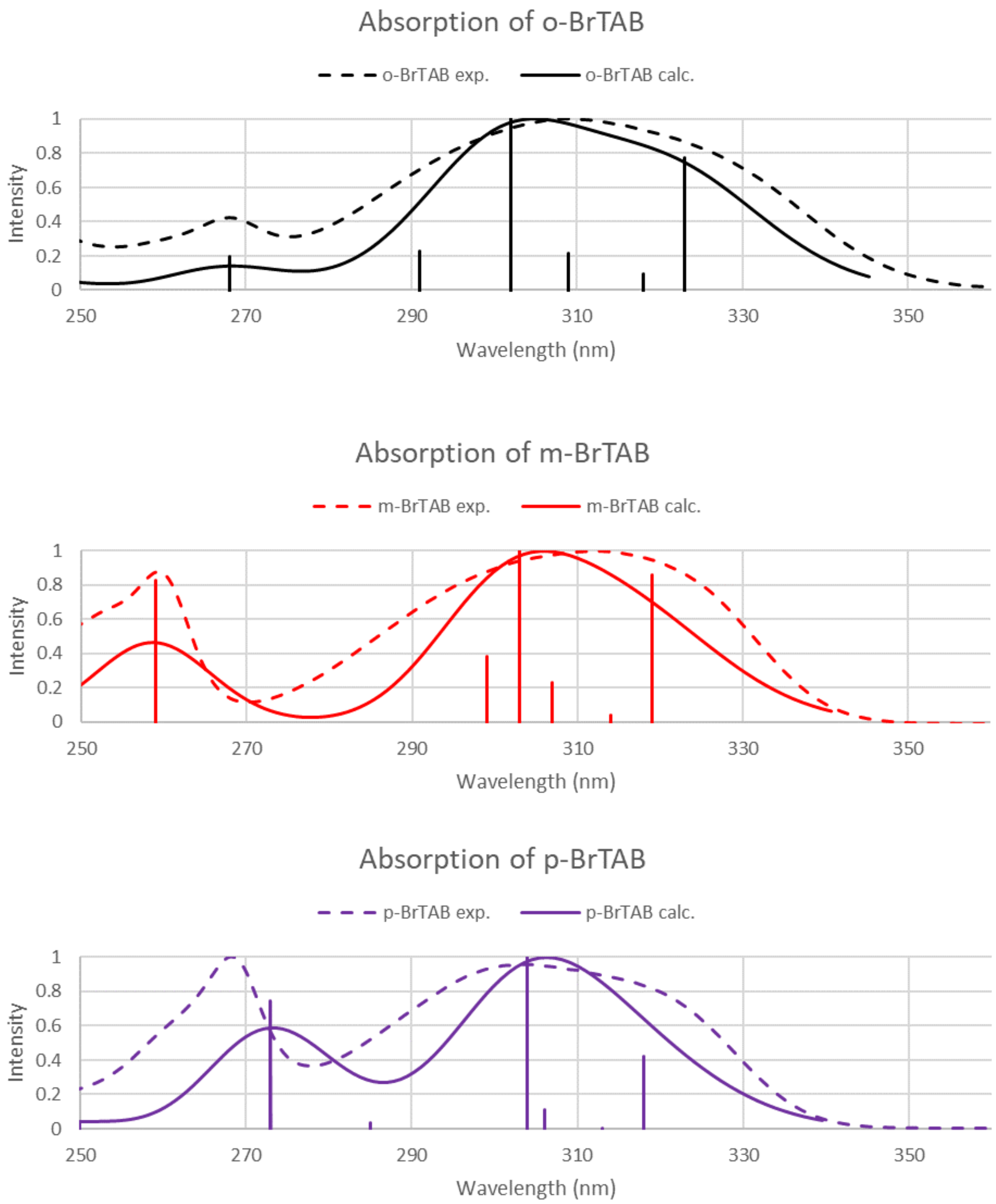

Figure S10. Calculated absorption spectra of ***o*-BrTAB**, ***m*-BrTAB** and ***p*-BrTAB** compared to experimental spectra.

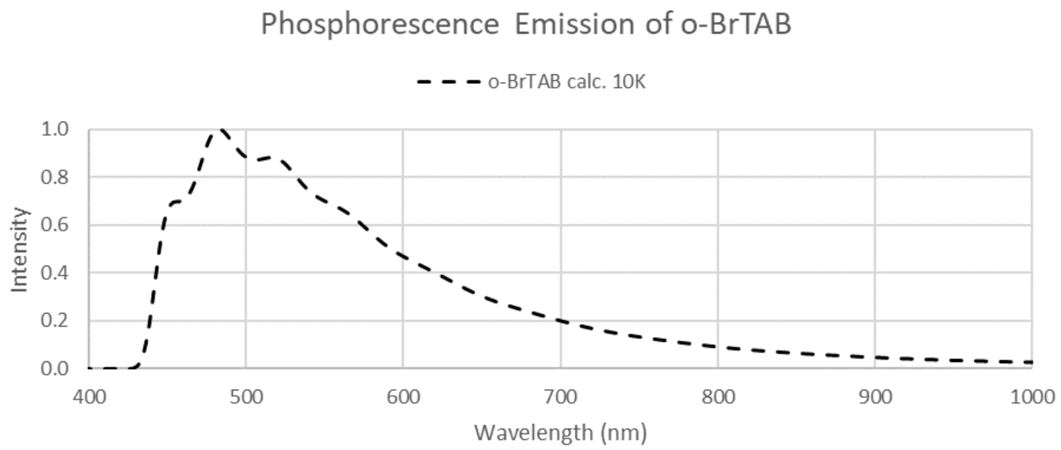

Figure S11. Calculated phosphorescence spectrum of monomeric ***o*-BrTAB** at 10 K.

Table S2: Spin–orbit coupling matrix elements (absolute values, cm^{-1}) of S_1^M, T_1^M, T_2^M and T_3^M states at the respective S_1^M minimum.

**SOCME**	**x**	**y**	**z**	**$(x^2 + y^2 + z^2)$**
		***o*-BrTAB**		
$\langle T_1^M \vert \hat{H}_{SO} \vert S_1^M \rangle$	4.792	1.299	0.833	25.348
$\langle T_2^M \vert \hat{H}_{SO} \vert S_1^M \rangle$	122.773	44.841	39.445	18639.878*
		***m*-BrTAB**		
$\langle T_1^M \vert \hat{H}_{SO} \vert S_1^M \rangle$	0.117	0.165	0.106	0.052
$\langle T_2^M \vert \hat{H}_{SO} \vert S_1^M \rangle$	7.238	0.901	2.988	62.133
$\langle T_3^M \vert \hat{H}_{SO} \vert S_1^M \rangle$	9.894	3.037	3.934	122.585*
		***p*-BrTAB**		
$\langle T_1^M \vert \hat{H}_{SO} \vert S_1^M \rangle$	0.263	0.140	2.740	7.594*
$\langle T_2^M \vert \hat{H}_{SO} \vert S_1^M \rangle$	0.390	0.055	0.374	0.295
$\langle T_3^M \vert \hat{H}_{SO} \vert S_1^M \rangle$	0.353	1.055	1.805	4.497*

* marks the fastest ISC channel of the low lying singlet and triplet states.

Table S3. Photophysical properties of compounds ***o*-BrTAB**, ***m*-BrTAB** and ***p*-BrTAB** *in vacuo* as obtained from quantum chemical calculations.

	**λ_{abs} nm [a]**	**k_{isc} s^{-1} [b]**	**λ_f nm [c]**	**k_f s^{-1}**	**τ_f ns [d]**	**λ_p nm [c]**	**k_p s^{-1}**	**τ_p s [d]**
***o*-BrTAB**	305	$\approx 1 \cdot 10^{10}$	500	$1 \cdot 10^7$	81	451, 488, 515, 563	515	0.002
***o*-Dimer**				$1 \cdot 10^7$	96	-	1041	0.001
***m*-BrTAB**	306	$\approx 9 \cdot 10^8$	412	$2 \cdot 10^7$	49	-	0.202	4.94
***p*-BrTAB**	306	$\approx 3 \cdot 10^8$	385	$2 \cdot 10^7$	45	-	1.03	0.97

[a]Absorption maximum of line spectrum broadened by Gaussian function; [b]rate constant for the fastest of the open ISC channels (***o*-BrTAB**: $S_1^M \rightarrow T_2^M$, ***m*-BrTAB**: $S_1^M \rightarrow T_3^M$ and ***p*-BrTAB**: $S_1^M \rightarrow T_1^M/T_3^M$); [c] maximum of 0-0 band, maximum of Franck-Condon spectrum at 10 K; [d] radiative lifetime assuming a quantum yield of 100 %.

Table S4. Calculated phosphorescence lifetimes of the lowest triplet components.

τ_p [s]	T_α	T_β	T_γ	$T_{average}$	$T_{exp, short}$ [a]	$T_{exp, long}$ [a]
***o*-BrTAB**	0.0007	0.0367	0.0070	0.0019	0.8077	174.2307
***o*-Dimer**	0.0003	0.0200	0.0297	0.0010		
***m*-BrTAB**	2.3030	8.4451	18.3713	4.2161	5.6667	56.8889
***m*-Dimer**	3.6310	0.3058	4.1039	0.7918		
***p*-BrTAB**	1.6762	17.2833	0.4107	0.9712	0.9000	29.0500
***p*-Dimer**	0.2339	3.5239	0.6741	0.4965		

[a] Radiative phosphorescence lifetime (τ_p/Φ_p) obtained from the experimental lifetime (τ_p) measured in the crystal at RT and the phosphorescence quantum yield (Φ_p).

Table S5. Vertical and adiabatic energies of the low-lying excited singlet and triplet states in the monomers. Adiabatic energies are marked in grey.

Energies [eV]	@S_0^M	@S_1^M	@T_1^M	@T_2^M	@T_3^M
			***o*-BrTAB**		
S_1^M	3.85	3.56	3.64	3.86[a]	
T_1^M	3.26	3.25	2.98	3.17[a]	
T_2^M	3.39	3.80	3.66	3.26[a]	
T_3^M	3.53	3.98	3.79	3.63[a]	
			***m*-BrTAB**		
S_1^M	3.89	3.78	3.96	3.94	3.91
T_1^M	3.32	3.39	3.03	3.83	3.27
T_2^M	3.38	3.52	3.86	3.01	3.38
T_3^M	3.49	3.71	3.95	3.94	3.42
			***p*-BrTAB**		
S_1^M	3.91	3.82	3.99	3.99	3.90
T_1^M	3.32	3.48	2.99	3.93	3.15
T_2^M	3.32	3.41	3.51	3.04	3.29
T_3^M	3.43	4.71	3.61	3.88	3.34

[a] Obtained at the crossing point between T_1 and T_2.

Table S6. Adiabatic energy gaps including vibrational zero-point energies.

Energies [eV]	*o*-BrTAB	*m*-BrTAB	*p*-BrTAB
$S_1^M - S_0^M$	3.48	3.66	3.71
$T_1^M - S_0^M$	2.89	2.91	2.93
$S_1^M - T_1^M$	0.60	0.75	0.83
$S_1^M - T_2^M$		0.76	0.78
$S_1^M - T_3^M$		0.26	0.41

Table S7. Vertical and adiabatic energies of the low-lying excited singlet and triplet states in the dimers. Adiabatic energies are marked in grey.

**Energies [eV]**	**@S_0^D**	**@S_1^D**	**@T_1^D**
		***o*-BrTAB**	
S_1^D	3.65	3.41	3.51
T_1^D	3.07	3.11	2.93
T_2^D	3.07	3.61	3.52
T_3^D	3.21	3.78	3.54
T_4^D	3.21	3.83	3.71
		***m*-BrTAB**	
S_1^D	3.75	3.64	3.68
T_1^D	3.26	3.33	2.93
T_2^D	3.26	3.54	3.63
T_3^D	3.30	3.80	3.72
T_4^D	3.30	3.81	3.79
		***p*-BrTAB**	
S_1^D	3.79	3.65	3.81
T_1^D	3.20	3.23	3.02
T_2^D	3.24	3.41	3.48
T_3^D	3.25	3.66	3.63
T_4^D	3.37	3.71	3.64

Table S8: Selected bond lengths and angles of the isolated compounds ***o*-BrTAB**, ***m*-BrTAB** and ***p*-BrTAB** as obtained from geometry optimizations of the S_0^M, S_1^M, T_1^M, T_2^M and T_3^M states. R1 = bromo-xylyl, R2 = R3 = xylyl

***o*-BrTAB**	**@S₀**^{M}	**@S₁**^{M}	**@T₁**^{M}		
B-R1	1.581	1.532	1.566		
B-R2	1.578	1.614	1.560		
B-R3	1.582	1.584	1.587		
C-Br	1.896	1.912	1.902		
∠ R1(C)-B-(C-C)R2	49.4	67.2	49.2		
∠ R2(C)-B-(C-C)R3	51.7	47.4	49.7		
∠ R3(C)-B-(C-C)R1	42.4	23.1	31.3		
∠ R2(C)-B-(C-C)R1	44.2	19.3	33.3		
∠ R3(C)-B-(C-C)R2	52.3	58.1	46.3		
∠ R1(C)-B-(C-C)R3	55.3	49.8	51.1		
***m*-BrTAB**	**@S₀**^{M}	**@S₁**^{M}	**@T₁**^{M}	**@T₂**^{M}	**@T₃**^{M}
B-R1	1.574	1.531	1.568	1.568	1.553
B-R2	1.581	1.586	1.594	1.560	1.589
B-R3	1.582	1.606	1.559	1.595	1.587
C-Br	1.891	1.900	1.893	1.894	1.873
∠ R1(C)-B-(C-C)R2	54.5	48.4	62.5	36.1	-51.9
∠ R2(C)-B-(C-C)R3	55.0	65.6	42.1	61.7	-55.7
∠ R3(C)-B-(C-C)R1	27.8	13.2	25.6	20.1	-20.1
∠ R2(C)-B-(C-C)R1	27.9	19.6	20.3	25.3	-22.2
∠ R3(C)-B-(C-C)R2	54.6	49.1	61.6	41.9	-51.2
∠ R1(C)-B-(C-C)R3	54.9	69.8	36.4	62.7	-55.7
***p*-BrTAB**	**@S₀**^{M}	**@S₁**^{M}	**@T₁**^{M}	**@T₂**^{M}	**@T₃**^{M}
B-R1	1.571	1.530	1.543	1.566	1.561
B-R2	1.583	1.606	1.588	1.559	1.584
B-R3	1.583	1.586	1.588	1.595	1.584
C-Br	1.887	1.890	1.854	1.887	1.872
∠ R1(C)-B-(C-C)R2	55.1	69.5	52.4	35.2	53.4
∠ R2(C)-B-(C-C)R3	55.0	49.1	52.3	63.5	53.1
∠ R3(C)-B-(C-C)R1	27.2	19.8	21.8	19.2	26.4
∠ R2(C)-B-(C-C)R1	27.2	13.2	21.8	24.9	26.4
∠ R3(C)-B-(C-C)R2	55.0	65.5	51.6	41.0	53.1
∠ R1(C)-B-(C-C)R3	55.0	48.4	52.9	63.9	53.4

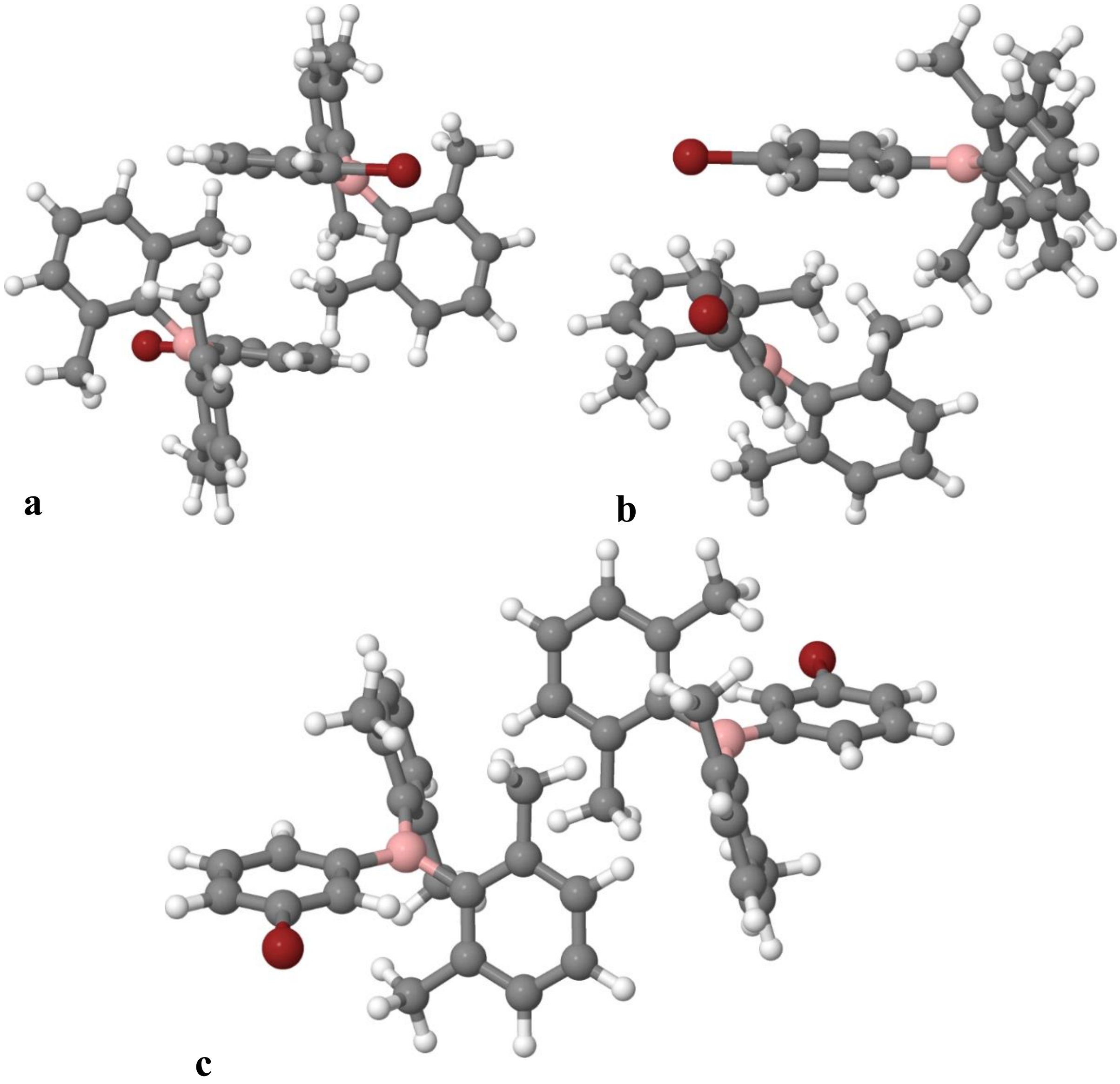

Figure S12. Dimer geometries of ***o*-BrTAB** (a), ***p*-BrTAB** (b) and ***m*-BrTAB** (c).

Difference Densities:

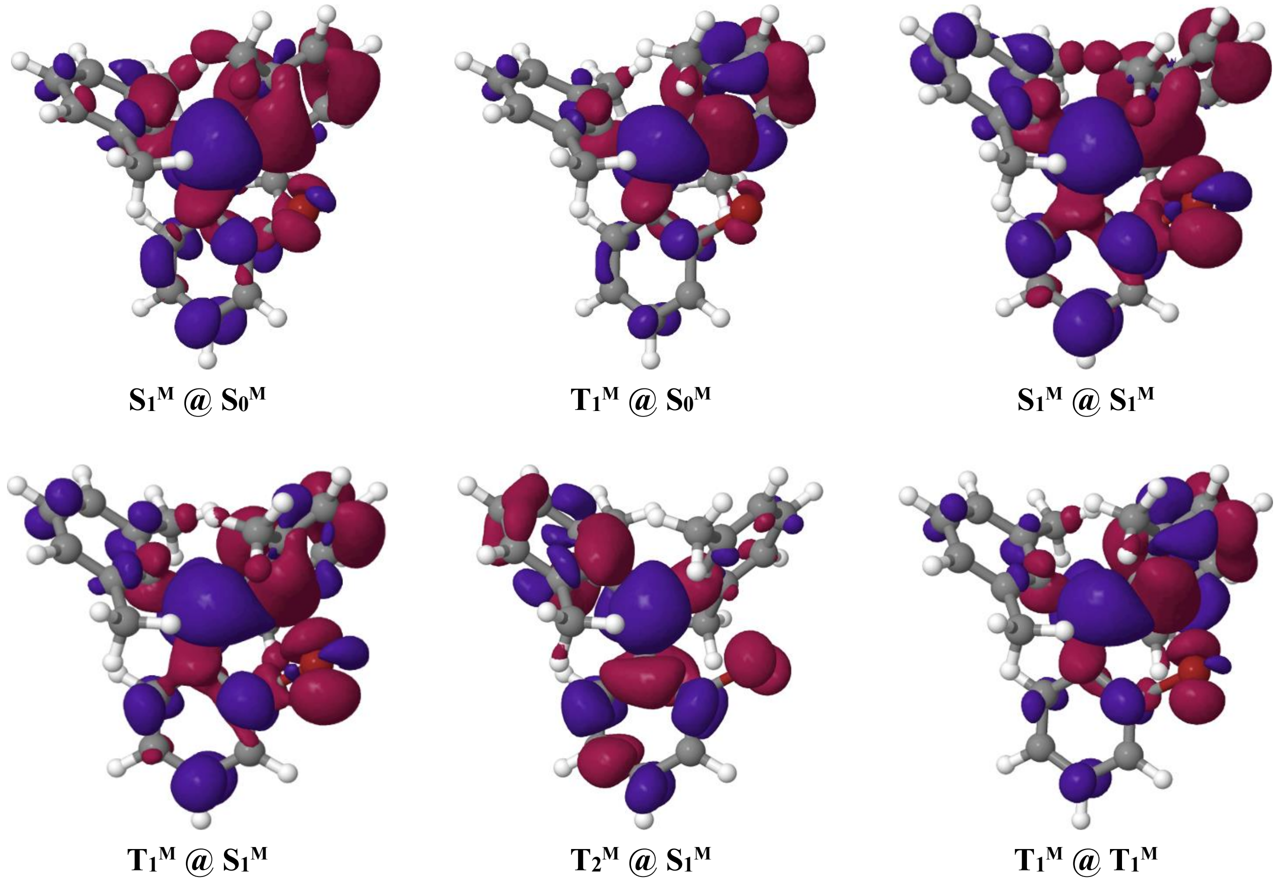

Figure S13. Difference densities of the S_1^M, T_1^M, and T_2^M states of ***o*-BrTAB** at various molecular geometries. Red areas indicate a loss of electron density upon excitation from the electronic ground state, and blue areas a gain.

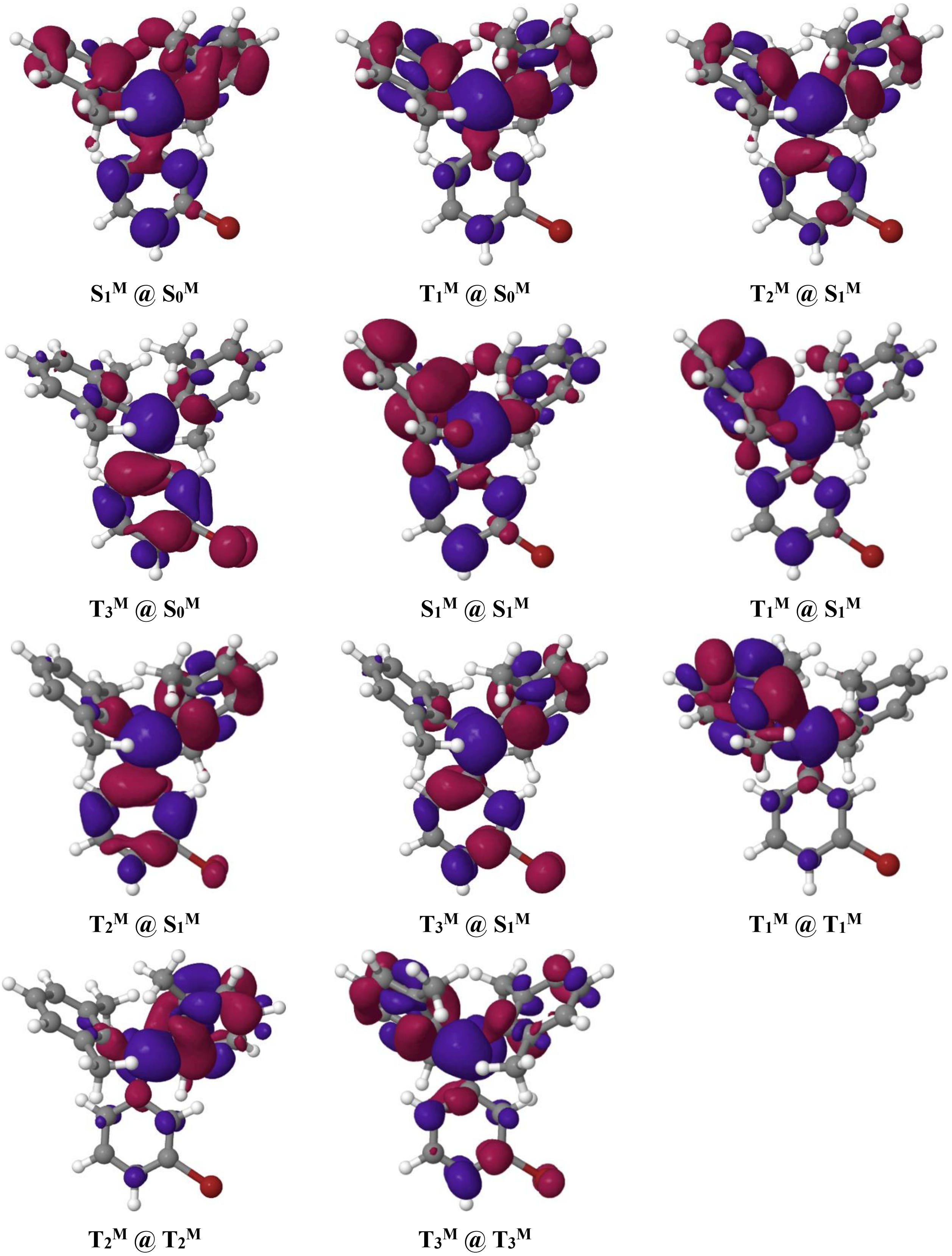

Figure S14. Difference densities of the S_1^M, T_1^M, T_2^M and T_3^M states of ***m*-BrTAB**. For color codes, see above.

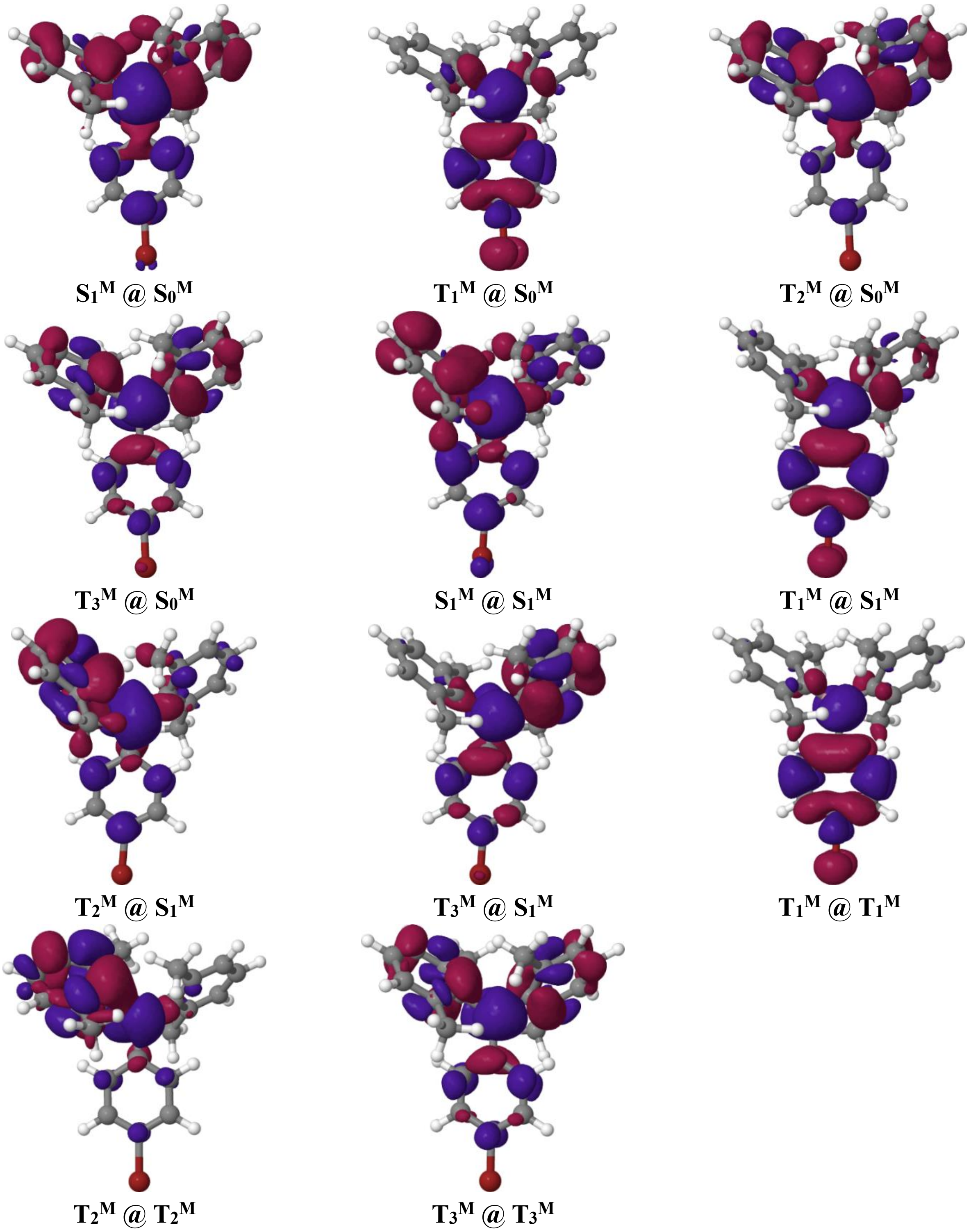

Figure S15. Difference densities of the S_1^M, T_1^M, T_2^M and T_3^M states of ***p*-BrTAB**. For color codes, see above

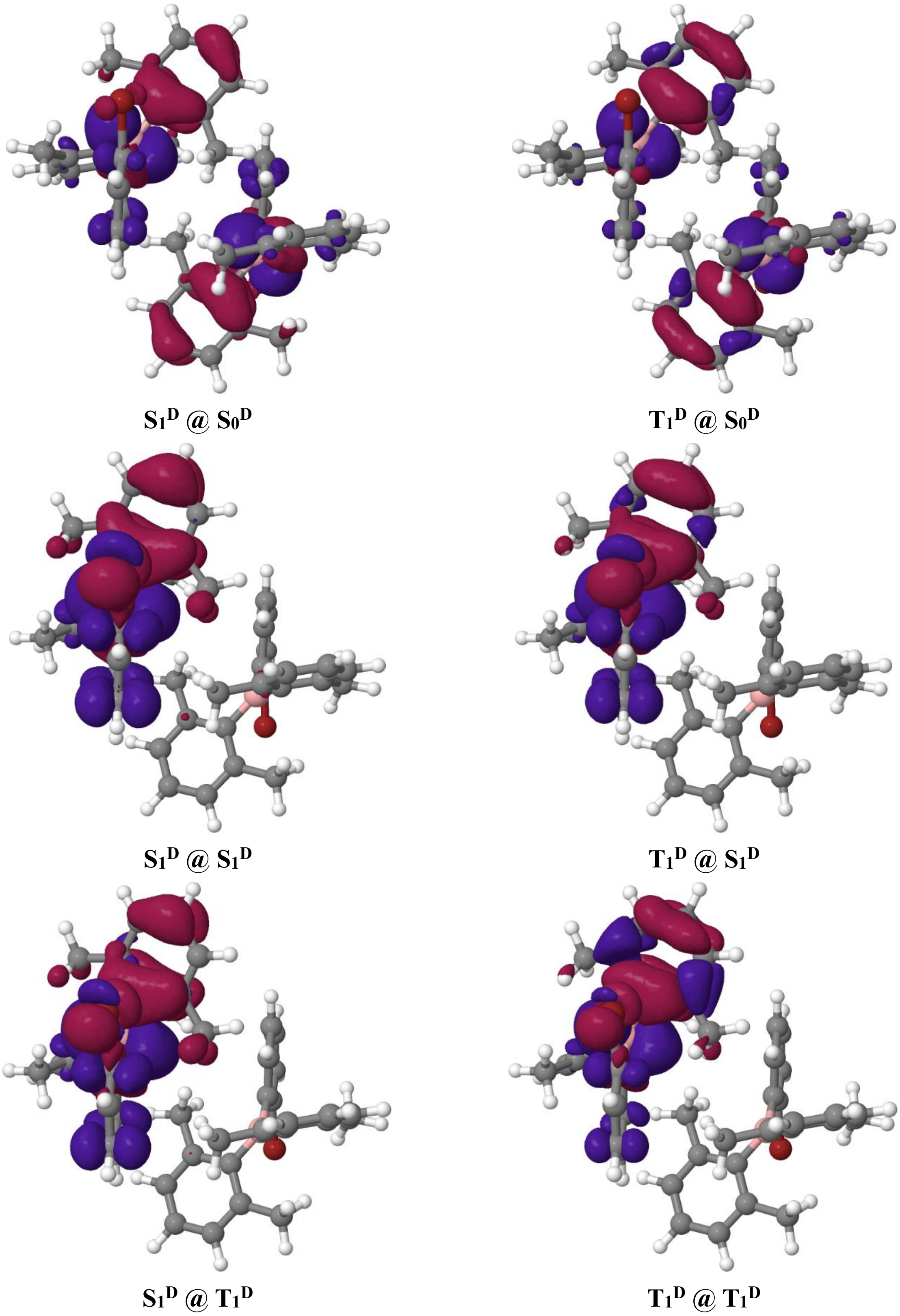

Figure S16. Difference densities of the S_1^D and T_1^D states of the ***o*-BrTAB** dimer. For color codes, see above.

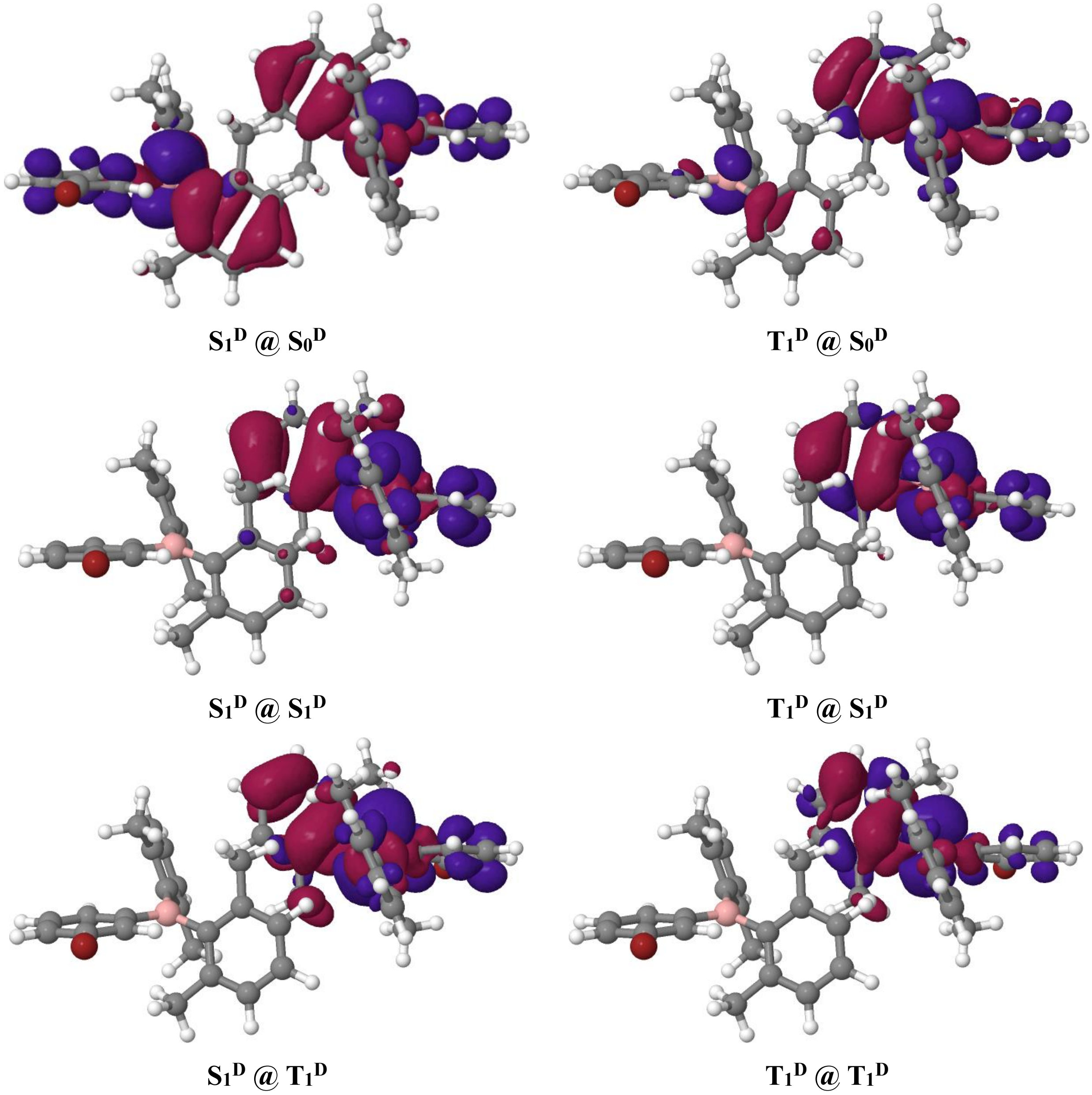

Figure S17. Difference densities of the S_1^D and T_1^D states of the ***m*-BrTAB** dimer. For color codes, see above.

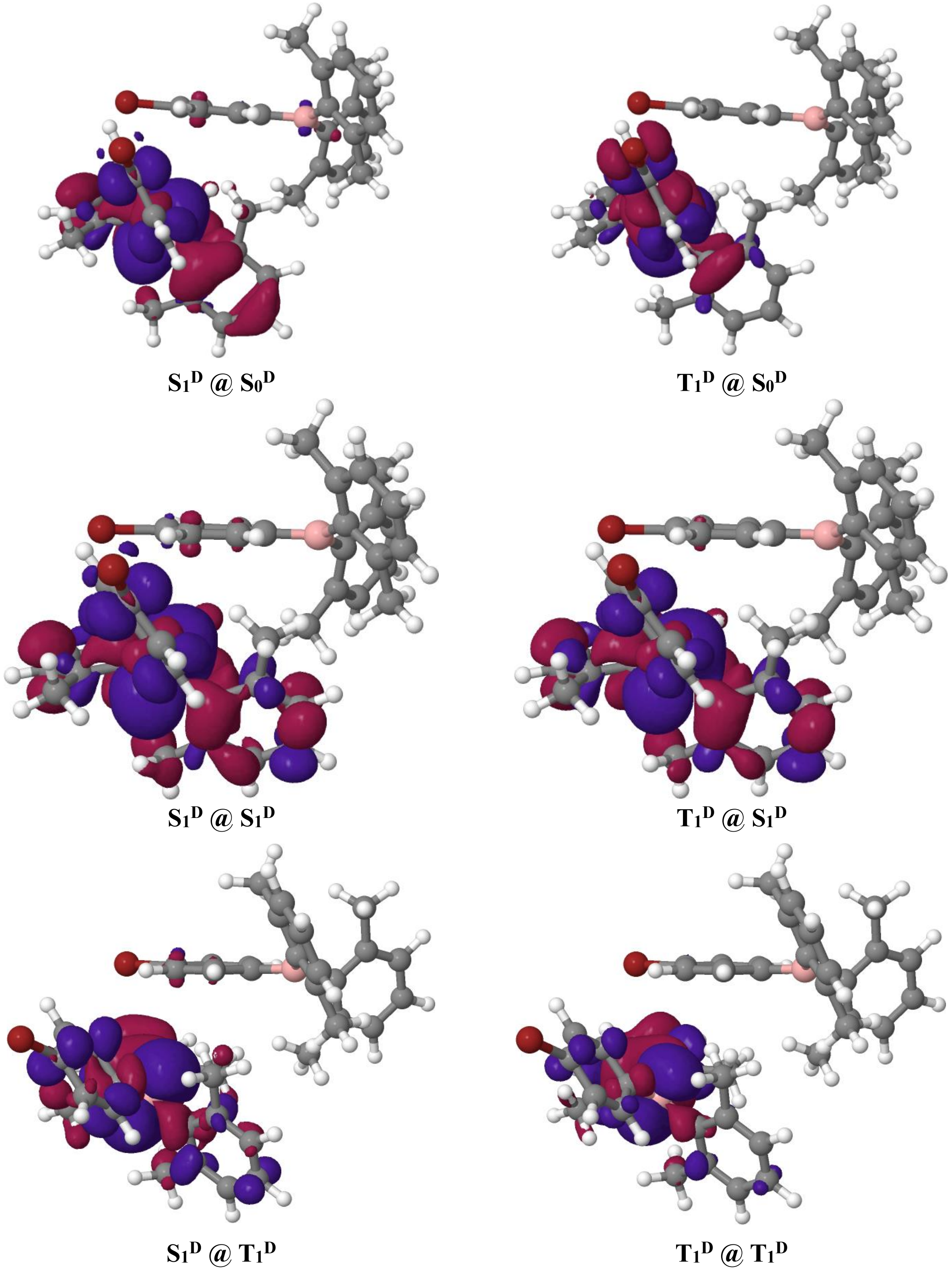

Figure S18. Difference densities of the S_1^D and T_1^D states of the ***p*-BrTAB** dimer. For color codes, see above.

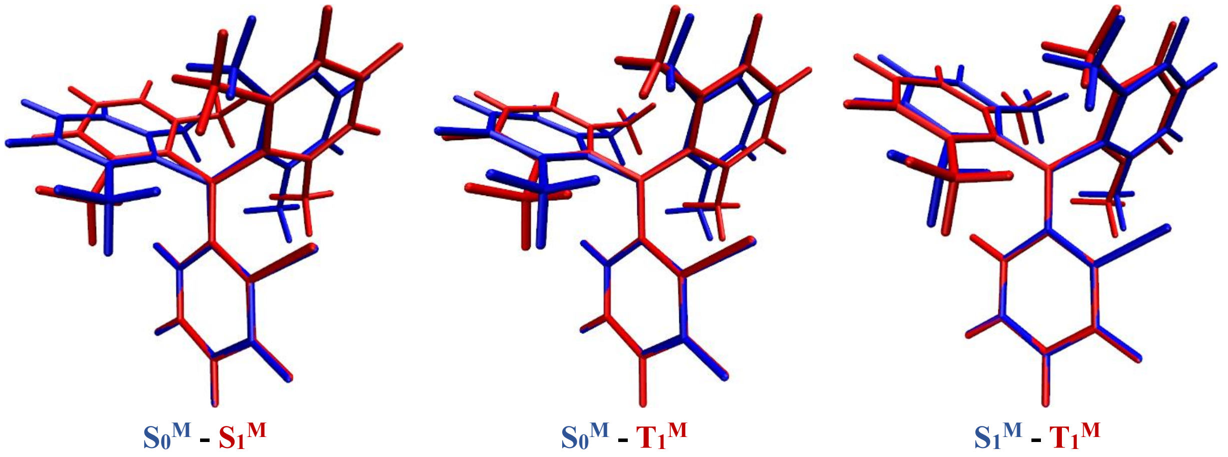

Figure S19. Structure overlays of ***o*-BrTAB**.

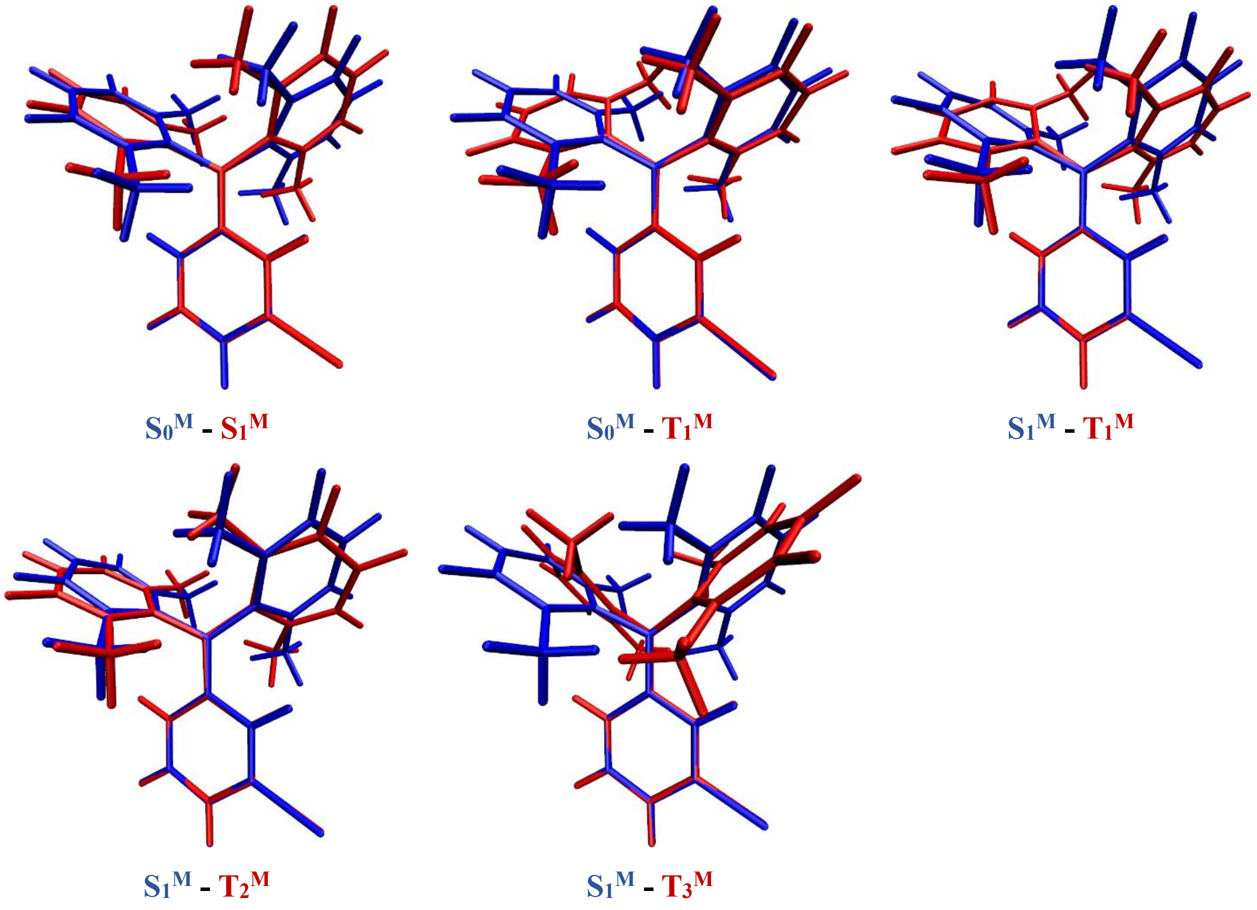

Figure S20. Structure overlays of ***m*-BrTAB***.*

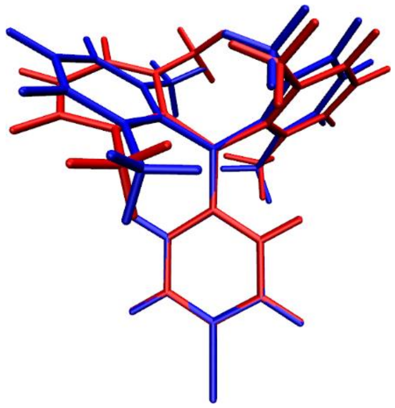

S_0^M - S_1^M

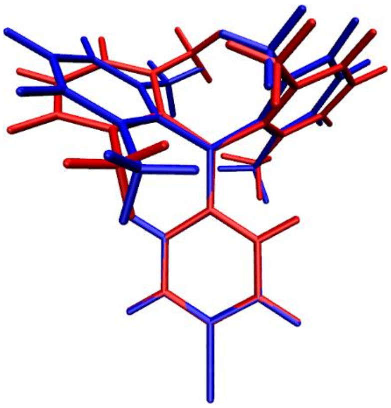

S_0^M - T_1^M

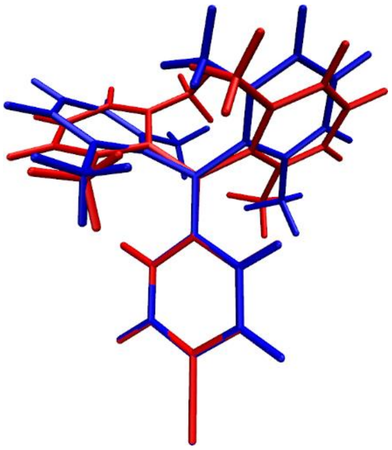

S_1^M - T_1^M

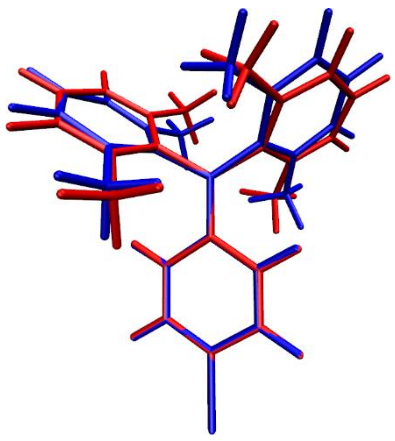

S_1^M - T_2^M

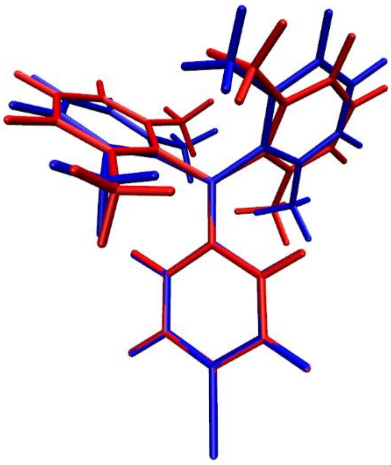

S_1^M - T_3^M

Figure S21. Structure overlays of ***p*-BrTAB**.

VI. Single-crystal X-ray diffraction

Table S9. Single-crystal X-ray diffraction data and structure refinements of ***o*-BrTAB**, ***m*-BrTAB** and ***p*-BrTAB** at 100 K.

Data	***o*-BrTAB**	***m*-BrTAB**	***p*-BrTAB**
CCDC number	2085814	2085815	2085816
Empirical formula	$C_{22}H_{22}BBr$	$C_{22}H_{22}BBr$	$C_{22}H_{22}BBr$
Formula weight / $g \cdot mol^{-1}$	377.11	377.11	377.11
T / K	100(2)	100(2)	100(2)
Radiation, λ / Å	Mo-Kα 0.71073	Mo-Kα 0.71073	Mo-Kα 0.71073
Crystal size / mm³	0.42×0.29×0.22	0.34×0.21×0.09	0.32×0.30×0.25
Crystal color, habit	colorless block	colorless plate	colorless block
μ / mm^{-1}	2.235	2.230	2.213
Crystal system	Monoclinic	Triclinic	Monoclinic
Space group	$P2_1/n$	$P\bar{1}$	$C2/c$
a / Å	8.1374(6)	7.559(3)	21.837(7)
b / Å	12.0614(9)	8.296(3)	18.956(5)
c / Å	18.7609(14)	15.372(6)	17.946(8)
α / °	90	102.84(3)	90
β / °	93.027(2)	94.23(2)	90.31(4)
γ / °	90	99.364(18)	90
Volume / Å^3	1838.8(2)	921.3(6)	7429(5)
Z	4	2	16
ρ_{calc} / $g \cdot cm^{-3}$	1.362	1.359	1.349
F(000)	776	388	3104
θ range / °	2.008 - 27.481	2.562 - 29.575	2.149 - 29.574
Reflections collected	36785	25129	95509
Unique reflections	4221	5190	10416
Parameters / restraints	221 / 0	221 / 0	441 / 0
GooF on F^2	1.060	1.059	1.019
R_1 [I>2σ(I)]	0.0350	0.0422	0.0306
wR^2 (all data)	0.0973	0.1117	0.0776
Max. / min. residual electron density / $e \cdot Å^{-3}$	1.278 / –0.548	2.049 / –0.380	0.439 / –0.594

Table S10. Single-crystal X-ray diffraction data and structure refinements of ***o*-BrTAB**, ***m*-BrTAB** and ***p*-BrTAB** at ambient temperature.

Data	***o*-BrTAB**	***m*-BrTAB**	***p*-BrTAB**
CCDC number	2089473	2118234	2118235
Empirical formula	$C_{22}H_{22}BBr$	$C_{22}H_{22}BBr$	$C_{22}H_{22}BBr$
T / K	290(2)	296(2)	300(2)
Radiation, λ / Å	Mo-Kα 0.71073	Mo-Kα 0.71073	Mo-Kα 0.71073
Crystal size / mm³	0.10×0.22×0.31	0.33×0.28×0.07	0.37×0.32×0.20
Crystal color, habit	Colorless block	Colorless plate	Colorless block
μ / mm^{-1}	2.178	2.162	2.213
Crystal system	Monoclinic	Triclinic	Monoclinic
Space group	$P2_1/n$	$P\bar{1}$	$C2/c$
a / Å	8.200(6)	7.6560(17)	21.9758(5)
b / Å	12.164(6)	8.3568(9)	19.2403(5)
c / Å	18.944(8)	15.496(3)	18.2089(3)
α / °	90	102.709(10)	90
β / °	93.277(7)	94.76(3)	91.0120(10)
γ / °	90	98.290(19)	90
Volume / Å^3	1886.4(17)	950.3(3)	7697.9(3)
Z	4	2	16
ρ_{calc} / g·cm^{-3}	1.328	1.318	1.302
F(000)	776	388	3104
θ range / °	1.991 - 26.372	1.357 – 26.370	2.394 – 26.371
Reflections collected	27916	25227	50647
Unique reflections	3859	3891	7870
Parameters / restraints	221 / 0	221 / 0	441 / 0
GooF on F^2	1.120	1.059	1.054
R_1 [I>2σ(I)]	0.0476	0.0489	0.0349
wR^2 (all data)	0.1197	0.1212	0.0990
Max. / min. residual electron density / e·Å^{-3}	0.373 / –0.579	0.590 / –0.512	0.412 / –0.716

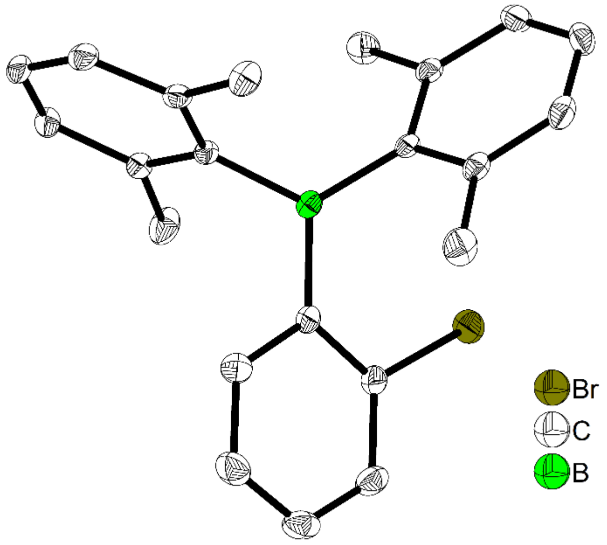

Figure S22. The solid-state molecular structure of ***o*-BrTAB** determined by single-crystal X-ray diffraction at 100 K. Ellipsoids are drawn at the 50% probability level, and H atoms are omitted for clarity.

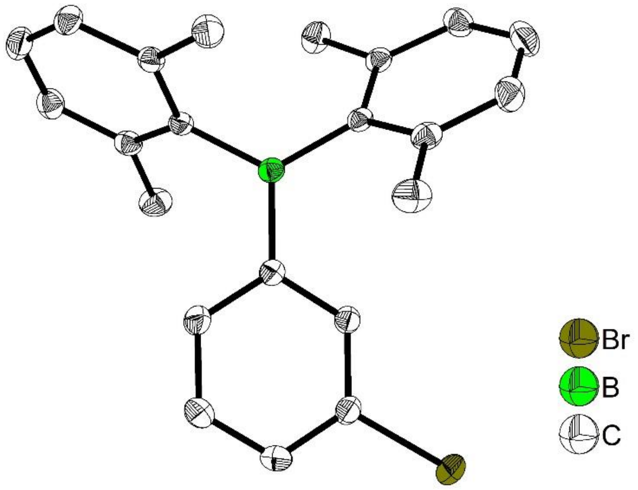

Figure S23. The solid-state molecular structure of ***m*-BrTAB** determined by single-crystal X-ray diffraction at 100 K. Ellipsoids are drawn at the 50% probability level, and H atoms are omitted for clarity.

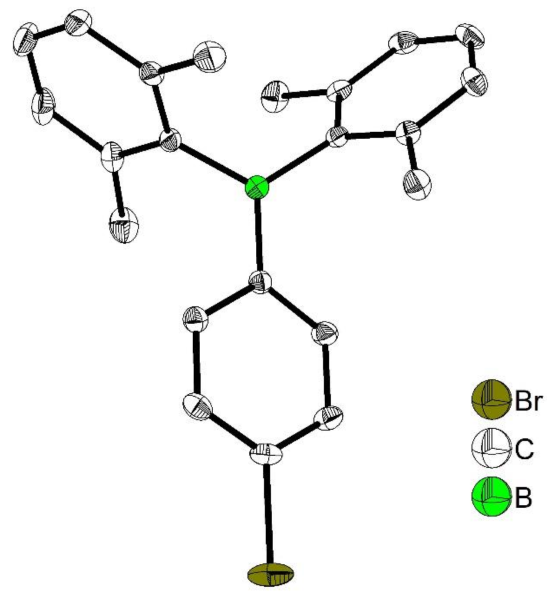

Figure S24. The solid-state molecular structure of ***p*-BrTAB** determined by single-crystal X-ray diffraction at 100 K. Ellipsoids are drawn at the 50% probability level, and H atoms are omitted for clarity. Only one of two non-symmetry-equivalent molecules is shown here.

Table S11. Selected bond lengths (Å), angles (°), and intramolecular contacts (Å) of ***o*-BrTAB**, ***m*-BrTAB** and ***p*-BrTAB** at 100 K. Aryl rings are numbered R1, R2, or R3 according to the C1, C7, or C15 atom being present and bonded to the boron atom, respectively.

	***o*-BrTAB**	***m*-BrTAB**	***p*-BrTAB**	
			Molecule 1	Molecule 2
C–Br	1.906(2)	1.899(2)	1.8982(16)	1.9007(17)
∠ C1–B–C15	121.38(18)	119.06(18)	119.58(14)	119.46(13)
∠ C1–B–C7	**116.28(18)**	118.00(18)	119.16(13)	122.29(13)
∠ C7–B–C15	122.15(19)	122.92(18)	121.26(13)	118.22(13)
Sum ∠ CBC	359.8(2)	360.0(2)	360.0(1)	360.0(1)
B–C1	1.578(3)	1.577(3)	**1.563(2)**	**1.561(2)**
B–C7	1.583(3)	1.579(3)	1.583(2)	1.585(2)
B–C15	1.574(3)	1.575(3)	1.581(2)	1.580(2)
∠ BC3 – aryl R1	38.61(9)	21.33(12)	20.06(8)	24.77(7)
∠ BC3 – aryl R2	61.02(6)	59.69(9)	63.38(7)	53.47(7)
∠ BC3 – aryl R3	55.21(9)	50.09(9)	57.88(7)	68.62(7)
∠ B–C1–C2	**127.2(2)**	121.18(18)	121.27(13)	122.85(14)
∠ B–C1–C6	**117.74(19)**	**117.47(19)**	121.34(14)	119.98(13)
∠ B–C15–C16	120.81(19)	121.33(18)	121.27(14)	120.93(14)
∠ B–C15–C20	120.93(19)	120.69(19)	120.78(14)	120.37(14)
∠ B–C7–C12	121.59(19)	121.47(18)	120.36(13)	120.77(13)
∠ B–C7–C8	119.71(19)	120.22(19)	120.88(14)	121.32(13)
Shortest B–Br contact	3.345(2)			
Shortest C15–Br contact	3.298(2)			

Table S12. Selected bond lengths (Å), angles (°), and intramolecular contacts (Å) of ***o*-BrTAB**, ***m*-BrTAB** and ***p*-BrTAB** at ambient temperature. Aryl rings are numbered R1, R2, or R3 according to the C1, C7, or C15 atom being present and bonded to the boron atom, respectively.

	***o*-BrTAB**	***m*-BrTAB**	***p*-BrTAB**	
T (K)	290(2)	296(2)	300(2)	
			Molecule 1	Molecule 2
C–Br	1.905(4)	1.896(3)	1.896(2)	1.899(2)
∠ C1–B–C15	121.3(4)	119.3(2)	119.35(18)	119.57(17)
∠ C1–B–C7	**116.1(3)**	117.4(3)	118.66(17)	121.78(17)
∠ C7–B–C15	122.4(3)	123.3(3)	121.99(18)	118.63(16)
Sum ∠ CBC	359.8(3)	360.0(3)	360.0(1)	360.0(2)
B–C1	1.578(5)	1.575(4)	**1.561(3)**	**1.560(3)**
B–C7	1.581(5)	1.584(4)	1.581(3)	1.587(3)
B–C15	**1.565(5)**	1.572(4)	1.583(3)	1.579(3)
∠ BC3 – aryl R1	39.27(2)	22.30(12)	22.15(8)	27.05(8)
∠ BC3 – aryl R2	60.38(1)	60.46(12)	63.22(8)	53.57(8)
∠ BC3 – aryl R3	54.78(2)	49.85(12)	57.06(8)	66.37(8)
∠ B–C1–C2	**127.2(3)**	121.5(3)	121.44(17)	122.74(17)
∠ B–C1–C6	**118.0(3)**	**117.4(3)**	121.83(17)	120.54(17)
∠ B–C15–C16	120.8(3)	121.3(3)	121.20(19)	120.93(18)
∠ B–C15–C20	121.6(3)	121.0(3)	120.83(18)	120.17(18)
∠ B–C7–C12	121.4(3)	121.3(3)	120.77(19)	121.04(17)
∠ B–C7–C8	120.2(3)	119.8(3)	120.5(2)	121.03(17)
Shortest B–Br contact	3.350(4)			
Shortest C15–Br contact	3.304(4)			

Table S13. Intermolecular C−H···C/Br, C···C/Br, and H···H interaction distances (Å) and angles (°) in ***o*-BrTAB**, ***m*-BrTAB** and ***p*-BrTAB** at 100 K.

Compound	C−H···C(Br)	H··· C(Br,H)	C···C(Br)	∠(CHC(Br))
***o*-BrTAB**	C4−H4···Br1	**3.1001(3)**	**3.354(2)**	97.16(15)
	C5−H5···C9	2.802(2)	3.618(3)	144.62(15)
	C19−H19···C3	2.802(2)	3.730(3)	165.82(15)
	C10−H10···C19	**2.753(2)**	3.488(3)	134.74(14)
	C17−H17···C10	2.800(2)	3.652(3)	149.78(15)
	C17−H17···C11	2.821(2)	3.768(3)	175.45(15)
	C14···C18 (methyl)		3.361(3)	
***m*-BrTAB**	C22(methyl)−H22B···C18	2.836(2)	3.676(4)	144.13(17)
	C9−H9···C16	2.861(2)	3.790(3)	166.26(15)
	C17−H17···C9	2.888(3)	3.699(4)	143.96(15)
	C14(methyl)−H14B···Br1	**2.9425(12)**	3.898(3)	165.05(14)
	C10−H10···Br1	**3.0346(13)**	3.736(3)	131.85(15)
***p*-BrTAB**	H11_1··· H11_1	2.3628(10)		
	H10_1··· H17_1	2.2659(5)		
	C2−H2_1···C4	2.8390(17)	3.752(2)	161.38(10)
	C3−H3···C9_1	2.821(2)	3.520(3)	131.16(11)
	C3−H3···C10_1	2.820(2)	3.686(3)	152.03(11)
	C9−H9···C16	2.8655(18)	3.783(3)	162.62(11)
	C13(methyl)−H13C_1···C17	2.8102(18)	3.653(3)	144.58(10)
	C3_1···Br1		**3.480(2)**	
	C14(methyl)−H14B_1···Br1	**2.9635(11)**	3.594(2)	123.11(10)

Table S14. Intermolecular C−H···C/Br, C···C/Br, and H···H interaction distances (Å) and angles (°) in ***o*-BrTAB**, ***m*-BrTAB** and ***p*-BrTAB** at ambient temperature. Significant intermolecular contacts as calculated below the sum of van der Waals radii using the Mercury program[24] are shown in black, while longer contacts, which do not play a significant role, are shown in bold for comparison with the close contacts at 100 K given in Table S13.

Compound	C−H···C(Br)	H··· C(Br,H)	C···C(Br)	∠(CHC(Br))
***o*-BrTAB 290(2) K**	C4−H4···Br1	3.1579(9)	**3.432(5)**	99.2(3)
	C5−H5···C9	**2.855(4)**	3.658(5)	145.3(2)
	C19−H19···C3	2.907(5)	3.809(6)	163.8(3)
	C10−H10···C19	**2.846(4)**	3.577(6)	136.4(2)
	C17−H17···C10	**2.873(4)**	3.709(6)	150.4(3)
	C17−H17···C11	**2.873(4)**	3.802(6)	177.2(3)
	C14···C18 (methyl)		3.426(6)	
***m*-BrTAB 296(2) K**	C22(methyl)−H22B···C18	2.981(4)	3.759(6)	139.0(3)
	C9−H9···C16	2.946(3)	3.858(4)	167.2(2)
	C17−H17···C9	2.980(3)	3.779(5)	144.9(2)
	C14(methyl)−H14B···Br1	**2.9857(8)**	3.927(3)	166.77(18)
	C10−H10···Br1	3.1583(6)	3.846(4)	132.3(3)
***p*-BrTAB 300(2) K**	H11_1··· H11_1	2.480(1)		
	H10_1··· H17_1	**2.393(1)**		
	C2−H2_1···C4	2.9083(19)	3.792(3)	159.20(14)
	C3−H3···C9_1	**2.887(2)**	3.585(3)	132.78(13)
	C3−H3···C10_1	2.913(2)	3.763(3)	152.60(13)
	C9−H9···C16	2.982(2)	3.879(4)	162.38(17)
	C9–H9···C17	2.935(2)	3.819(3)	159.24(16)
	C13(methyl)−H13C_1···C17	**2.870(2)**	3.699(3)	145.18(15)
	C3_1···Br1		3.611(3)	
	C14(methyl)−H14B_1···Br1	**2.9648(4)**	3.635(3)	127.98(15)

Table S15. Aryl···aryl (π···π) distances (Å) and angles (°) in crystals of ***m*-BrTAB** at 100 K and 296 K: nearest-neighbour (nn) C···C distances, centroid-centroid distances, interplanar separations, shifts, and slip angles. Aryl rings are numbered R1 or R3 according to the C1 or C15 atom being present and bonding to the boron atom, respectively.

Compound	Aryl···Aryl	nn C···C	Centroid-centroid distance	Interplanar separation	Shift	Slip angle
***m*-BrTAB 100 K**	R1···R1	3.698(5)	4.285(2)	3.640(3)	2.261(4)	31.8
	R3···R3	3.637(5)	4.951(3)	3.544(4)	3.457(4)	44.3
***m*-BrTAB 296 K**	R1···R1	3.871(8)	4.455(3)	3.812(4)	2.305(6)	31.2
	R3···R3	3.750(7)	5.034(3)	3.645(6)	3.473(6)	43.6

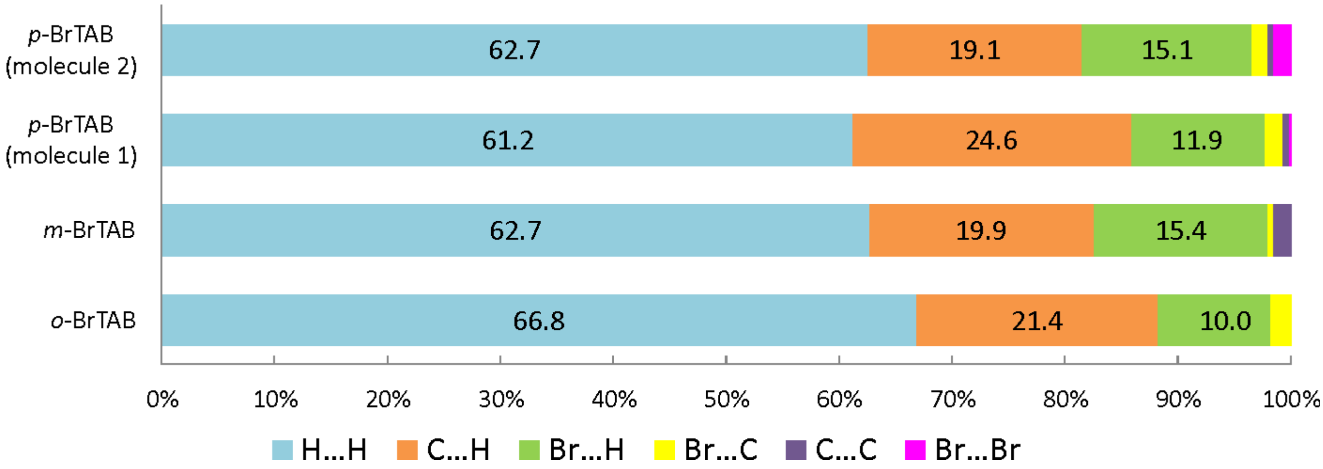

Figure. S25. Percentage contributions to the Hirshfeld surface area for the various close intermolecular contacts in ***o*-BrTAB**, ***m*-BrTAB**, and ***p*-BrTAB** at 100 K. In ***p*-BrTAB**, two symmetrically non-equivalent molecules are distinguished by slightly different contributions.

Table S16. Properties of crystals of ***o*-BrTAB**, ***m*-BrTAB** and ***p*-BrTAB** at ambient temperature and at 100 K: Volume of the molecule within van der Waals (V_m), Hirshfeld (V_H), and of the surface of the crystal voids (V_v), surface area of the molecule within van der Waals (SA_m), Hirshfeld (SA_H), and void surface area (SA_v), crystal packing coefficient (c_k), solvent accessible volume (V_{solv}), and percentage of intermolecular contacts. pfu…per formula unit; van der Waals radii used: C 1.7 Å, H: 1.09 Å, B: 2 Å, Br: 1.85 Å.

	***o*-BrTAB**		***m*-BrTAB**		***p*-BrTAB**			
**T (K)**	**290**	**100**	**296**	**100**	**Mol. 1 300 K**	**Mol. 2 300 K**	**Mol. 1 100 K**	**Mol. 2 100 K**
V_m / Å³	299.75	302.60	300.52	303.08	300.13	300.93	302.56	303.09
SA_m / Å²	344.28	347.42	345.67	348.27	346.83	346.31	350.12	348.78
V_H / Å³	464.68	452.75	467.53	453.02	472.65	474.25	455.65	457.64
SA_H / Å²	**367.41**	**364.05**	382.95	378.28	382.30	386.48	376.05	380.28
V_v / Å³	263.15	218.07	130.42	104.28	1174.41		912.57	
V_v / Å³ pfu	65.79	54.52	65.21	52.14	**73.40**		57.04	
SA_v / Å²	752.74	644.85	387.91	323.83	3275.62		2820.38	
SA_v / Å² pfu	**188.19**	161.21	193.96	161.92	204.73		176.27	
c_k	0.635	0.658	0.633	0.658	0.624		0.653	
V_{solv} / Å³	**31.9 (1.7%)**	0.0 (0%)	**41.9 (4.4%)**	16.2 (1.8%)	**209.2 (2.7%)**		39.3 (0.5%)	
Br···Br /%	0	0	0	0	0.3	1.8	0.2	1.6
Br···C / %	1.7	1.8	0.4	0.5	1.4	1.4	1.5	1.4
Br···H / %	10.2	10.0	15.6	15.4	12.1	15.5	11.9	15.1
C···C / %	0	0	**1.4**	**1.6**	0.4	0.4	0.6	0.5
C···H / %	20.4	21.4	18.9	19.9	23.5	18.1	24.6	19.1
H···H / %	**67.7**	**66.8**	63.6	62.7	62.4	62.8	61.2	62.3

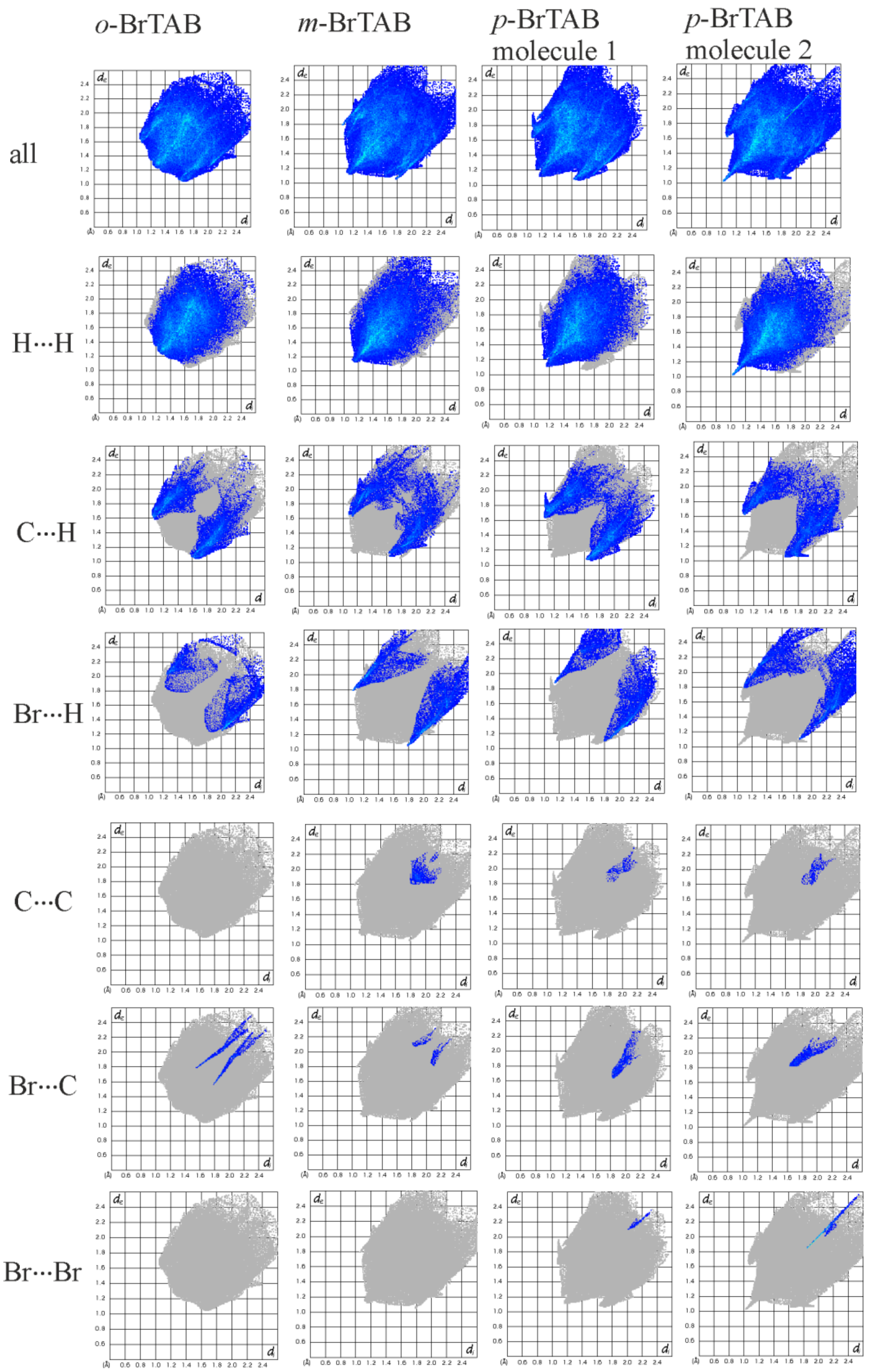

Figure S26. Two-dimensional fingerprint plots of molecules ***o*-BrTAB**, ***m*-BrTAB**, and ***p*-BrTAB** at 100 K calculated from the Hirshfeld surfaces. In ***p*-BrTAB**, two non-equivalent molecules are distinguished. The top row shows the complete fingerprint plots, while the other plots indicate the contributions of the individual intermolecular interactions (H···H, C···H, Br···H, C···C, Br···C, and Br···Br from top to bottom) within the grey area of all contributions.

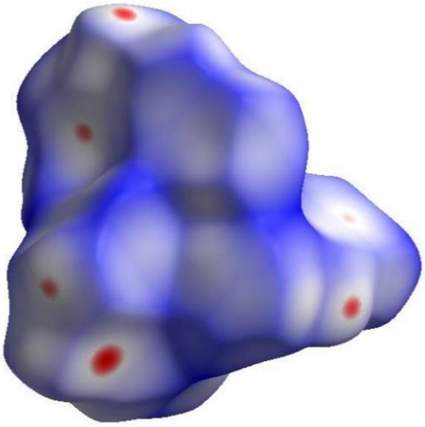

Figure S27. Hirshfeld surface of ***o*-BrTAB** at 100 K mapped with d_{norm} over the range -0.113 to 1.476. Close contacts are shown in red on the surface.

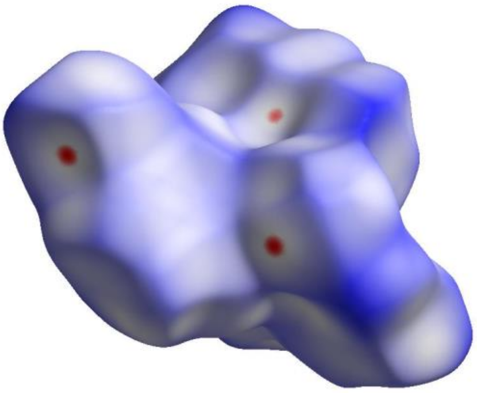

Figure S28. Hirshfeld surface of ***m*-BrTAB** at 100 K mapped with d_{norm} over the range -0.113 to 1.476. Close contacts are shown in red on the surface.

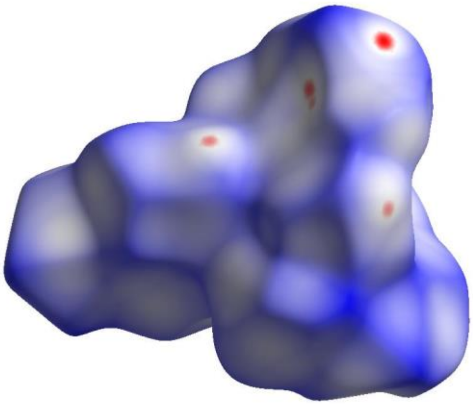

Figure S29. Hirshfeld surface of ***p*-BrTAB** at 100 K mapped with d_{norm} over the range -0.102 to 1.532. Close contacts are shown in red on the surface.

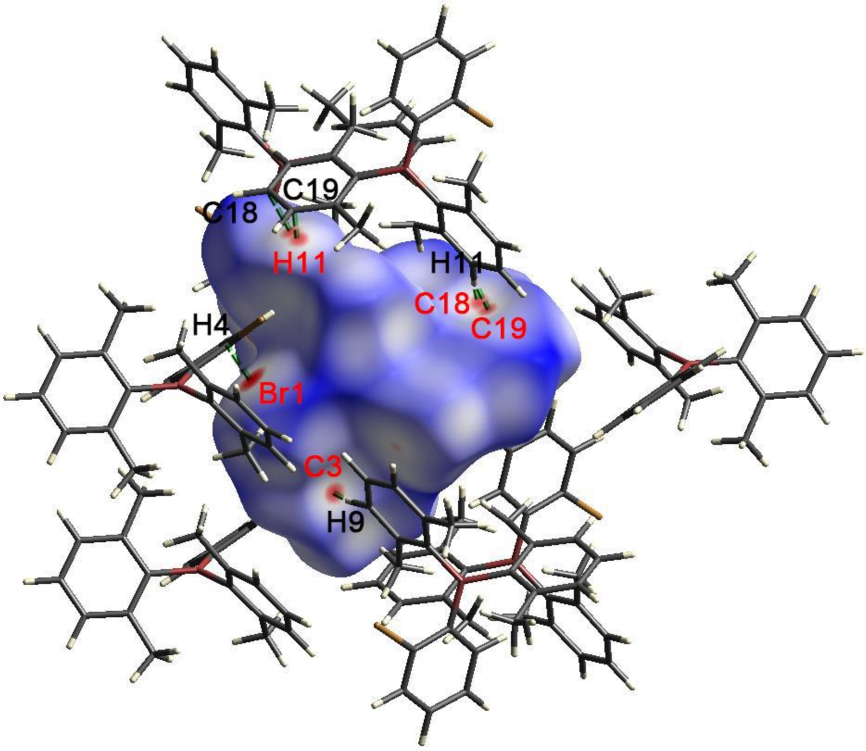

Figure S30. Hirshfeld surface of ***o*-BrTAB** mapped with d_{norm} over the range -0.113 to 1.476 at 100 K. Neighboring molecules associated with close contacts are shown.

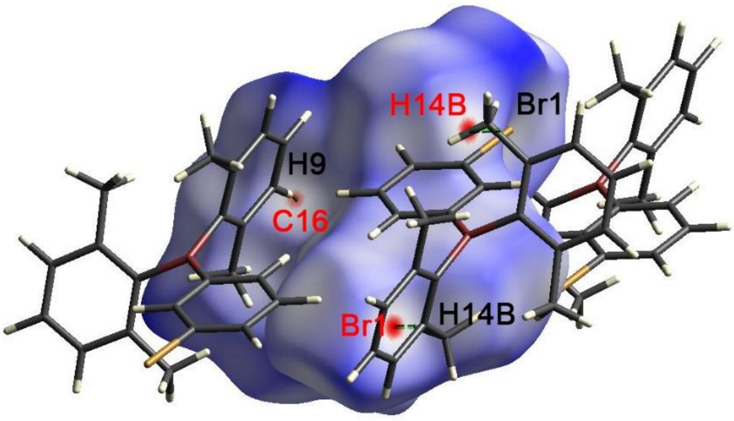

Figure S31. Hirshfeld surface of ***m*-BrTAB** mapped with d_{norm} over the range -0.113 to 1.476 at 100 K. Neighboring molecules associated with close contacts are shown.

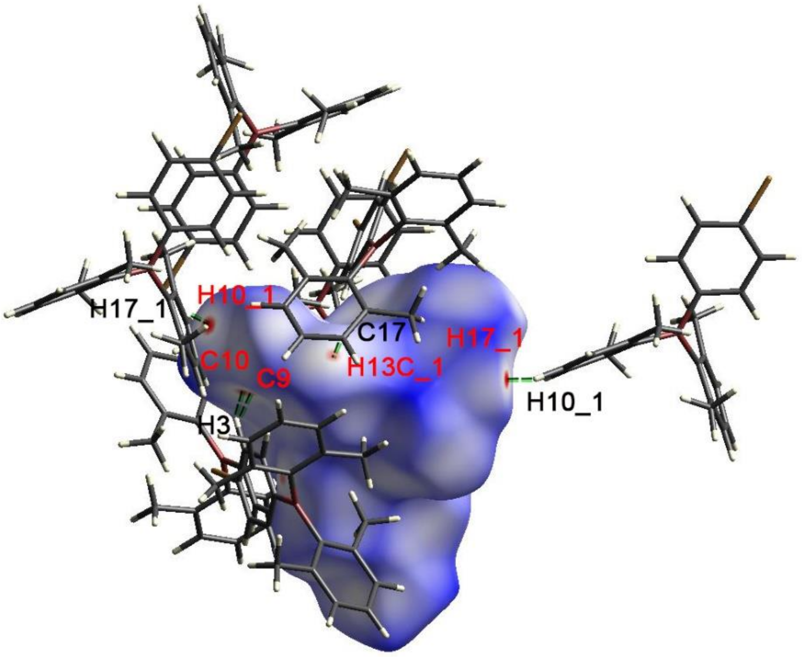

Figure S32. Hirshfeld surface of ***p*-BrTAB** mapped with d_{norm} over the range -0.102 to 1.532 at 100 K. Neighboring molecules associated with close contacts are shown.

VII. ^{1}H, ^{13}C, ^{11}B, GC-MS and HRMS spectra

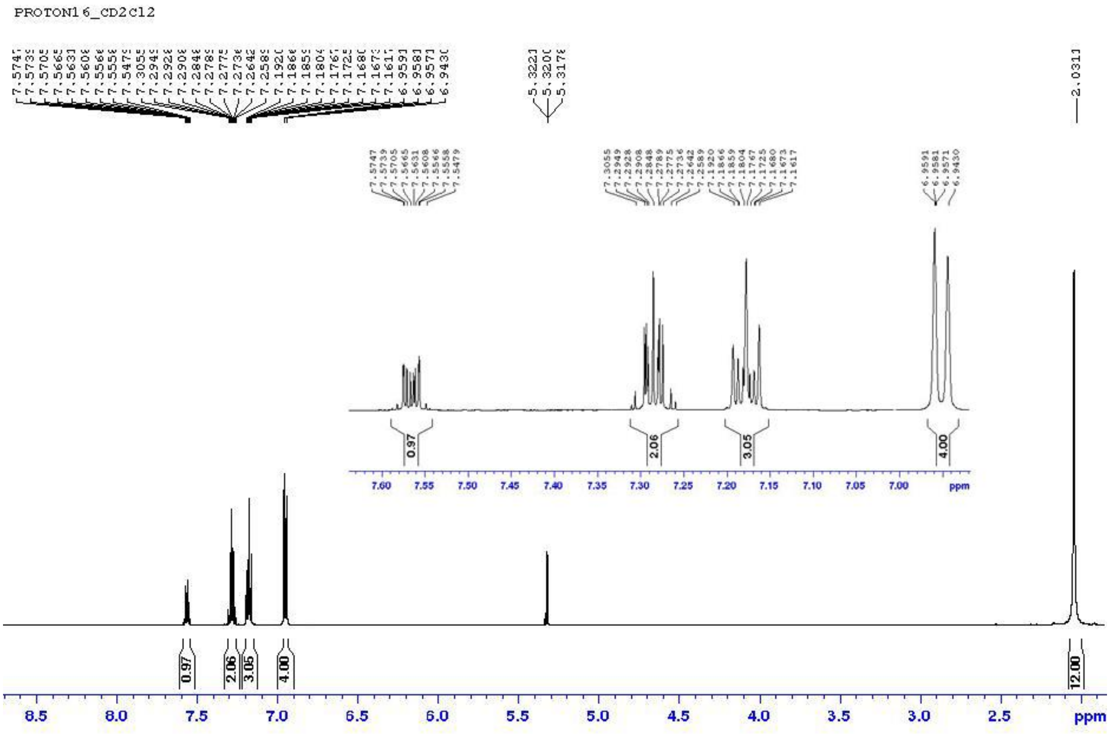

Figure S33. ^{1}H NMR spectrum (500 MHz, CD_2Cl_2) of ***o*-BrTAB**.

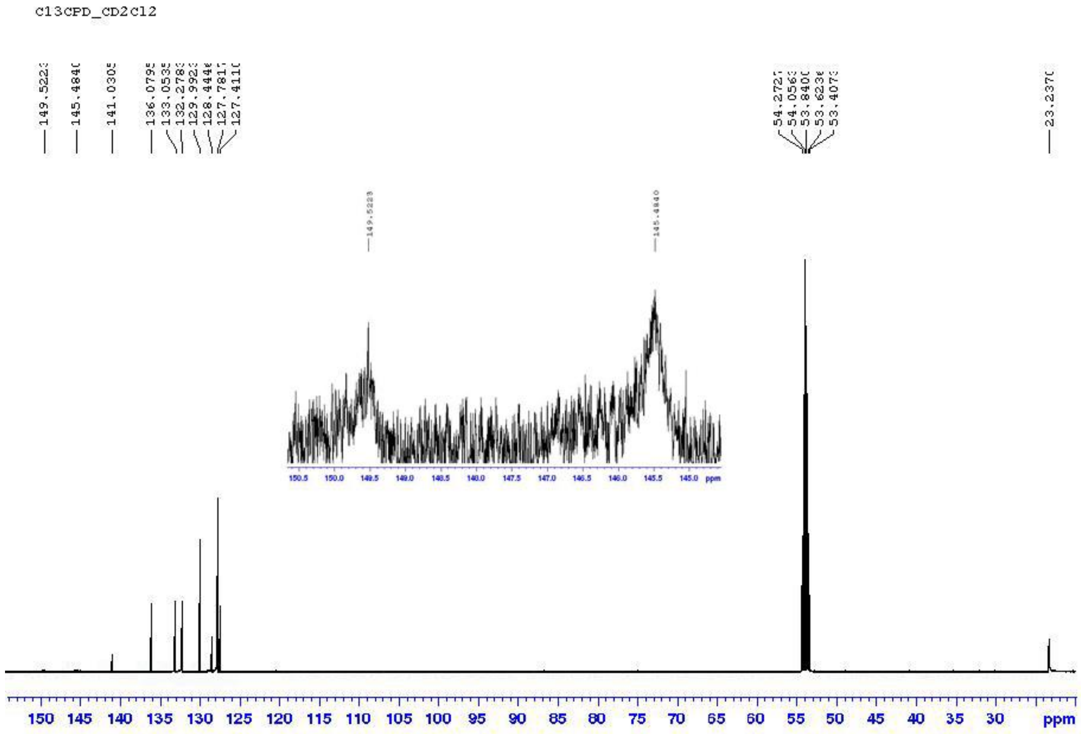

Figure S34. $^{13}C\{^{1}H\}$ NMR spectrum (126 MHz, CD_2Cl_2) of ***o*-BrTAB**.

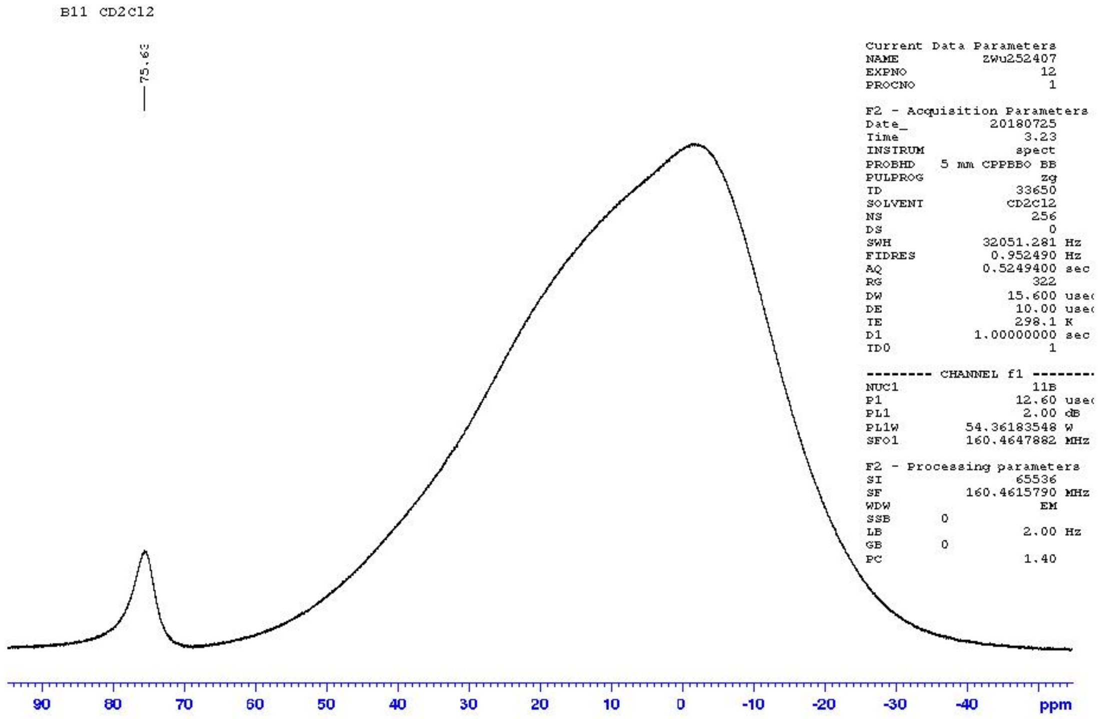

Figure S35. ^{11}B NMR spectrum (160 MHz, CD_2Cl_2) of ***o*-BrTAB**.

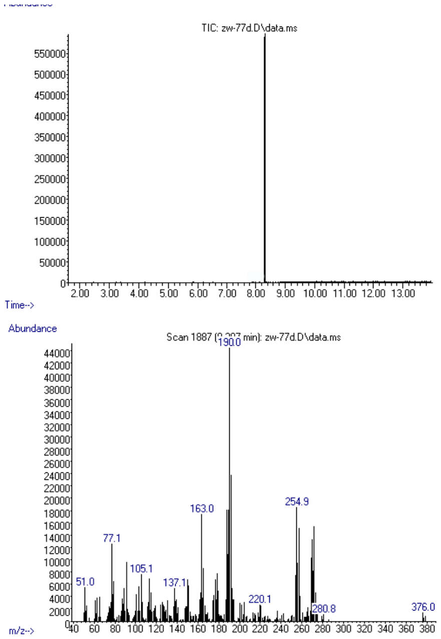

Figure S36. GC-MS total ion chromatogram^{TIC} and MS (EI)$_{m/z}$ of ***o*-BrTAB**.

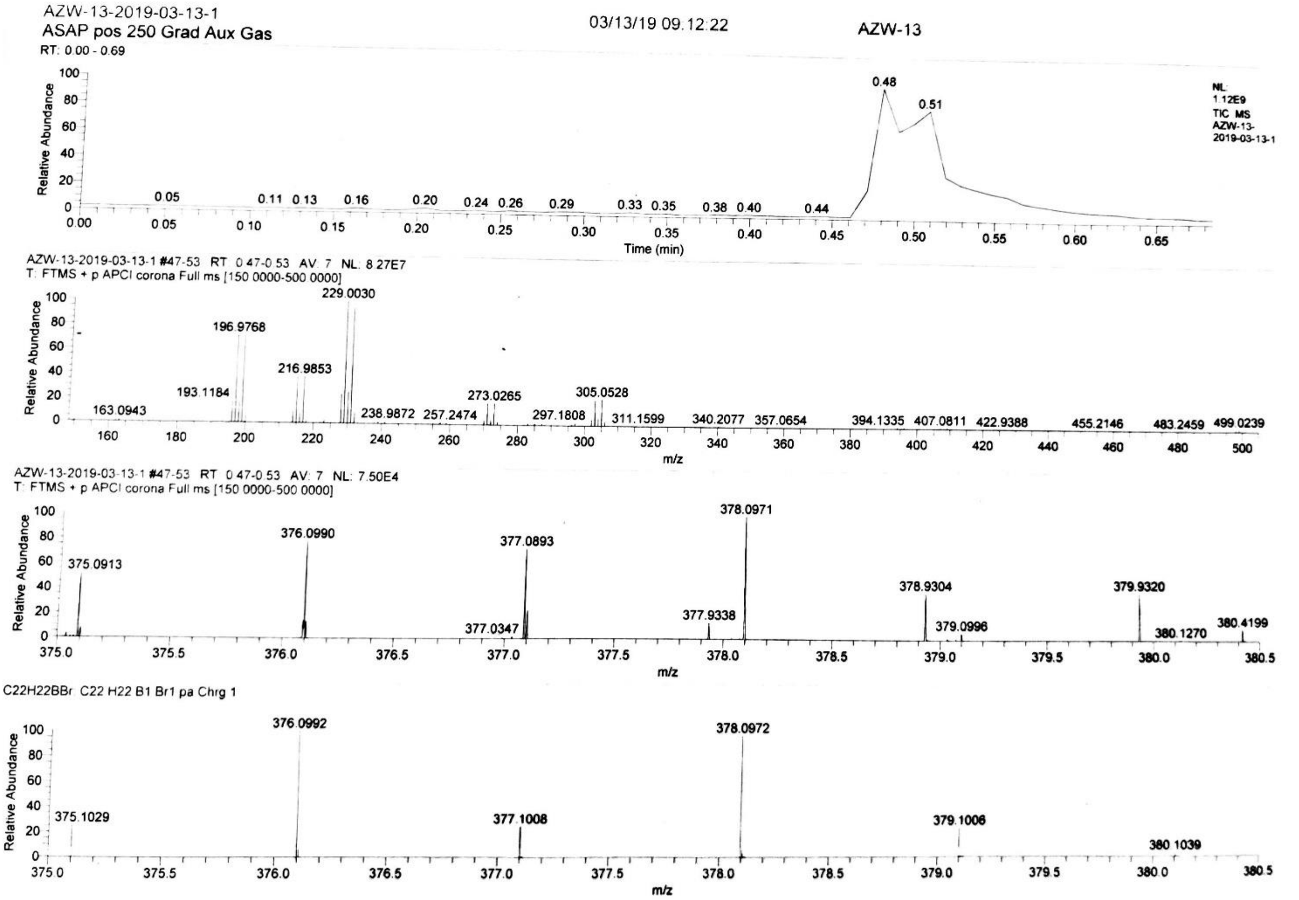

Figure S37. HR-ESI-MS spectrum of ***o*-BrTAB**.

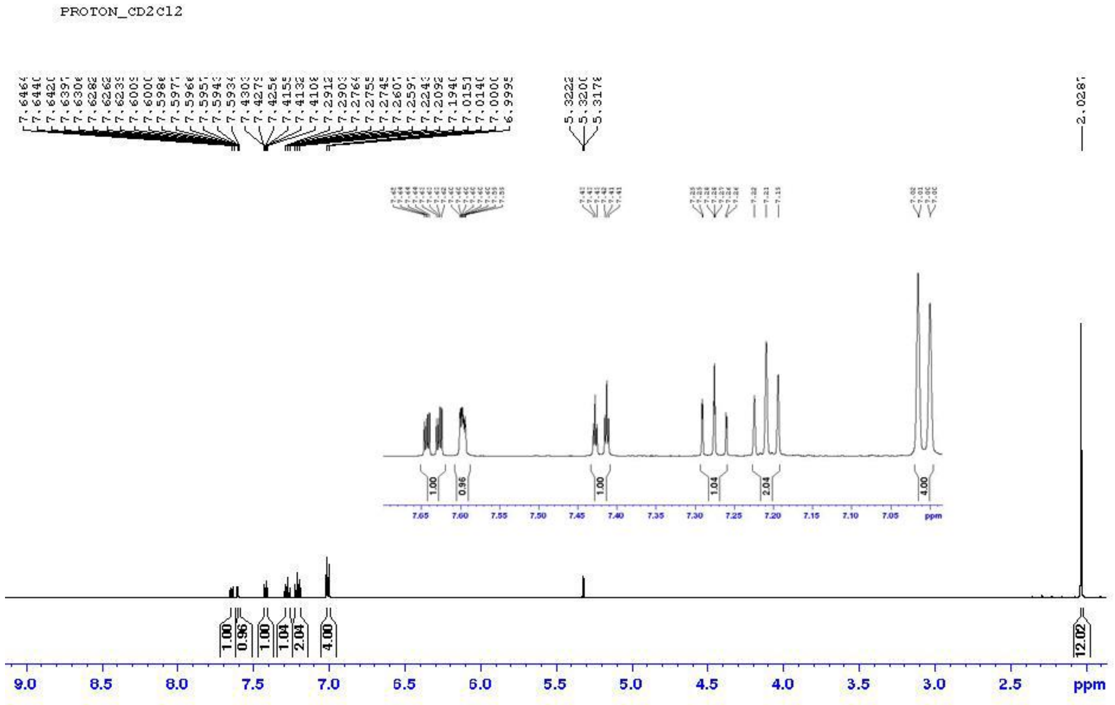

Figure S38. ^{1}H NMR spectrum (500 MHz, CD_2Cl_2) of ***m*-BrTAB**.

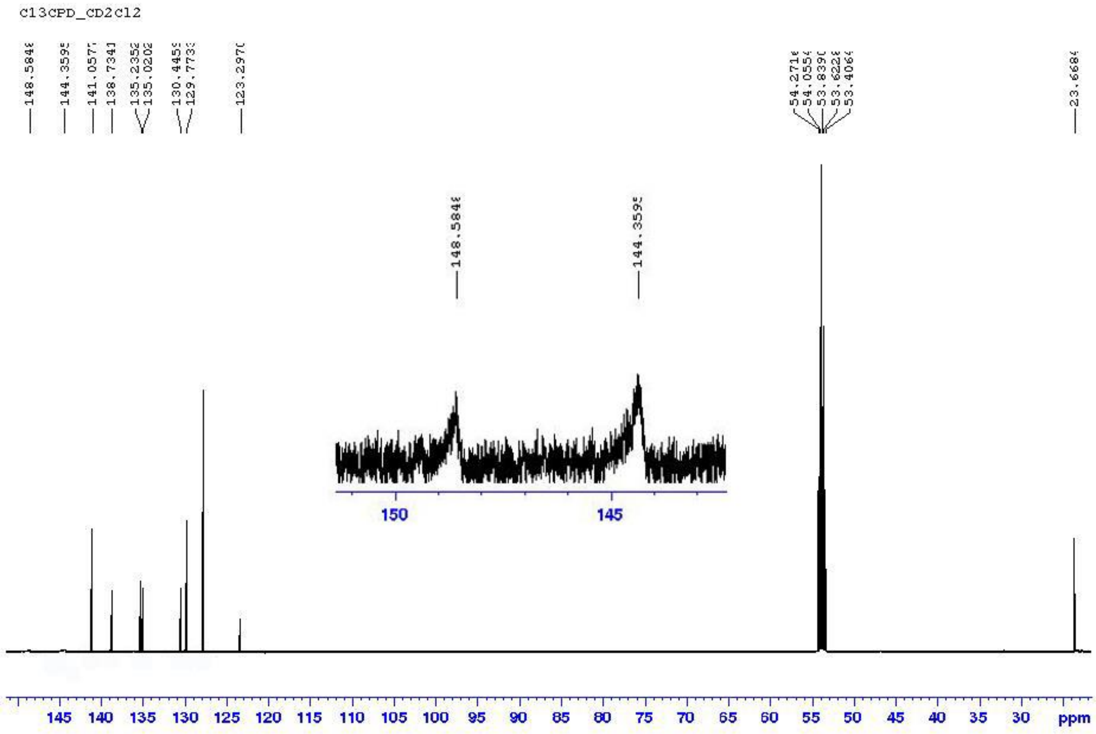

Figure S39. $^{13}C\{^{1}H\}$ NMR spectrum (126 MHz, CD_2Cl_2) of ***m*-BrTAB**.

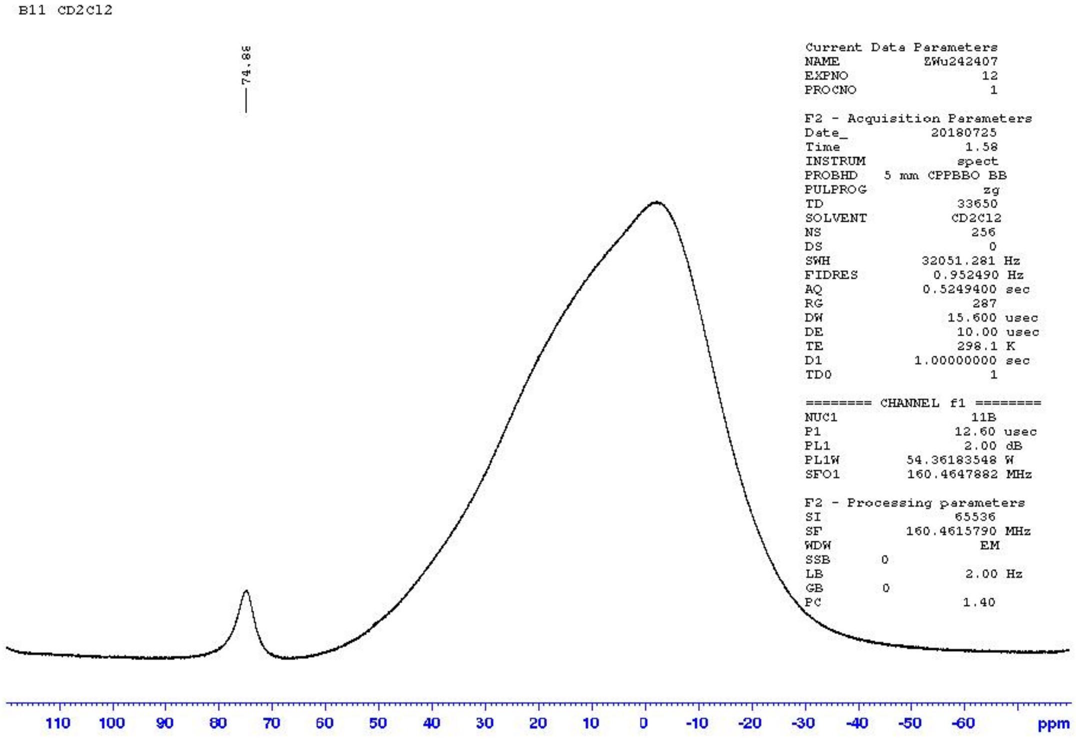

Figure S40. ^{11}B NMR spectrum (160 MHz, CD_2Cl_2) of ***m*-BrTAB**.

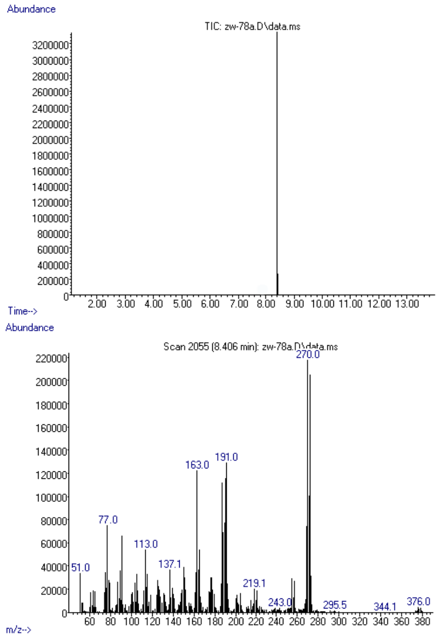

Figure S41. GC-MS total ion chromatogram^{TIC} and MS (EI)$_{m/z}$ of ***m*-BrTAB**.

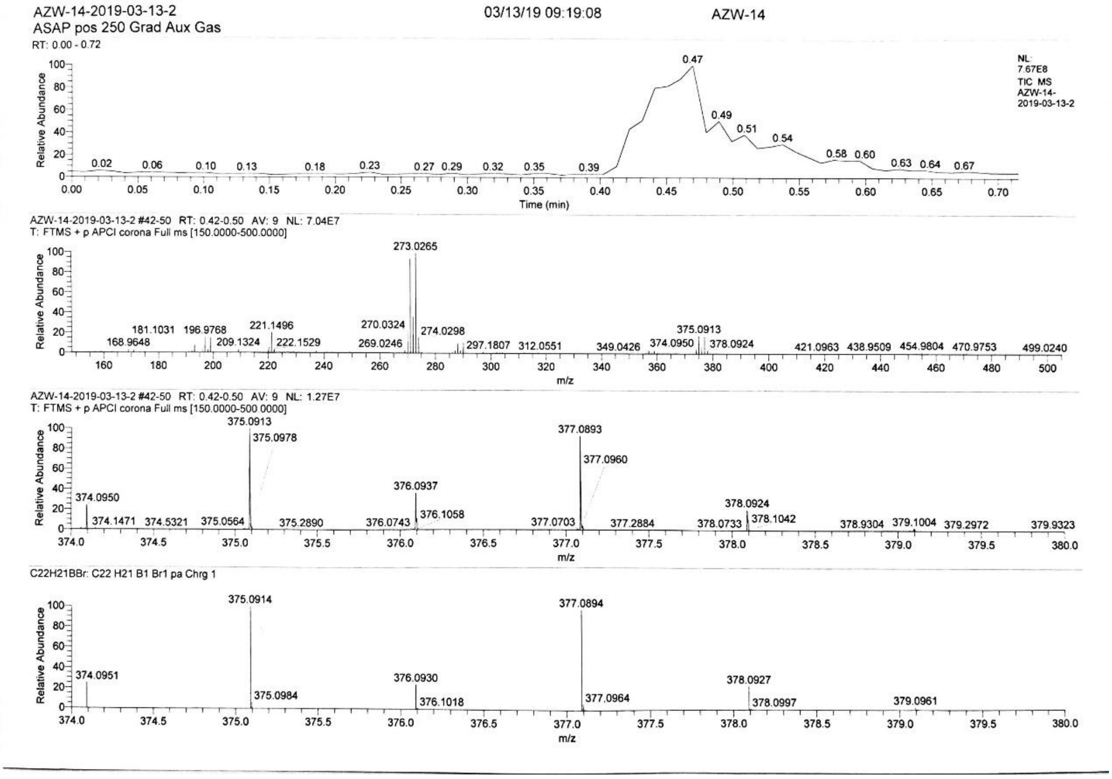

Figure S42. HR-ESI-MS spectrum of ***m*-BrTAB**.

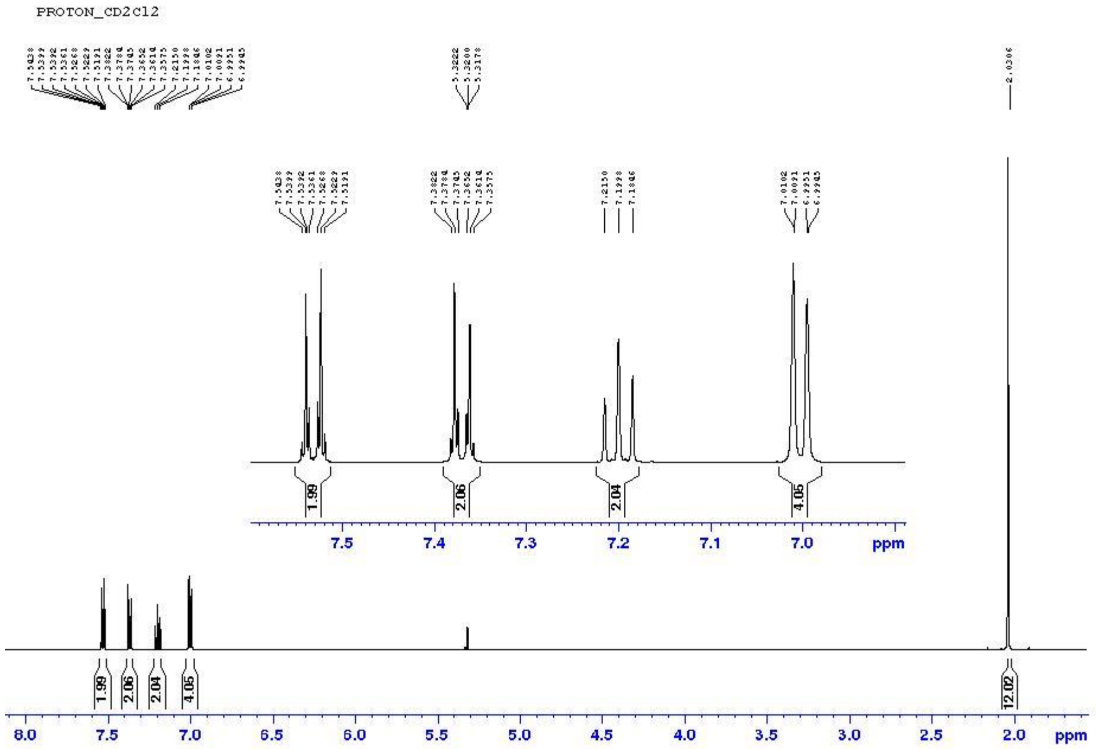

Figure S43. ^{1}H NMR spectrum (500 MHz, CD_2Cl_2) of ***p*-BrTAB**.

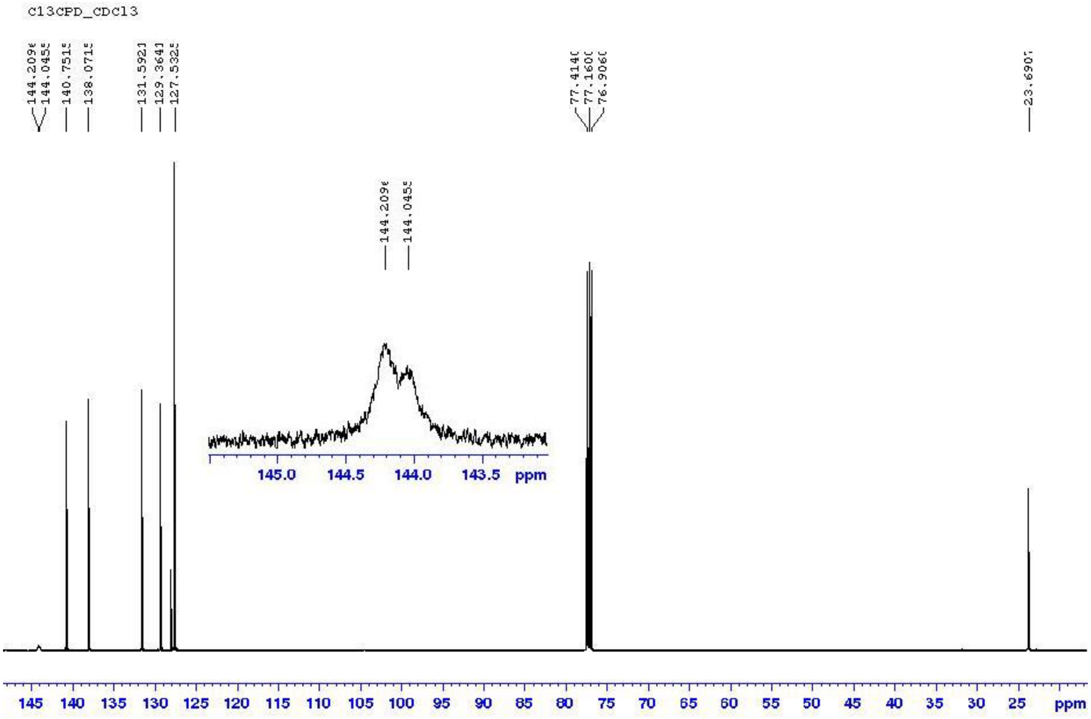

Figure S44. $^{13}C\{^{1}H\}$ NMR spectrum (126 MHz, $CDCl_3$) of ***p*-BrTAB**.

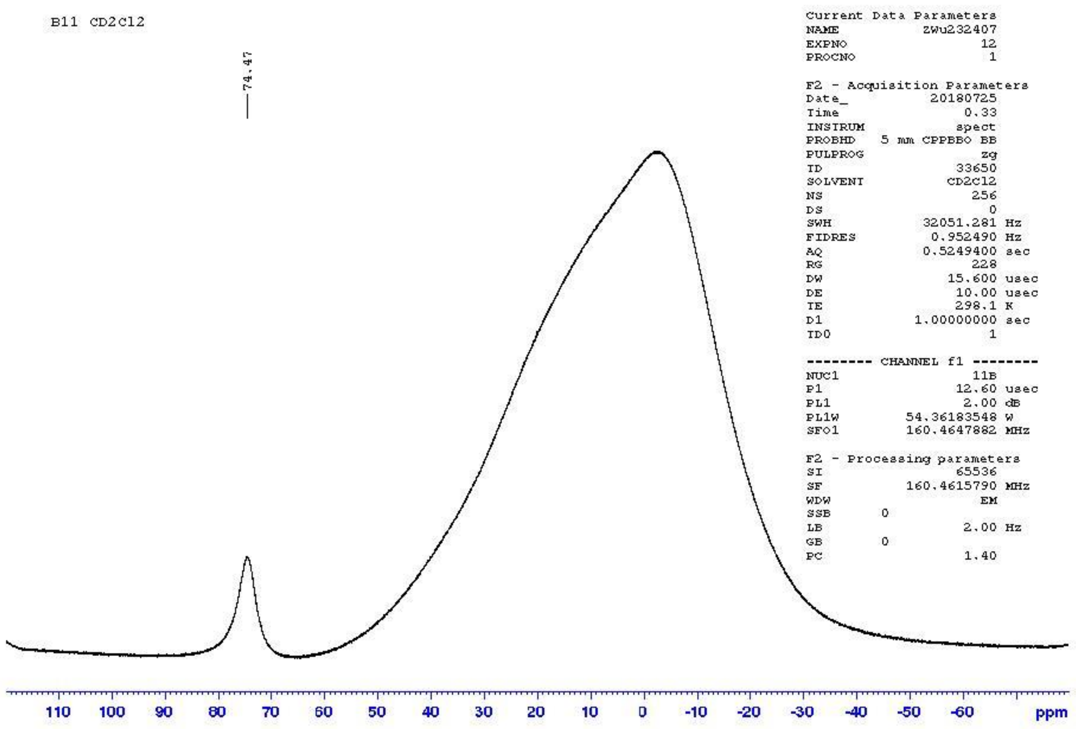

Figure S45. ^{11}B NMR spectrum (160 MHz, CD_2Cl_2) of ***p*-BrTAB**.

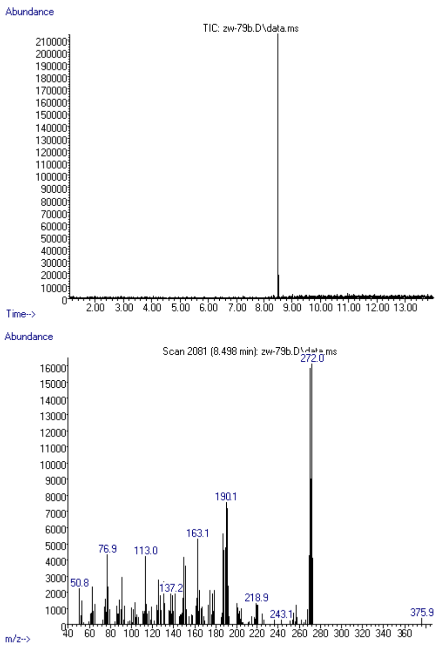

Figure S46. GC-MS total ion chromatogram^{TIC} and MS $(EI)_{m/z}$ of ***p*-BrTAB**.

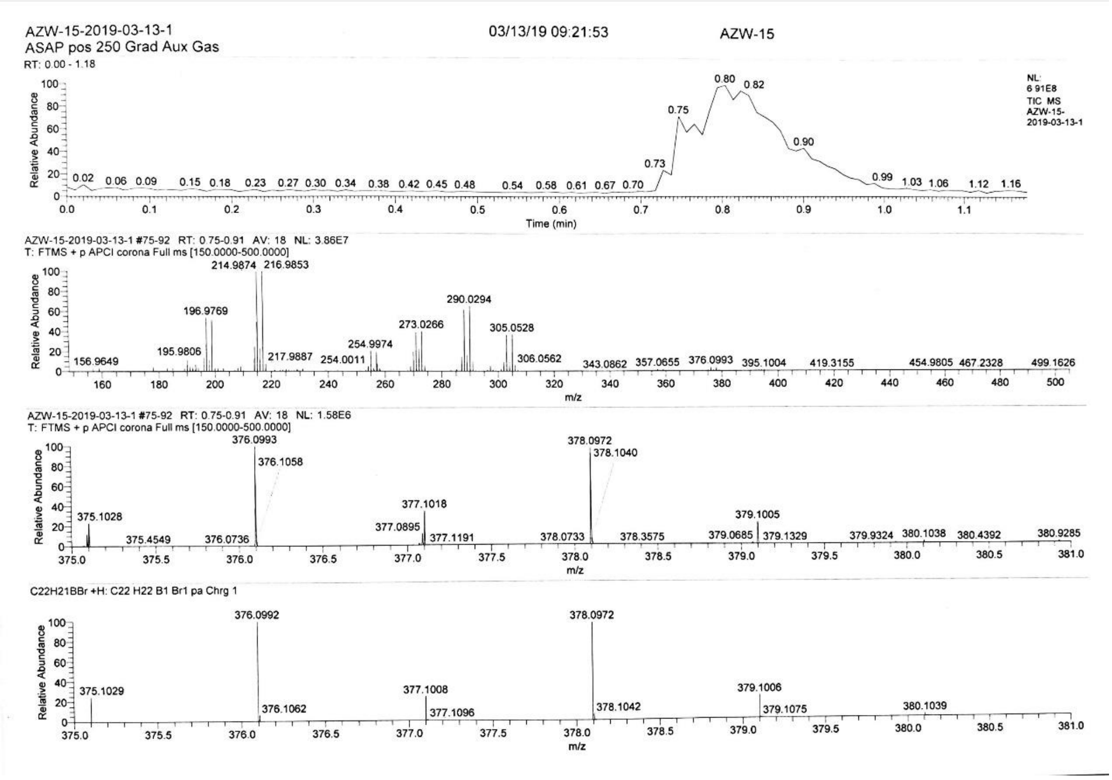

Figure S47. HR-ESI-MS spectrum of ***p*-BrTAB**.

VIII. References

[1] B. H. Toby, *J. Appl. Crystallogr.* **2005**, *38*, 1040-1041.

[2] A. C. Larson, R. B. Von Dreele, Los Alamos National Laboratory Report LAUR, **2004**, pp. 86-748.

[3] TURBOMOLE V7.0 2015, A development of University of Karlsruhe and Forschungszentrum Karlsruhe GmbH, 1989-2007, TURBOMOLE GmbH, since 2007, available from http://www.turbomole.com.

[4] Gaussian 98 g09, Revision E.01, M. J. Frisch, G. W. Trucks, H. B. Schlegel, G. E. Scuseria, M. A. Robb, J. R. Cheeseman, G. Scalmani, V. Barone, B. Mennucci, G. A. Petersson, H. Nakatsuji, M. Caricato, X. Li, H. P. Hratchian, A. F. Izmaylov, J. Bloino, G. Zheng, J. L. Sonnenberg, M. Hada, M. Ehara, K. Toyota, R. Fukuda, J. Hasegawa, M. Ishida, T. Nakajima, Y. Honda, O. Kitao, H. Nakai, T. Vreven, J. A. Montgomery, Jr., J. E. Peralta, F. Ogliaro, M. Bearpark, J. J. Heyd, E. Brothers, K. N. Kudin, V. N. Staroverov, T. Keith, R. Kobayashi, J. Normand, K. Raghavachari, A. Rendell, J. C. Burant, S. S. Iyengar, J. Tomasi, M. Cossi, N. Rega, J. M. Millam, M. Klene, J. E. Knox, J. B. Cross, V. Bakken, C. Adamo, J. Jaramillo, R. Gomperts, R. E. Stratmann, O. Yazyev, A. J. Austin, R. Cammi, C. Pomelli, J. W. Ochterski, R. L. Martin, K. Morokuma, V. G. Zakrzewski, G. A. Voth, P. Salvador, J. J. Dannenberg, S. Dapprich, A. D. Daniels, O. Farkas, J. B. Foresman, J. V. Ortiz, J. Cioslowski, and D. J. Fox, Gaussian, Inc., Wallingford CT, 2013.

[5] J. P. Perdew, M. Ernzerhof and K. Burke, *J. Chem. Phys*. **1996**, *105*, 9982–9985.

[6] J. P. Perdew, K. Burke and M. Ernzerhof, *Phys. Rev. Lett*. **1996**, *77*, 3865–3868.

[7] C. Adamo and V. Barone, *J. Chem. Phys*. **1999**, *110*, 6158–6170.

[8] K. Eichkorn, F. Weigend, O. Treutler, R. Ahlrichs, *Theor. Chem. Acc.*, **1997**, 97, 119.

[9] K.A. Peterson, D. Figgen, E. Goll, H. Stoll and M. Dolg, *J. Chem. Phys.*, **2003**, 119, 11113.

[10] S. Grimme, Mirko Waletzke, J. Chem. Phys. **1999**, 111, 5645-5655.

[11] C. M. Marian, A. Heil and M. Kleinschmidt, *WIREs Comput. Mol. Sci*. **2019**, 9, e1394.

[12] I. Lyskov, M. Kleinschmidt, C. M. Marian, *J. Chem., Phys.*, **2016**, 144, 034104

[13] A. D. Becke, *J. Chem. Phys.*, **1993**, 98, 1372.

[14] C. Lee, W. Yang, and R. G. Parr, *Phys. Rev. B* **1988**, 37, 785.

[15] M. Kleinscmidt, J. Tatchen and C. M. Marian, *J. Chem. Phys*. **2006**, *124*, 124101.

[16] M. Kleinschmidt and C. M. Marian, *Chem. Phys*. **2005**, *311*, 71-79.

[17] M. Kleinschmidt, J. Tatchen and C. M. Marian, *J. Comput. Chem*. **2002**, *23*, 824-833.

[18] M. Etinski, J. Tatchen and C. M. Marian, *J. Chem. Phys*. **2011**, *134*, 154105.

[19] M. Etinski, J. Tatchen and C. M. Marian, *Phys. Chem. Chem. Phys*. **2014**, *16*, 4740-4751.

[20] G. M. Sheldrick, *Acta Crystallogr. A Found Adv.* **2015**, *71*, 3-8.

[21] G. M. Sheldrick, *Acta Crystallogr. A* **2008**, *64*, 112-122.

[22] C. B. Hübschle, G. M. Sheldrick, B. Dittrich, *J. Appl. Crystallogr.* **2011**, *44*, 1281-1284.

[23] K. D. Brandenburg, Crystal and Molecular Structure Visualization, Crystal Impact, H. Putz & K. Brandenburg GbR, Bonn (Germany), 2017.

[24] C. F. Macrae, I. J. Bruno, J. A. Chisholm, P. R. Edgington, P. McCabe, E. Pidcock, L. Rodriguez-Monge, R. Taylor, J. van de Streek, P. A. Wood, *J. Appl. Crystallogr.* **2008**, *41*, 466-470.

[25] O. V. Dolomanov, L. J. Bourhis, R. J. Gildea, J. A. K. Howard, H. Puschmann, *J. Appl. Crystallogr.* **2009**, *42*, 339-341.

[26] P. R. Spackman, M. J. Turner, J. J. McKinnon, S. K. Wolff, D. J. Grimwood, D. Jayatilaka, M. A. Spackman, CrystalExplorer21 (2021), University of Western Australia, http://crystalexplorer.net.

[27] Y. A. Cho, D. S. Kim, H. R. Ahn, B. Canturk, G. A. Molander, J. Ham, *Org. Lett*. **2009**, *11*, 4330-4333.

[28] a) M. Ferger, S. M. Berger, F. Rauch, M. Schonitz, J. Ruhe, J. Krebs, A. Friedrich, T. B. Marder, *Chem. Eur. J.* **2021**,

27, 9094-9101; b) Z. Wu, J. Nitsch, J. Schuster, A. Friedrich, K. Edkins, M. Loebnitz, F. Dinkelbach, V. Stepanenko, F. Würthner, C. M. Marian, L. Ji, T. B. Marder, *Angew. Chem. Int. Ed*. **2020**, *59*, 17137-17144.
